# MXene Chemistries for Biomedicine: On-Body, In-Body, and Beyond

**DOI:** 10.1021/acsnano.6c05051

**Published:** 2026-08-24

**Authors:** Damla Alkaya, Roberta Cagliani, Gokce Yagmur Summak, Linda Giro, Omur Besbinar, Maha Alhosani, Maksym Pogorielov, Mehak Gupta, Sanjiv Dhingra, Marco Orecchioni, Flavia Vitale, Giacomo Chessa, Valentina Palmieri, Benjamin Chacon, Yury Gogotsi, Anna-Maria Pappa, Laura Fusco, Acelya Yilmazer, Lucia Gemma Delogu

**Affiliations:** † Integrated Technologies Research Center (BÜTAM), 37504 Ankara University, 06790 Ankara, Türkiye; ‡ Graduate School of Health Sciences, Ankara University, 06110 Ankara, Türkiye; § Department of Biological Sciences, Khalifa University of Science and Technology, Abu Dhabi 127788, United Arab Emirates; ∥ Department of Biomedical Sciences, 9308 University of Padova, 3513 Padova, Italy; ⊥ Institute of Atomic Physics and Spectroscopy, Faculty of Science and Technology, University of Latvia, Riga LV-1004, Latvia; # Biomedical Research Centre, Sumy State University, Sumy 40007, Ukraine; ∇ Department of Physiology and Pathophysiology, University of Manitoba, Winnipeg, Manitoba R3E 0J9, Canada; ○ Immunology Center of Georgia, 1421 Augusta University, Augusta, Georgia 30912, United States; ◆ Department of Pharmacology and Toxicology, Augusta University, Augusta, Georgia 30912, United States; ¶ Department of Bioengineering, 6572 University of Pennsylvania, Philadelphia, Pennsylvania 19104, United States; ⋈ Center for Neuroengineering and Therapeutics, University of Pennsylvania, Philadelphia, Pennsylvania 19104, United States; ⧓ Department of Neurology, University of Pennsylvania, Philadelphia, Pennsylvania 19104, United States; ⧖ Institute for Complex Systems CNR Consiglio Nazionale delle Ricerche, 00185 Rome, Italy; ● A.J. Drexel Nanomaterials Institute and Department of Materials Science and Engineering, 6527 Drexel University, Philadelphia, Pennsylvania 19104, United States; ¤ Department of Biomedical Engineering and Biotechnology, Khalifa University of Science and Technology, Abu Dhabi 127788, United Arab Emirates; ☼ Center for Biotechnology, Khalifa University of Science and Technology, Abu Dhabi 127788, United Arab Emirates; ◎ Department of Biomedical Engineering, Faculty of Engineering, Ankara University, 06830 Ankara, Türkiye; ◑ University of Sassari, 07100 Sassari, Italy

**Keywords:** MXenes, two-dimensional materials, bioelectronics, regenerative medicine, cancer nanotherapy, biosensing, immunomodulation, sustainable nanomaterials

## Abstract

MXenes, a rapidly expanding family of two-dimensional transition-metal carbides, nitrides, and carbonitrides, have gained considerable attention as highly versatile nanomaterials for biomedical applications owing to their high electrical conductivity, chemically tunable surfaces, mixed ionic–electronic transport, and controllable redox activity. This review provides a structured overview of MXene-based biomedical technologies, organized by their functional interaction with the human body. We first examine on-body applications, highlighting MXene-enabled wearable, epidermal, and implantable bioelectronics. We then discuss in-body applications, including regenerative medicine, advanced cancer therapies, and antimicrobial strategies, emphasizing how MXene chemistry regulates biological interactions and therapeutic performance. Finally, we address applications outside the body, such as biosensing and diagnostic platforms, where MXenes enhance sensitivity, signal transduction, and imaging capabilities. Beyond summarizing recent advances, this review critically analyzes current gaps in knowledge, including long-term biocompatibility, degradation pathways, immune interactions, and structure–activity relationships, which currently limit clinical translation. We conclude by outlining key future directions, including sustainable synthesis, oxidation-resistant and fluorine-free MXenes, and the integration of artificial intelligence to accelerate materials discovery and application-specific design. Together, these insights position MXenes as chemistry-programmable nanoplatforms with strong potential to bridge on-body, in-body, and outside-the-body biomedical technologies.

## Graphical abstract

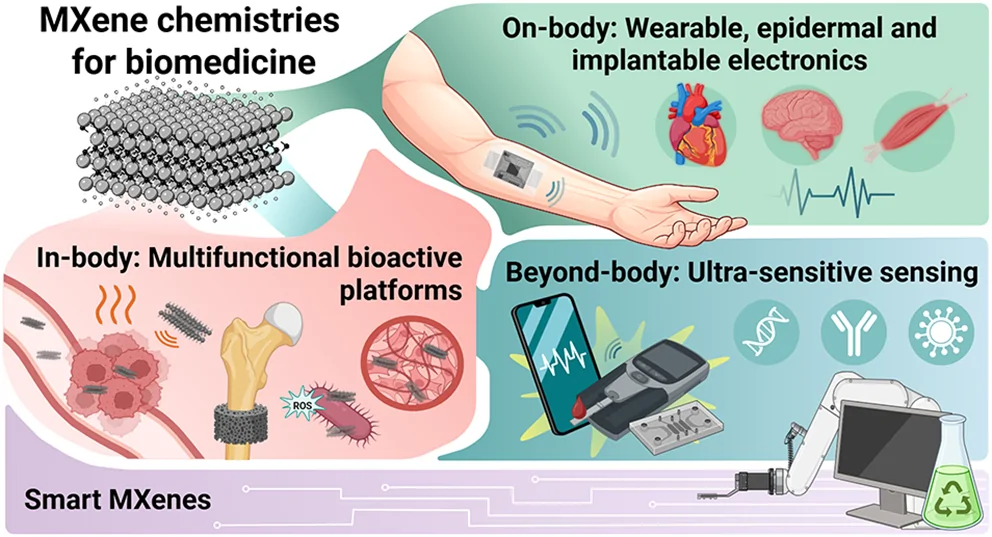

## 1 MXene Versatile Chemistry: One Material Family, Many Journeys

MXenes are a class of two-dimensional (2D) nanomaterials that have attracted significant attention in a wide range of biomedical applications.^1^ MXenes are transition metal carbides, nitrides, and carbonitrides with 2D morphology and a layered structure. The chemistry of MXenes is M_*n*+1_X_*n*_T_*x*_, where M represents an early transition metal (e.g., Ti, V, Mo, Ta, etc.), X represents carbon and/or nitrogen, and T_*x*_ represents surface groups (typically =O, −OH, −F, or −Cl) and *n* = 1 to 4 (Figure 1).^2^ Furthermore, due to their unique structure, MXenes exhibit a range of distinctive and highly tunable physicochemical properties, including interlayer spacing, termination-dependent hydrophobicity/hydrophilicity, high strength, oxidation resistance, low thermal conductivity, and high electrical conductivity. Due to this combination of properties, MXenes are attractive for sensing, imaging, and electrophysiology applications (Figure 1).^3^

**Figure 1:**
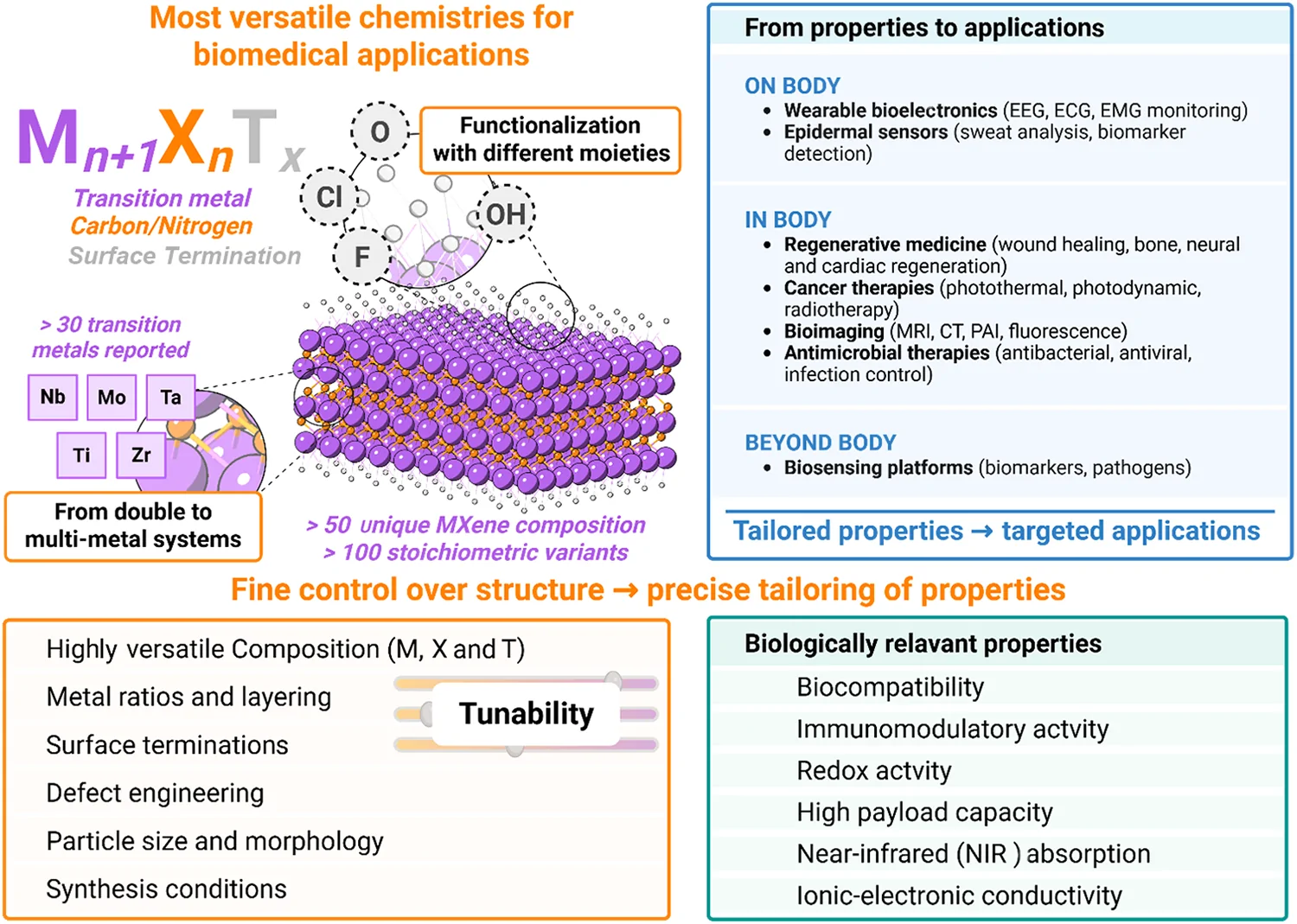
Schematic overview of the chemical versatility and biomedical relevance of MXenes. The M_*n*+1_X*_n_* T_*x*_ structure highlights tunable composition (transition metal, carbon/nitrogen, and surface terminations such as −O, −OH, −F, −Cl), enabling functionalization with diverse moieties and the design of multimetal systems. Control over composition, surface chemistry, defect density, particle size, and synthesis conditions allows precise tuning of physicochemical properties. These features confer key biological functionalities, including biocompatibility, immunomodulation, redox activity, high payload capacity, near-infrared absorption, and mixed ionic–electronic conductivity. Such properties underpin a wide range of applications spanning on-body (wearable bioelectronics and epidermal sensors), in-body (regenerative medicine, cancer therapy, bioimaging, and antimicrobial treatments), and beyond-body (biosensing platforms), illustrating how tailored MXene chemistries enable targeted biomedical applications. (Created with BioRender.com).

MXenes are typically synthesized using a selective chemical etching and delamination technique starting from the MAX phase, which is composed of layered carbide/nitride precursors, where M is the early transition metal, A is Al, Si, or other metals/metalloids, and X is C or N. First, the Group A element is chemically etched away, typically with an acid containing fluoride ions, and replaced with hydroxyl, oxygen, or fluorine surface terminations for MXene formation.^4^ Although hydrofluoric acid (HF)^5^ is toxic, it is a common chemical, and concentrated hydrofluoric acid–based etching is commonly used for the synthesis of many types of MXenes.^6^ After etching, X-ray diffraction (XRD) and scanning electron microscopy (SEM)^7^ can be used to confirm the formation of MXene multilayers, which are then delaminated into single or multiple nanosheets. Delamination is typically induced by incubating the MXene multilayer with organic solvents, such as tetramethylammonium hydroxide (TMAOH) or dimethyl sulfoxide (DMSO), and salts, such as LiCl. The specific solvent and salt may differ based on the metal composition of the MXene and the production method.^8–10^ In recent years, safer and more environmentally friendly etching routes such as lithium fluoride/hydrochloric acid (LiF/HCl) assisted etching, molten salt etching, and electrochemical exfoliation have emerged as alternatives to HF-based synthesis, thereby improving biocompatibility and scalability for biomedical and industrial research.^11,12^ Since their discovery in 2011, over 100 distinct MXene compositions have been successfully synthesized, with hundreds more predicted through computational modeling. The ability to create solid solutions, including high-entropy compositions, and design ordered multimetal architectures gives rise to an almost limitless number of possible variants.^13,14^ This unprecedented chemical versatility positions MXenes as one of the most tunable inorganic material families to date, unlocking extraordinary opportunities for innovation in biomedicine. The chemical tunability of MXenes is primarily governed by synthesis conditions, which determine their surface terminations and influence properties such as hydrophilicity, electronic conductivity, redox activity, and catalytic behavior. Etching methods, ranging from traditional fluoride-based acids to recent fluoride-free strategies, play a central role in shaping surface chemistry. For instance, fluoride-rich environments typically result in −F, −O, and −OH-terminated MXenes, while alternative routes can yield oxygen-rich surfaces that enhance electrical conductivity and electrochemical reactivity (Figure 1).^15^ The chemical tunability of MXenes extends beyond surface terminations. The selection of M and X elements, or the introduction of double-metal or rare-earth elements, allows for tuning of electronic, magnetic, and structural properties. The formation of solid solutions and ordered configurations, such as in-plane or out-of-plane arrangements of different metals, opens new frontiers in controlling properties, including the modulation of metallic, semiconducting, or even topological-insulating behavior.^13,14^ Computational modeling and machine learning have been instrumental in predicting stable configurations, guiding experimental efforts, and accelerating discovery.^13^ By integrating machine learning with large high-throughput density functional theory (DFT) data sets, researchers can now predict stable structures, optimal elemental combinations, and favorable surface terminations, streamlining the discovery and design of next-generation MXenes.^16^ Notably, the advent of high-entropy MXenes, which integrate up to nine transition metals in a single 2D flake, has expanded the structural and chemical landscape, revealing novel electrochemical and magnetic phenomena (Figure 1).^13,14^

The ability to engineer surface terminations enables precise control over properties such as redox activity and charge-transfer resistance, thereby contributing to low detection limits and high selectivity. Additionally, surface terminations influence infrared (IR) reflectivity and optical absorption in a broad wavelength range, with implications for applications in photothermal therapy (PTT), sensing, and electromagnetic interference shielding.^13^

The incorporation of emerging elements, such as phosphorus and antimony, into surface terminations further broadens the chemical scope, enabling fine-tuning of interactions with biological systems.^13^ Importantly, recent innovations in synthesis methods, including the development of oxidation-resistant and fluorine-free materials, are overcoming previous limitations in MXene stability, scalability, and environmental compatibility, paving the way for real-world deployment.

By examining 2,784 potential MXene compositions through high-throughput DFT calculations, 998 were identified as thermodynamically stable or metastable.^17^ The findings demonstrate that both the transition metal core and the surface termination chemistry play fundamental roles in governing structural stability and functional performance. Surface terminations such as −O, −F, −Cl, −Br, −I, −Te, and −Sb were shown to markedly influence electrochemical behavior, catalytic activity, and thermal robustness. In particular, halogen terminations frequently contribute to enhanced thermodynamic stability, underscoring their relevance for advanced applications such as energy storage electrodes. Moreover, numerous promising yet unexplored MXene compositions involving lanthanides and actinides have emerged, greatly extending the compositional landscape beyond the traditionally studied Ti-based systems.^17^

Their 2D layered morphology, combined with a large accessible surface area and tunable interlayer spacing, enables efficient loading, adsorption, and intercalation of small-molecule drugs, biomacromolecules, and growth factors. These characteristics make MXenes particularly attractive platforms for drug delivery, regenerative medicine, and cancer therapy, where controlled loading and release are essential for therapeutic efficacy.^18,19^ Beyond cargo delivery, MXenes possess high electronic conductivity and mixed ionic–electronic transport, a rare combination that supports rapid signal transduction at biological interfaces. This property is central to their successful integration in electrophysiology, bioelectronics, implantable and epidermal electrodes, and cardiac and neural tissue engineering, where synchronized electrical signaling governs tissue function and maturation.^20^ In parallel, MXenes exhibit strong absorption across the near-infrared (NIR)^21^ spectrum and high photothermal conversion efficiency, enabling precise spatiotemporal control over heat generation. This feature has been widely exploited in bioimaging, photothermal, photodynamic, sonodynamic therapies and radiotherapy, as well as in externally triggered antibacterial strategies and on-demand drug release, where localized activation minimizes damage to surrounding healthy tissue.^22^ A defining advantage of MXenes is their rich and highly tunable surface chemistry, which is dictated by surface terminations (−O, −OH, −F, −Cl, etc.) and postsynthetic functionalization strategies. This chemical versatility allows fine control over biocompatibility, immune interactions, targeting specificity, facilitating applications in immunomodulation, infection-resistant implant coatings, and targeted cancer therapies.^23,24^

Importantly, MXenes also display composition- and termination-dependent redox activity, enabling them to either generate or scavenge reactive oxygen species (ROS) depending on their design and mode of activation. This dual capability supports both antimicrobial activity and cytoprotective antioxidant behavior, which is particularly relevant for wound healing, diabetic ulcer treatment, and inflammation control.^25,26^ Finally, the mechanical robustness, flexibility, and processability of MXenes into hydrogels, fibers, and 3D-printed architectures further extend their applicability to regenerative scaffolds, wearable biomedical devices, and soft-tissue interfaces. Together, these interconnected properties illustrate how a single MXene material platform can be rationally engineered to address diverse biomedical challenges, positioning MXenes as programmable and multifunctional nanomaterials (Figure 2).^24,27^ 
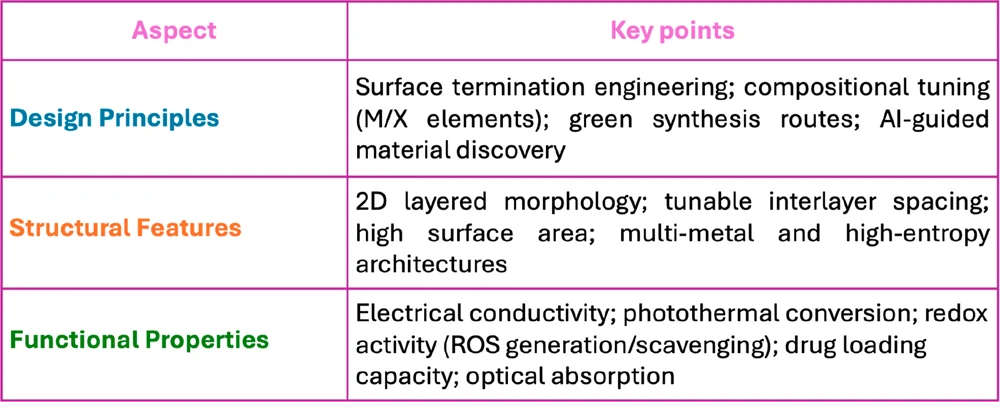


**Figure 2:**
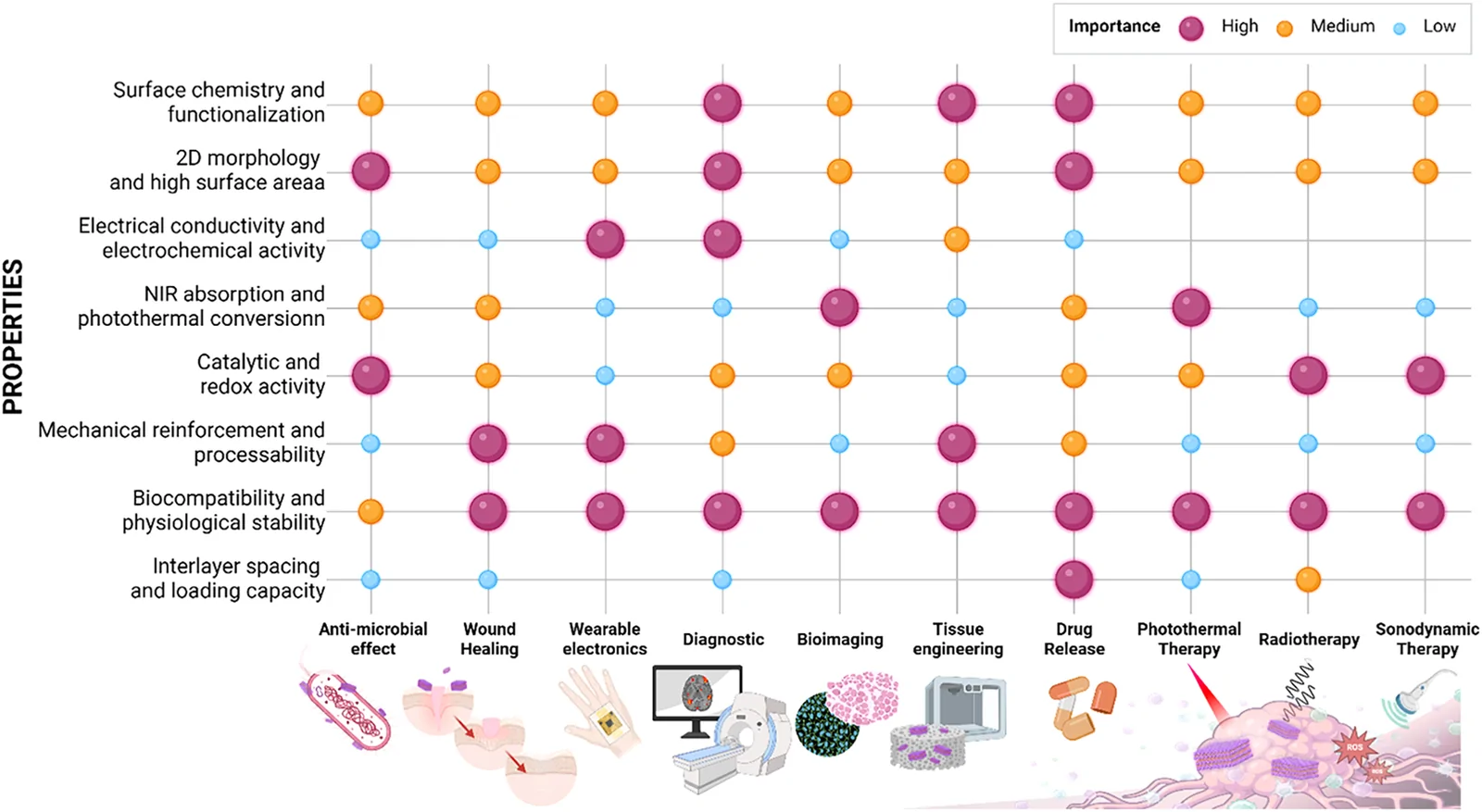
Relative importance of MXene properties across different biomedical and technological applications. The diagram summarizes the relevance of key MXene physicochemical and biological properties for a range of applications, including antimicrobial activity, wound healing, wearable electronics, diagnostics, bioimaging, tissue engineering, drug delivery, PTT, radiotherapy, and sonodynamic therapy. Each circle represents the importance of a given property for a specific application. Circle size reflects relative importance, with larger spheres indicating higher relevance, while color coding denotes importance level (high, medium, or low). This qualitative mapping highlights how distinct MXene properties selectively contribute to application-specific functional requirements and provides a comparative framework to guide material design and application-driven optimization. (Created with BioRender.com).

## 2 Biocompatibility of MXenes: a Chemistry-Driven Perspective

MXenes are widely reported as biocompatible 2D nanomaterials when their chemistry, purity, and dose are properly controlled, with numerous studies demonstrating minimal cytotoxicity and limited immune activation *in vitro* and *in vivo*.^18,28^ Scheibe et al. reported that Ti_3_C_2_ MXenes displayed lower cytotoxicity toward normal human fibroblasts than toward cancer cells.^29^ *In vivo* histological and hematological evaluation confirmed the superior biocompatibility of MXenes. Their favorable biological profile arises from intrinsic hydrophilicity, metallic conductivity, and chemically addressable surface terminations.^24^ Importantly, MXenes preserve cell viability, mitochondrial function, and immune homeostasis at low-to-moderate concentrations, whereas higher doses can overwhelm cellular antioxidant defenses, leading to oxidative stress and inflammatory signaling.^30^ When MXenes exhibit reduced biocompatibility, the causes are predominantly chemical rather than purely physical. Excessive fluorine-rich terminations (−F), residual etching byproducts, or incomplete washing promote membrane destabilization and ROS generation, while uncontrolled oxidation at edges and defect-rich basal planes accelerates degradation into metal oxides (e.g., TiO_2_), which can stimulate pro-inflammatory cytokines such as interleukin-6 (IL-6) and interleukin-8 (IL-8).^22,26^ Furthermore, specific MXene compositions and oxidation states can cause ion release and redox activity, inducing macrophage activation, inflammasome engagement, and immune polarization.^24^ In contrast, MXenes enriched in −O/–OH terminations, produced via mild or fluoride-free etching and stabilized through surface passivation, show markedly reduced ROS production, attenuated inflammatory responses, and improved tissue compatibility, even under prolonged exposure.^23,25,31,32^ Collectively, these studies establish that MXene biocompatibility is not an intrinsic material constant, but a chemistry-driven and dose-regulated property, underscoring the importance of termination engineering, oxidation control, and immune-aware “safe-by-design” strategies for biomedical translation. Nevertheless, critical gaps remain regarding the long-term fate of MXenes in biological systems, including their chronic biodistribution, biodegradation pathways, and clearance mechanisms, as well as the interaction between surface chemistry and immune modulation under physiologically relevant exposure scenarios. Moreover, comprehensive structure–activity relationships linking MXene composition, defect density, and termination heterogeneity to cell-type–specific biological responses and *in vivo* outcomes have yet to be fully established, limiting predictive toxicological assessment and evidence-based regulatory translation.
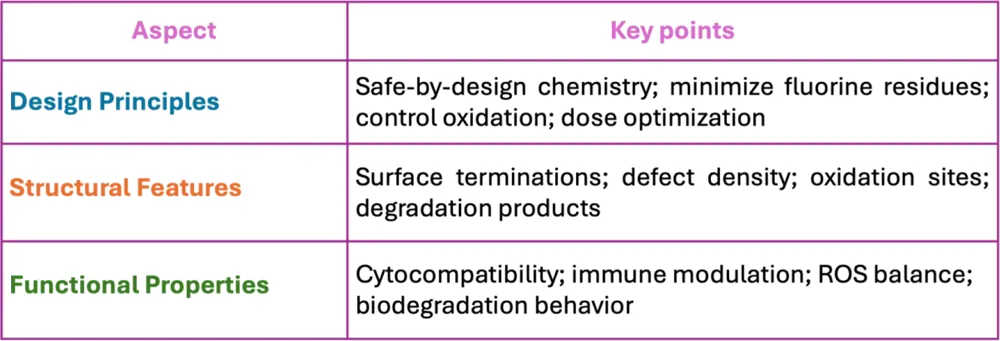


## 3 MXenes for On-Body Biointerfaces

### 3.1 Wearable, Epidermal, and Implantable Electronics

MXenes have rapidly emerged as powerful materials for electrophysiology owing to their unique combination of metallic conductivity, 2D morphology, and tissue-compatible surface chemistry. In particular, Ti_3_C_2_T*_x_* MXenes exhibit electrical conductivities comparable to those of metals while remaining mechanically flexible and hydrophilic, enabling intimate, low-impedance coupling with soft biological tissues.^14,20^ These properties have been exploited in neural and cardiac electrophysiology, where MXene-based electrodes outperform conventional gold or poly­(3,4-ethylenedioxythiophene):polystyrenesulfonate (PEDOT:PSS) interfaces by reducing interfacial impedance and improving long-term recording stability (Figure 3a).^20,33^ Moreover, the mixed ionic–electronic conductivity of MXenes supports efficient transduction of bioelectric signals while minimizing polarization losses at the electrode–tissue interface, a critical requirement for high-fidelity stimulation and recording.^27^ MXenes further enables integration into flexible, stretchable, and epidermal electrophysiological devices, facilitating applications from surface electromyography (EMG), electroencephalography (EEG)^34^ and electrocardiography (ECG) to implantable neural interfaces and osteosarcoma treatment (Figure 3b).^24,33,35^ Collectively, these studies establish MXenes as a new class of electrophysiological materials that bridge the gap between rigid metallic electrodes and soft biological systems, opening pathways toward next-generation bioelectronic and neuromodulation technologies. The intercalated layers of MXenes, when subjected to mechanical vibrations, produce colloidal solutions of single to few-layer MXenes. These can be easily processed into free-standing flexible films through vacuum-assisted filtration while maintaining metallic conductivity. Such films can be patterned into user-defined shapes and sizes, with their thickness and conductivity tailored by adjusting the solution concentration and flake size.^36^ MXenes form stable, water-based conductive inks without the need for surfactants or additives, enabling the easy fabrication of thin films via printing, coating, or casting.^36^ The mechanical properties of MXenes are influenced by the strong interatomic bonds and the number of atomic layers in the structure, providing the flexibility, bendability, and high fracture strength to MXene films and coatings.^37^ Computational studies, such as those by Borysiuk et al., highlight the exceptional bending ability of MXene nanoribbons, suitable for on-body applications.^38^ Vitale and colleagues were the first to introduce MXene electrodes for wearable electrophysiology recording (also named “MXtrodes”), without the need for conductive gels and with electrochemical properties exceeding those of conventional bioelectronic materials (e.g., Pt, Au).^20^ The scalable fabrication enabled by the liquid-phase processability of Ti_3_C_2_T_*x*_ MXene allowed full customization of the device geometry, scale, and channel density for applications ranging from high-density muscle activity mapping to noninvasive brain monitoring.^20^ In the area of implantable neural interfaces, transparent MXene microelectrode arrays enable multimodal neural sensing via electrophysiological recording and optical imaging, by combining optical transparency with low impedance, another advantage over conventional electrode materials (Figure 3c).^33^ Beyond dry bioelectronics, MXene-based hydrogels have emerged as promising materials for soft, skin-conformable electronics. These systems address longstanding limitations in hydrogel electronics, including poor mechanical strength, dehydration sensitivity, and low sensing responsiveness, toward dynamic wearable sensing platforms.^39^ The study of Cui Ye et al. demonstrated that MXene-based epidermal sensors enable ultrasensitive, noninvasive, and real-time molecular monitoring directly at the skin interface, combining high electrical conductivity, mechanical flexibility, and skin conformability. By integrating AuNP–MXene electrodes into a wearable patch, the work achieves subpicomolar detection of oestradiol in sweat and wireless data transmission, validating reliable on-body performance in human subjects. This establishes MXenes as key enablers for next-generation epidermal sensors, capable of translating trace-level biochemical signals from sweat into clinically relevant, personalized health information (Figure 3d).^7,33^ MXenes can also be integrated into textile-based electronics such as printed wireless charging coils, textile supercapacitors, and fully integrated on-garment energy grids. Such multifunctional e-textile systems enable the recording of digital biomarkers, including real-time electromyography, thermoregulation via Joule heating, and energy harvesting (Figure 3e).^33,39^

**Figure 3:**
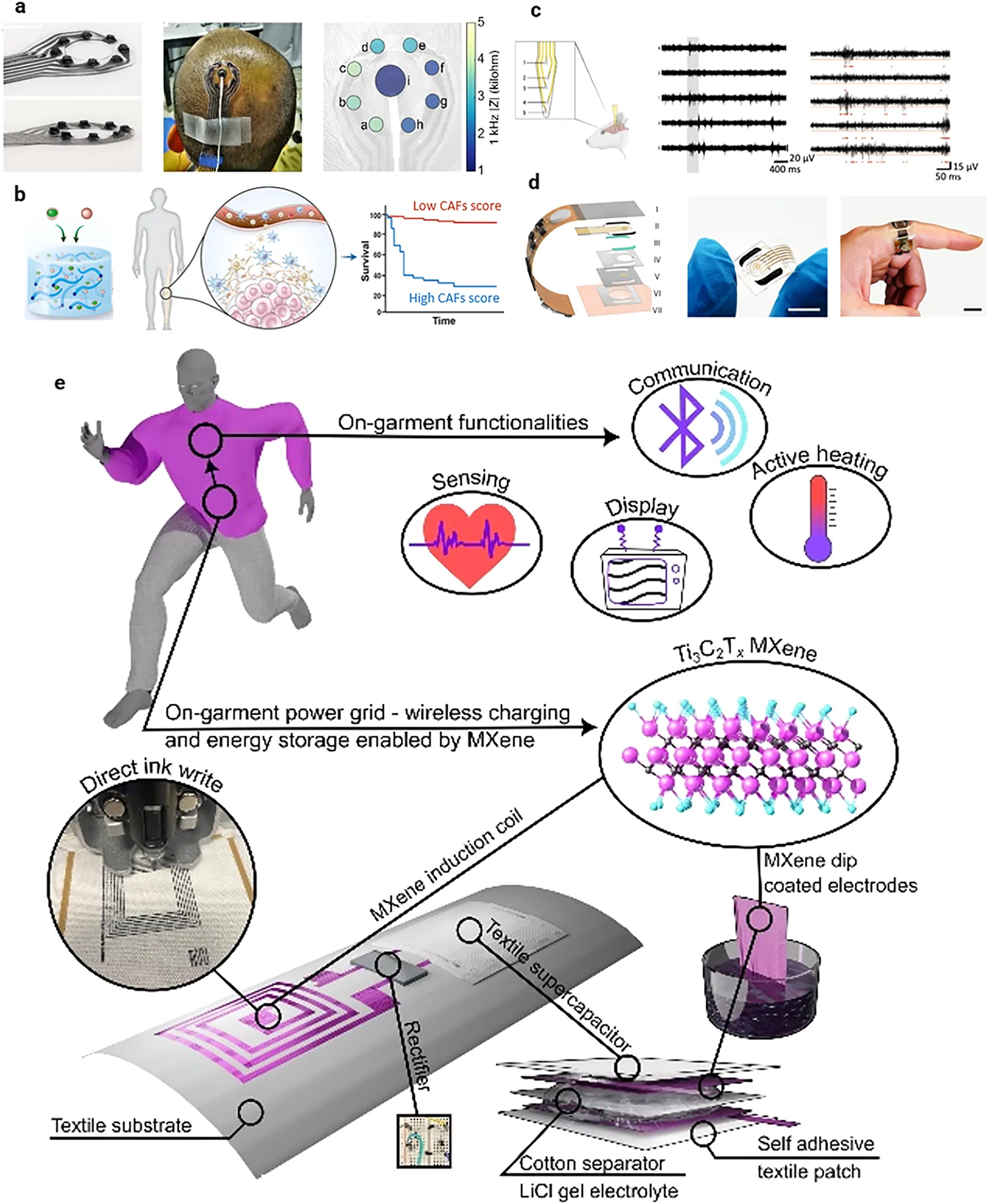
MXene-enabled biointerfaces and wearable systems for sensing, therapy, neural recording, and integrated power. (a) Dry EEG recording enabled by 3D pillar MXtrodes. Photographs of a MXtrode-based 3D EEG array with MXene electrodes, comparison with standard gelled Ag/AgCl electrodes on a human subject, and a map of electrode impedance values. Reprinted with permission from Driscoll et al., 2021.^20^ Copyright 2021 American Association for the Advancement of Science. (b) Hydrogel-enabled sonodynamic therapy for cancer treatment. A localized delivery platform combining a nanosonosensitizer and a SMAD3 inhibitor enhances ROS generation, remodels the tumor extracellular matrix, and promotes immune cell infiltration, leading to strong antitumor efficacy *in vivo*. Reprinted with permission from Lin et al., 2026.^35^ Copyright 2025 Elsevier B.V. (c) Intracortical recording using transparent electrode arrays. Electrode placement, representative neural recordings, and spike detection demonstrating high-quality neural signal acquisition. Scale bars represent 400 and 50 ms (ms). Reprinted with permission from Shankar et al., 2025.^33^ Copyright 2024 Wiley-VCH GmbH. (d) Flexible and wireless microfluidic wearable patch for sweat analysis. Schematic of the device architecture and layered design, along with photographs of a disposable sensor patch and a fully integrated wearable system for hormone monitoring. Reprinted with permission from Ye et al., 2024.^7^ Copyright 2024 Springer Nature BV. (e) MXene-enabled wearable power solutions. Schematics illustrating the need for on-garment power and an integrated approach combining MXene-based wireless charging, conductive traces, bonding lines, and a textile-based MXene supercapacitor. Reprinted with permission from Inman et al., 2024.^39^ Copyright 2024 Elsevier B.V..

In the study by Xiaoqi Yin et al., MXene-based composites have been developed using chewed gum as a flexible matrix, toward sustainable and unconventional platforms for wearables. Through iterative stretching and kneading, MXene nanosheets can be uniformly integrated into the gum, yielding a conductive, adhesive, and self-healing material. In addition to serving as a flexible motion sensor, the composite leverages the photothermal properties of MXenes to function as a thermoelectric generator under solar illumination, offering a unique route for energy harvesting and waste upcycling.^40^ Despite rapid progress in MXene-enabled wearable, epidermal, and implantable electronics, a critical gap remains in the predictive understanding of long-term material–device–tissue interactions under realistic operating conditions. In particular, how MXene composition, flake size, defect density, and surface terminations influence electrical stability, mechanical fatigue, interfacial adhesion, and bioelectronic signal fidelity during prolonged skin contact or implantation remains poorly defined. Moreover, most demonstrations focus on short-term or proof-of-concept performance, leaving unresolved questions regarding chronic biostability, oxidation-driven property drift, immune responses, and degradation pathways in epidermal and implantable settings.
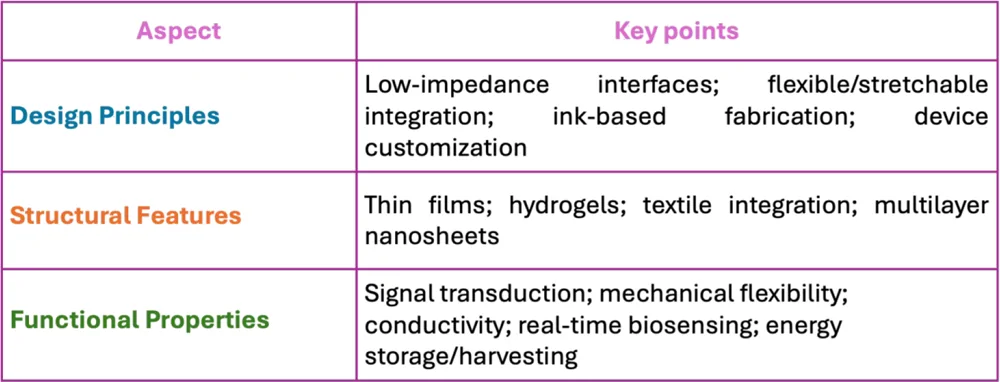


## 4 MXenes as Multifunctional Platforms for In-Body Applications

### 4.1 MXenes in Regenerative Medicine

The unique features of MXenes, including their biocompatibility, immunomodulatory activity and electrical conductivity, make them promising candidates for use in regenerative medicine.^27,41–43^ The regenerative effects of MXenes include bacterial clearance from infected wounds and accelerated healing of damaged tissue (skin and bone) via stimulation of related signaling pathways, including survival and angiogenesis. They also alleviate oxidative stress through downregulation of ROS, promote neuronal and cardiac differentiation via electronic stimulation for tissue repair. Their effects are not only limited to regeneration but also include drug delivery to the target site.^44^ While this section primarily focuses on wound healing, bone regeneration, and neuronal differentiation in regenerative medicine, recent studies have also begun to investigate their potential for cardiac tissue repair, which is also discussed here.

The main mechanisms of action of MXenes in regenerative medicine include antimicrobial activity via ROS-mediated oxidative stress to membrane disruption, regulation of excessive ROS levels in wound healing, and stimulation of cell survival and proliferative signaling pathways.^45,46^ MXenes can induce ROS generation at the bacterial membrane, thereby disrupting it, as discussed in the antimicrobial section. While MXenes can induce ROS production as antimicrobial agents, they scavenge ROS in regeneration mainly through their surface chemistry and electron-transfer capability. The surface of MXenes terminated with functional groups such as −OH, −O, and −F, and the transition metal core (e.g., Ti in Ti_3_C_2_T_*x*_) can donate electrons to neutralize ROS like superoxide (O_2_^–•^) and hydroxyl radicals (^•^OH), converting them into H_2_O or O_2_, etc., to stimulate tissue regeneration.^47^

In regenerative medicine applications, hydrogels are widely used to promote healthy wound healing,^48,49^ and the inclusion of MXenes into three-dimensional (3D) structured hydrogels has the potential to increase the efficacy of hydrogel-based treatments.^50^ MXene-based hydrogels can support healthy wound regeneration through two major mechanisms: first, the antibacterial properties of MXenes may help prevent the establishment of infection at the wound site, and second, electrical stimulation of the MXene can enhance cell proliferation and the development of cell-to-cell communication networks (Figure 4ia).^51^ For example, the addition of Ti_3_C_2_T_*x*_ MXene to a reduced graphene oxide hydrogel significantly increased cellular interactions in a cell-type-independent manner, indicating that MXenes promote cellular network formation.^52^ Likewise, a polycaprolactone-Ti_3_C_2_T_*x*_ composite scaffold provided a favorable environment for cellular attachment and growth while maintaining antibacterial activity and electrical conductivity over a wide temperature range.^53^ Furthermore, Ti_3_C_2_T_*x*_ promotes skeletal muscle regeneration, myoblast differentiation, and muscle fiber formation. It also reduces inflammation via anti-inflammatory M2 macrophage polarization through the transformation of inflammatory M1 and decreases oxidative stress by acting as an antioxidant.^54^

**Figure 4:**
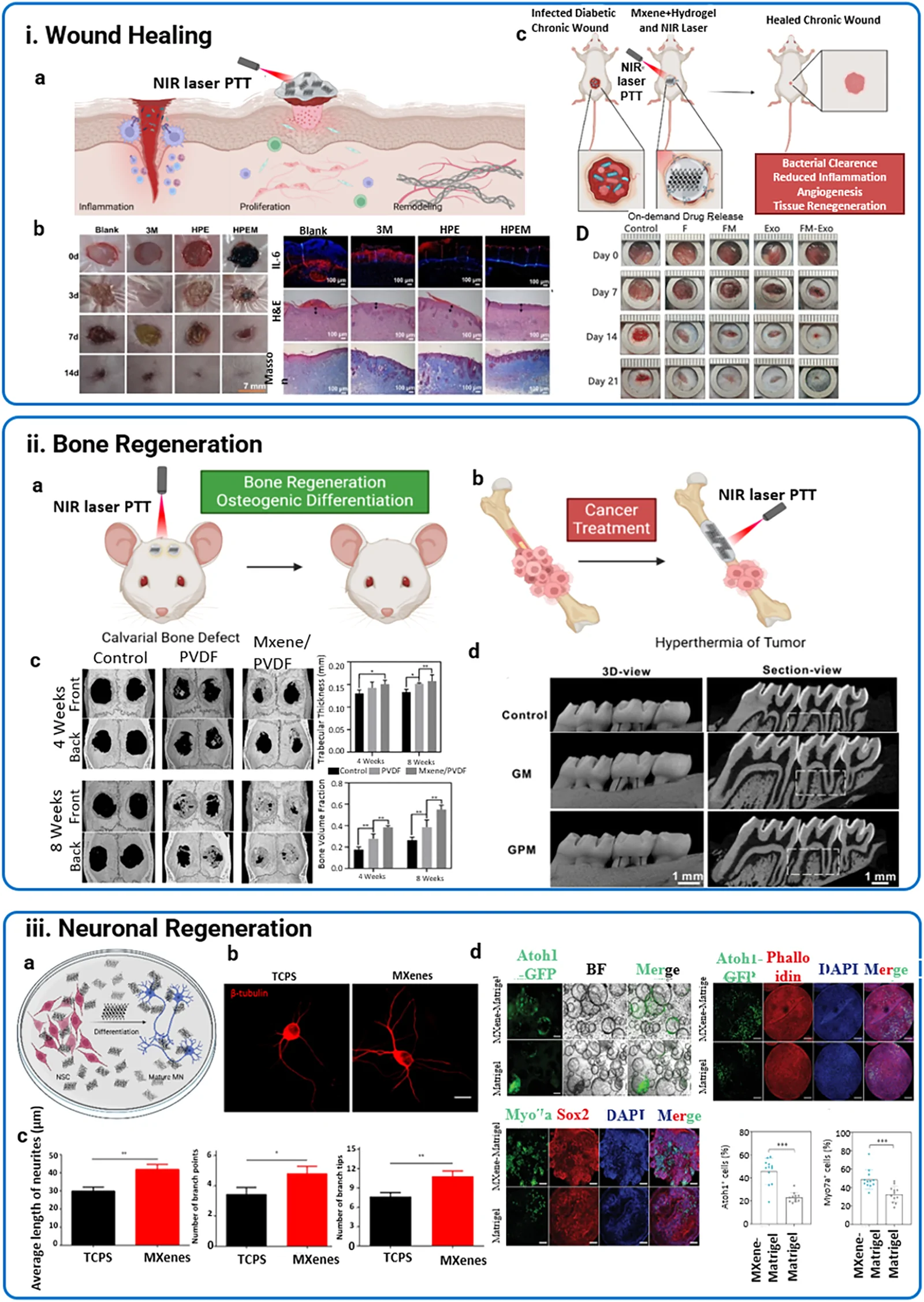
MXenes in Regenerative Medicine. **i**. **Wound healing**. (a) Schematic representation of wound healing and the application of MXene composite to reduce inflammation due to the upregulation of Tregs and anti-inflammatory M2 macrophage polarization (Created with BioRender.com). (b) *In vivo* MRSA-infected wound healing with MXene-containing composite. IL-6 immunofluorescence, H&E, and Masson’s trichrome staining images are presented for the evaluation of inflammation reduction and tissue healing. Reprinted with permission from Zhou et al., 2021.^55^ Copyright 2021 American Chemical Society. (c) Schematic representation of applying MXene and hydrogel composite with NIR treatment in infected diabetic chronic wounds to increase tissue regeneration (Created with BioRender.com). (d) Wound healing images in diabetic mice. Reprinted with permission from Jiang et al., 2024.^56^ Copyright 2024 American Chemical Society. **ii**. **Bone regeneration**. (a) The dual effect of MXene application in bone. The application of MXene hydrogel composite with NIR laser exposure induces osteogenic differentiation, and biodegradation helps the mineralization of the bone for regeneration in calvarial bone defected rats (Created with BioRender.com). (b) MXene hydrogel composite with NIR laser exposure causes hyperthermia in the cancer cells and induces cancer cell death. (c) MXene PVDF composite applied 3D reconstruction images of the rat cranial defect model, showcasing both front and back views. Reprinted with permission from Fu et al., 2023.^57^ Copyright 2023 American Chemical Society. (d) The impact of injectable hydrogels on in situ periodontal bone regeneration was evaluated. Maxillary second molars (M2) were scanned using a micro-CT device following treatment with an injectable hydrogel containing gelatin, Ti_3_C_2_T_*x*_ MXene nanosheets, and poly l-lysine. Reprinted with permission from Yu et al., 2024.^58^ Copyright 2024 American Chemical Society **iii**. **Neuronal Regeneration**. (a) MXene treatment induced Neuron Differentiation (Created with BioRender.com). (b) Ti_3_C_2_T_*x*_ MXene induced neuron growth and development. (c) Ti_3_C_2_T_*x*_ MXene induced neuron growth evaluated in matters of length of neurites, number of branch points and branch tip. (b, c) Reprinted with permission from Li et al. 2022.^59^ Copyright 2022 Springer Nature. (d) Confocal images of cochlear organoids with hair cell markers, including Atoh1 and Myo7a and percentages of hair cells with the Ti_3_C_2_T_*x*_ MXene incorporated Matrigel. Reprinted with permission from Zhang et al., 2022.^60^ Copyright 2022 Wiley-VCH GmbH.

#### 4.1.1 Wound Healing

The skin is the largest organ, and any injury to the skin triggers a natural wound-healing process composed of four stages: hemostasis, inflammation, proliferation, and remodeling (Figure 4ia).^61^ The body initiates hemostasis immediately upon injury. After hemostasis is achieved, innate immune cells, including macrophages and neutrophils, are recruited to the site of injury to alleviate inflammation and pathogen clearance. During the proliferative phase, the body begins several processes, including neovascularization, extracellular matrix (ECM) formation, re-epithelization, and granulation, tissue formation to replace lost or dead cells and restore skin integrity. Finally, in the remodeling phase, scar tissue is replaced with newly produced skin cells, and skin integrity is fully regained; previous hemostatic, inflammatory, or proliferative activity ends.^62^ The overall rate and efficacy of this four-stage wound-healing process can be influenced not only by endogenous conditions such as aging, hormone levels, nutrition, oxidative imbalance, poor vascularization and chronic diseases (e.g., diabetes) but also by exogenous factors such as bacterial infections, hypoxia, additional stress or smoking.^61,63^

##### 4.1.1.1 Cell Proliferation and Cytoprotection

Several studies have demonstrated the efficacy of MXene-based therapeutics, including hydrogels, for promoting more rapid wound healing and skin regeneration. In one study, poly­(vinyl alcohol)-based hydrogel dressings were enriched with Ti_3_C_2_T_*x*_ and polyaniline to promote wound healing through several mechanisms, including the downregulation of proinflammatory cytokines, antibacterial activity against *Escherichia coli* and *Staphylococcus aureus* under NIR stimulation, upregulation of vascular endothelial growth factor (VEGF) and α-smooth muscle actin (α-SMA), as well as enhanced angiogenesis and collagen deposition.^64^ In a separate study, Yu et al. (2023) developed 2D CaO_2_-loaded titanium carbide nanosheets with sonodynamic properties. These features arise from the *in situ* oxidation of Ti_3_C_2_T_*x*_ by CaO_2_, which generates the acoustic sensitizer, TiO_2_, on the material’s surface. At the same time, the hemodynamic features of these nanosheets promote the Fenton reaction, which involves the generation of hydroxyl radicals through the interaction of ferrous iron (Fe^2+^) with H_2_O_2_. The nanosheets were applied to MRSA-infected wounds in C57BL/6JNifdc mice and then activated with ultrasound. In mice treated with the CaO_2_-loaded Ti_3_C_2_T_*x*_, wounds exhibited an increased presence of CD31-expressing vascular endothelial cells, indicative of angiogenesis, along with reduced inflammation and substantial collagen deposition.^65^ Li et al. (2022) similarly designed a chitin/MXene-based composite sponge containing gold nanoparticles to induce wound healing by reducing blood loss, accelerating skin cell migration to the wound site, and providing antibacterial and photothermal activity. Their product successfully resulted in hair follicle and collagen formation.^66^ Likewise, an MXene-NH_2_/chitosan/copper hemostatic sponge with antibacterial activity has been shown to stimulate wound healing.^67^ Zhou et al. (2021) tested a multifunctional scaffold composed of Ti_3_C_2_T_*x*_ MXene@PDA, poly­(glycerol-ethylenimine), and oxidized hyaluronic acid *in vivo* in full-thickness methicillin-resistant *Staphylococcus aureus* (MRSA) infected wounds. The scaffold accelerated wound healing by inhibiting bacterial growth and stimulating tissue granulation, thereby reducing inflammation (Figure 4ib).^55^ Elsewhere, it has been reported that V_2_CT_*x*_ MXene effectively mimicked six natural enzymes for ROS scavenging and cytoprotection against oxidative stress.^25^ More recently, Geng et al. (2024) used a V_2_CT_*x*_-containing heterojunction activated by sonodynamic therapy (SDT) to promote wound healing through ROS scavenging and bacterial clearance.^46^ Hu et al. (2024) likewise designed a cost-effective, hydrogel-based wound-dressing composite by cross-linking sodium alginate, agar, Ti_3_C_2_T_*x*_ MXene, and zinc ions (Zn^2+^) for PTT and wound healing applications.^68^ Finally, a MXene@polydopamine-cryptotanshinone (MXene@PDA-CPT) composite showed excellent antibacterial activity,^69^ while a MXene@PDA-decorated nonwoven chitosan fabric has been used as an antibacterial, hemostatic dressing to promote clotting, fibrinogen formation, and scar tissue assembly. The composite fabric specifically promotes tissue granulation and upregulates the collagen production gene COL3A1.^70^

MXene-based wound healing may also be accelerated by exogenous electrical stimulation, as an endogenous direct current electric field arises during the natural wound healing process due to the transepithelial potential difference. This electric field promotes cell adhesion, proliferation, migration, and differentiation.^71^ To test the potential of MXenes for electrically stimulated wound healing, Mao et al. (2020) created a composite hydrogel of Ti_3_C_2_T_*x*_ and regenerated bacterial cellulose, which is desirable in wound healing products because of its high water uptake capacity and biocompatibility. This composite was then applied to full-thickness defects in Sprague–Dawley rats. Rats that received the MXene treatment exhibited higher levels of VEGF, transforming growth factor-β, and epidermal growth factor, which promote re-epithelization, as well as increased CD31-positive microvessel formation, indicative of angiogenesis. These wound healing effects were further magnified, when the hydrogel treatment was coupled with electrical stimulation.^72^

##### 4.1.1.2 Therapeutic Loading and Drug Delivery

MXene-based materials can also be loaded with therapeutic agents to facilitate precise, on-site drug delivery to target tissues. When exposed to NIR light, MXene-based materials will release any loaded pharmaceuticals or compounds into adjacent tissue. For example, Wang et al. (2021) preloaded hepatic growth factor into an MXene-hydrogel composite to trigger c-Met signaling upon photothermal stimulation for survival. On-demand release of hepatic growth factor was then observed *in vivo* in 10 mm-diameter cutaneous wounds on Kunming male mice. The authors monitored the expression of *K*_i_-67 (an indicator of cell proliferation), VEGF-A, and CD31 and observed that NIR light exposure significantly enhanced wound closure and angiogenesis.^73^ Similarly, MXene hydrogels preloaded with tumor necrosis factor-α (TNF-α) can be used to promote apoptosis as part of cancer spheroid treatment. In another study, a Ti_3_C_2_T_*x*_/MoS_2_ composite activated with PTT exhibited antibacterial activity by releasing Mo^4+^ ions, which can invade ruptured bacterial cell membranes to trigger intracellular ROS formation and deplete the antioxidant glutathione. Loading this composite with fibroblast growth factor 21 further promoted cell migration, wound healing, and bacterial clearance *in vivo*.^74^ More recently, when VEGF was incorporated into an MXene-based hydrogel and exposed to NIR light, it boosted skin flap survival by improving angiogenesis, reducing inflammation, and promoting cell migration and proliferation.^75^ Finally, Lu et al. (2023) used the intestinal wrinkles and villi structure to design MXene-based microneedles for accelerated wound healing. They encapsulated human epidermal growth factor in the microneedles, and the needle-like structure allowed for controlled drug release. Under NIR light exposure, the needles achieved 100% wound closure *in vivo* with full skin recovery, skin appendages for hair growth, and excellent remodeling.^76^

##### 4.1.1.3 Diabetic Wound Regeneration

Patients with diabetes are at risk of several additional complications in the wound healing process that may be mitigated by MXene-based treatment. First, diabetic patients exhibit reduced antioxidant production, disrupted hemostasis, and increased ROS levels and oxidative stress, which can induce apoptosis and downregulate proliferation in ways that delay the healing process and increase the chance of infection.^77^ Wei et al. (2022) accordingly tested a sponge-like, macroporous hydrogel containing poly­(acrylic acid)–0.16% Ti_3_C_2_T_*x*_ MXene for wound healing in diabetic mice. After 7 days, wounds in the MXene-containing hydrogel treated group were significantly smaller than those treated with an MXene-free hydrogel, and, unlike the other treatments, hair growth was observed. This accelerated healing in MXene-containing hydrogel-treated mice was supported by increased expression of VEGF and transforming growth factor β (indicating angiogenesis and re-epithelization) and decreased CD68 expression (indicating reduced inflammation) (Figure 4ic).^78^

Second, diabetes can lead to the development of deep ulcers in the lower extremities, which are associated with chronic pain. Yang et al. (2022) encapsulated Fe_3_O_4_@SiO_2_ magnetic nanoparticles within MXene hydrogels and then infused the resulting hydrogel with silver nanoparticles (AgNPs). Upon exposure to NIR lasers, the AgNPs were released as an antibiotic.^79^ The authors then used their AgNP-MXene-based hydrogel system to treat deep wound infections in a diabetic Sprague–Dawley rat model. Mice in the AgNP-MXene-treated group exhibited increased CD31 and α-SMA levels, indicating tissue regeneration and angiogenesis, and reduced IL-6 and TNF-α expression, indicating reduced inflammatory activity. Anti-inflammatory M2 cells also increased with treatment, providing further evidence of reduced inflammatory activity.^79^ In a separate study, Jiang et al. (2024) coupled an F127-MXene hydrogel with M2 macrophage-derived exosomes, small extracellular vesicles that mediate cellular communication and can promote wound healing. This hydrogel exosome composite enhanced wound closure in high-glucose microenvironments both *in vitro* and *in vivo* by activating the PI3K-Akt pathway and regulating inflammation (Figure 4id).^56^ MXenes are valuable agents with dual capabilities for promoting wound healing: first, their antibacterial properties make them a promising option for targeting drug-resistant bacteria and reducing the spread of antimicrobial resistance; second, they facilitate vital regenerative processes such as angiogenesis and tissue granulation. MXenes have accordingly been proven to be highly effective in treating infected chronic or diabetic wounds.

#### 4.1.2 Bone Regeneration

Bone regeneration is a complex, regulated process that occurs naturally during fracture repair and throughout life. However, large-scale trauma or infection, and cancers or cancer treatments such as osteosarcomas or tumor resection, often require additional support for bone regeneration.^80^ MXenes can be used to support bone regeneration through two distinct mechanisms: first, upon exposure to NIR lasers in photothermal applications, MXene-based materials can kill osteosarcoma cancer cells, and second, MXenes can promote osteogenic differentiation and reduce inflammation through the modulation of macrophages (Figure 4iia, iib).^81–83^ MXene-containing composites are therefore widely used as scaffolds for bone remodeling.

##### 4.1.2.1 Osteogenesis and Stem Cell Differentiation

As an example of MXene-based bone regeneration, Zhang et al. (2019) subcutaneously implanted Ti_3_C_2_T_*x*_ MXene sheets in Sprague–Dawley rats to evaluate their biocompatibility and assess their efficacy in treating critical-sized calvarial defects. They found that the MXene sheets promoted earlier osteogenesis and, therefore, more rapid bone regeneration.^84^ Likewise, Ti_3_C_2_T_*x*_–poly­(*N*-vinylpyrrolidone) nanosheets irradiated with NIR induce osteogenic differentiation of bone marrow-derived mesenchymal stem cells (BMMSCs).^85^ Mi et al. (2022) fabricated a composite containing hydroxyapatite (the main ingredient nanophase of the bone), sodium alginate, and Ti_3_C_2_T_*x*_-MXene to treat calvarial bone defects in rat models. Their composite improved bone regeneration and osteogenic differentiation of BMMSCs.^86^

Hu et al. (2023) designed an electroactive hydrogel composed of regenerated silk nanofibril (RSF) and bioencapsulated MXene to promote osteogenic differentiation. This MXene/RSF hydrogel was then implanted *in vivo* in rats with cranial defects, and electrical stimulation was applied every other day. Treatment with the MXene/RSF hydrogel promoted the osteogenic differentiation of BMMSCs and the polarization of CD206^+^F4/80^+^ M2 macrophages, which, in turn, promoted bone regeneration, neoangiogenesis, and endotheliocyte migration. The authors additionally visualized the osteogenic markers RUNX-2 and Col-1 using immunofluorescence staining and demonstrated osteogenic differentiation using alkaline phosphatase and Alizarin Red staining. Finally, they used immunofluorescence staining for α-SMA/CD31 to observe newly formed blood vessels in the MXene/RSF treatment group. RNA sequencing analysis suggested that their MXene/RSF hydrogel primarily promoted osteogenesis by activating the Ca^2+^/CALM signaling pathway.^87^ In this pathway, Ca^2+^ binds to the calmodulin (CALM) complex, inducing a conformational change that activates calmodulin kinase II and calcineurin. Calcineurin dephosphorylates the nuclear factor of activated T cells (NFAT) transcription factor, which allows NFAT to translocate to the nucleus, where it upregulates genes for osteogenic differentiation and proliferation. Wu et al. (2023) designed an NIR photoactivated bone scaffold composed of alginate methacrylate, alginate-*graft*-dopamine, and PDA-functionalized Ti_3_C_2_T_*x*_ MXene (MXene@PDA) to promote bone regeneration through immunomodulation and osteogenesis. In both *in vitro* and *in vivo* calvarial defect models, treatment with the composite decreased the abundance of M1 macrophages carrying the inflammatory markers TNF-α and iNOS, while increasing the abundance of M2 macrophages carrying the anti-inflammatory markers IL-10 and CD206. These findings suggest that the composite effectively scavenged excess ROS, which are often responsible for inducing severe inflammatory reactions at bone injury sites. MXene-treated rats also exhibited significant pro-angiogenic markers and recruitment of stem cells.^88^

Composites containing Nb_2_CT_*x*_ MXene have also been reported to be promising candidates for both bone regeneration and tumor treatment (Figure 4iia,iib).^83^ For example, Yin et al. (2021) integrated ultrathin niobium carbide (Nb_2_CT_*x*_) MXene nanosheets into 3D-printed bone mimetic scaffolds for osteosarcoma treatment and bone regeneration. Under higher-wavelength NIR irradiation (i.e., NIR-II), the MXene-containing scaffold achieved deeper tissue penetration and hyperthermia than non-MXene-based bone mimetic scaffolds, ultimately prolonging the lifespan of BALB/c mice with osteosarcoma. The inclusion of MXene in the scaffold was not only an effective treatment for osteosarcoma but also enhanced bone regeneration, increased osteogenic differentiation of BMMSCs, and increased angiogenesis. Furthermore, *in vivo* degradation of the MXene-based bone mimetic scaffold releases calcium and phosphate, which can promote bone mineralization.^89^ In addition to these promising effects of Nb_2_CT*_x_*, a separate study found that a Mo_2_Ti_2_C_3_T_*x*_ MXene-infused hydrogel promoted both osteogenic differentiation and neurogenesis.^90^

Finally, Fu et al. (2023) prepared a MXene/polyvinylidene fluoride (MXene/PVDF) ferroelectric nanocomposite that could be assembled into a natural ECM in bone tissue to improve regeneration. The MXene-PVDF membranes were then placed into 5 mm-sized bone defects on the cranial raphe in Sprague–Dawley rats. After 8 weeks, the hollow in the cranial raphe had almost closed with immature lamellar bone, vascular channels, and bone lacunae, indicating osteogenesis and mineralization of the ECM (Figure 4iic).^57^

##### 4.1.2.2 Periodontal Applications

In severe periodontitis, periodontal regeneration must be exogenously stimulated. Cui et al. (2021) found that the Ti_3_C_2_T_*x*_ MXene increased the proliferation and osteogenic differentiation of human periodontal ligament cells (hPDLCs) by activating the canonical Wnt/β-catenin pathway, which is responsible for embryonic development, tissue homeostasis and cell proliferation. For example, β-catenin upregulates hypoxia-inducible factor 1α (HIF-1α), which facilitates adaptation to the hypoxic condition characteristic of injury sites. In turn, upregulation of HIF-1α generates pseudohypoxic conditions and induces upregulation of the osteogenic differentiation genes COL I, RUNX2, and OCN.^91^ Other studies have also reported that Ti_3_C_2_T_*x*_ is a suitable hydrogel additive for supporting periodontal regeneration, as it inhibits bacterial growth, attenuates inflammation, and promotes bone regeneration (Figure 4iid).^58^

##### 4.1.2.3 Tissue Penetration in Osteomyelitis

Due to the limited tissue penetration depth of NIR lasers, MXene-based treatments relying on NIR lasers can be more challenging for deeper bone diseases such as osteomyelitis, an infection often caused by drug-resistant bacteria. In these cases, MXene-based SDT can be employed to eradicate pathogenic bacteria via ROS production. For instance, a porphyrin metal–organic framework containing a Schottky junction, modified with Ti_3_C_2_T_*x*_ nanosheets, demonstrated excellent antibacterial activity and bone regeneration upon activation with SDT.^92^ Similarly, He et al. (2024) designed an ultrasound-responsive Schottky junction composed of vanadium tetrasulfide-loaded Ti_3_C_2_T_*x*_ MXene for the treatment of MRSA-induced bone infection and bone regeneration. Upon ultrasound activation, vanadium generated ROS to achieve bacterial clearance; however, vanadium exhibited mild toxicity toward BMMSCs. Loading vanadium onto MXene reduced this inhibitory effect and supported bone regeneration by promoting osteogenic differentiation.^93^

#### 4.1.3 Neuronal Regeneration

Injuries to the peripheral or central nervous system often result in loss of function, prompting extensive research into strategies that promote cellular structure reconstruction and functional recovery. Various biomaterials have been used to support neural repair, and thanks to their extraordinary physicochemical properties, MXenes have proven promising for these applications.^94^ Therefore, their potential role in neural regeneration is currently being extensively studied.

##### 4.1.3.1 Stem Cell Differentiation and Growth

Many neurological disorders are treated using electrical stimulation to accelerate neuronal regeneration, and MXene-based therapies have shown potential to further enhance neuronal repair and functional recovery. Moreover, MXenes can stimulate the differentiation of neural progenitor/stem cells (NSCs), and MXene-infused hydrogel scaffolds provide a substrate for neuronal growth, migration, and neurite outgrowth (Figure 4iiia).^95^ For example, neurons cultured *in vitro* on a Ti_3_C_2_T_*x*_ MXene composite exhibited more branch points and longer neurites; immunofluorescence staining for the presynaptic protein synapsin-1 and postsynaptic protein PSD95 indicated that Ti_3_C_2_T_*x*_ MXene did not alter synapse development or density compared with neurons grown on tissue culture polystyrene. *In vitro* electrical stimulation of the Ti_3_C_2_T_*x*_ MXene resulted in increased NSC adhesion, more terminal extensions, and more S-phase cells, indicating increased NSC proliferation.^96^

To evaluate the effect of Ti_3_C_2_T_*x*_ MXene on NSCs, Li et al. (2022) measured the currents of voltage-gated Na^+^, K^+^, and Ca^2+^ ion channels using a patch-clamp experiment. The authors found that exposure to MXene composites increased the current through Ca^2+^ ion channels, contributing to longer neurites and neuronal firing, but not through Na^+^ or K^+^ ion channels, indicating no changes in the passive membrane component. Thus, although Ti_3_C_2_T_*x*_ MXene did not increase the density of the synaptic network, it increased synaptic release, thereby increasing synaptic transmission (Figure 4iiib,iiic).^59^ More recently, Wei et al. (2024) found that 3D Ti_3_C_2_T_*x*_ MXene Matrigel-basement membrane, which contains growth factors to support cellular growth - hydrogels provide an ECM-like growth area for the NSCs and promote proliferation and neuronal differentiation.^97^

To evaluate the MXene-activated mechanisms of NSC differentiation, Zhu et al. (2023) coated aligned poly­(l-lactide) nanofibers with Ti_3_C_2_T_*x*_ MXene and, after observing NSC proliferation and differentiation into astrocytes and neurons, assessed NSC gene expression using RNA sequencing. They found that adding MXene activated both the PI_3_K/Akt (phosphoinositide 3-kinase/protein kinase B) and calcium signaling pathways. The PI_3_K/Akt pathway, which includes the *Ghr*, *Prlr*, and *Lpar4* genes, is responsible for the proliferation and self-renewal of NSCs, while the calcium pathway, which includes the *Trhr*, *Cysltr2*, and *Hrh1* genes, directs NSC differentiation into astrocytes and neurons.^98^

##### 4.1.3.2 Central and Peripheral Nervous System Regeneration

MXenes have also been tested for use in central and peripheral nervous system injuries and regeneration. In one example, Ti_3_C_2_T_*x*_ MXene loaded with the phosphatase and tensin homologue inhibitor vanadium bisperoxovanadium promoted neuronal survival and regrowth after ischemia/reperfusion injury in ischemic brain tissues by upregulating the Akt pathway and reducing inflammation via M2 macrophage modulation.^99^ In a rat model of spinal cord injuries, a methyl acrylated gelatin and MXene composite loaded with NSCs was transplanted into the injury site. The loaded NSCs successfully differentiated into neurons and glial cells, exhibiting increased Ca^2+^ transient frequency, which enhanced the presence of neurofilament-positive cells, oligodendrocytes, and newborn neurons at the injury site.^100^

##### 4.1.3.3 Hearing Loss Improvement

Another promising application of MXenes is for patients with hearing loss, which otherwise cannot be self-healed because hair cells and spiral ganglion neurons have limited regenerative capacity. Currently, the most effective treatment for total hearing loss is cochlear implants, which convert sound into an electrical signal that stimulates spiral ganglion neurons.^101^ However, any true physiological repair of hearing loss would require the neurites of spiral ganglion neurons to develop and distribute onto regenerated inner ear hair cells. Liao et al. (2022) applied the low-frequency electrical stimulation transduced by a cochlear implant to a Ti_3_C_2_T_*x*_ MXene–matrigel hydrogel and found that their treatment increased growth cone development, spiral ganglion neurite length and growth, and filopodia number.^101^

Moreover, a separate study using an MXene-matrigel composite found that MXene treatment maintained normal proliferation and growth of cochlear organoids while upregulating hair cell-specific genes. Treatment with the MXene-matrigel composite specifically increased the abundance of phosphorylated Akt, GSK3β, and S6, suggesting that the composite promoted hair cell differentiation by potentiating the PI3K/Akt/mTOR/HIF1α signaling pathway for cell survival and proliferation (Figure 4iiid).^60^

##### 4.1.3.4 Retinal Regeneration

Retinal degeneration, including retinitis pigmentosa, macular degeneration, retinal dystrophy, and diabetic retinopathy, remains challenging to treat through conventional physiological approaches, although emerging studies suggest that MXenes could offer new therapeutic possibilities. In general, complete vision loss due to retinal degeneration is incurable, and retinal cells are highly vulnerable to oxidative stress.^102^ One promising treatment currently under study is retinal progenitor cell transplantation. Tang et al. (2023) found that Nb_2_CT*_x_* MXene activated with NIR-II laser exposure induced neuronal differentiation of retinal progenitor cells by activating the mitogen-activated protein kinase and PI_3_K/Akt pathways. At the same time, MXene treatment inhibited astrocyte-like differentiation, suppressed ROS formation, and exhibited antioxidant activity. Moreover, Nb_2_CT*_x_* MXene increased the expression of rhodopsin, β3-tubulin, and protein kinase C-α while reducing the expression of the retinal glial marker glial fibrillary acidic protein. These effects, combined with reduced stress-induced pathogenesis, led to successful retinal regeneration both *in vitro* and *in vivo*.^103^

#### 4.1.4 MXenes in Cardiac Tissue Engineering

Cardiovascular diseases remain a leading cause of death worldwide, accounting for millions of fatalities annually.^104,105^ One major condition, myocardial infarction (MI), results from obstruction of blood flow to the heart muscle, causing tissue injury and cell death. Over time, the damaged myocardium is often replaced by fibrous scar tissue, which impairs the heart’s electrical signaling and pumping function, increasing the risk of arrhythmias.^105,106^ While heart transplantation remains the definitive treatment for advanced MI, donor shortages and complications from lifelong immunosuppression limit its availability. Consequently, researchers and clinicians are exploring alternative therapeutic strategies to repair and regenerate damaged heart tissue. Cardiac tissue engineering offers a promising approach to restoring the structure and function of the injured myocardium.^106^ Since normal heart function depends on synchronized electrical communication between cardiomyocytes, biomaterials used in cardiac repair must facilitate electrical signal conduction and promote intercellular connectivity.^107^ MXenes have emerged as a cornerstone of cardiac tissue engineering, as they can bridge nonconductive scar regions, synchronize cardiomyocyte contractions, and restore physiological electrophysiological activity. Their excellent electrical conductivity, mechanical strength, tunable surface chemistry, and biocompatibility make them ideal for designing scaffolds that mimic the electro-mechanical microenvironment of native myocardium.^108^ One of the first demonstrations involved 3D-printed Ti_3_C_2_T*_x_*/polyethylene glycol (PEG) composite patches. In this study, aerosol-jet printing patterned MXene nanosheets onto hydrogels that supported the growth of human induced pluripotent stem cell-derived cardiomyocytes (iCMs). This platform enhanced cell alignment, increased expression of mature cardiac genes (MYH7, SERCA2, TNNT2), and improved synchronized beating and conduction velocity compared to controls.^109^ The engineered MXene framework provided efficient pathways for electrical coupling, illustrating how nanoscale conductivity cues can drive tissue-level functional organization. MXene-incorporated conductive hydrogels have also been designed to mimic the MI both electrically and mechanically. For instance, conductive and adhesive hydrogels containing Ti_3_C_2_T_*x*_ have demonstrated potential in promoting cardiomyocyte maturation and reducing pathological outcomes following MI. Applying MXene-containing composite to beating mouse hearts *in vivo* with MI lowered pathogenesis by improving cardiomyocyte function and adhesion to the epicardium.^110^ Similarly, a silk fibroin–hyaluronic acid hydrogel doped with Ti_3_C_2_T_*x*_ MXene enabled uniform electrical distribution, supported cardiomyocyte elongation, and improved contractility *in vitro*.^111^ Likewise, collagen-MXene biohybrids exhibited mechanical compliance and biocompatibility comparable to those of native myocardium, thereby fostering cell adhesion and electrical coupling.^108^ Future research directions involve combining MXenes with 3D bioprinting and stem-cell-laden hydrogel systems to fabricate patient-specific cardiac patches, as well as developing injectable MXene nanocomposites for minimally invasive myocardial delivery.

While these advances underscore the strong potential of MXenes in next-generation cardiac tissue engineering, they also reflect broader challenges that extend across regenerative medicine applications. In wound and diabetic wound healing, it remains unclear whether the dominant therapeutic drivers are electrical stimulation, redox modulation, or their synergy, particularly in complex chronic wound environments. Similarly, in bone regeneration, the mechanisms linking MXene degradation, ion release, immunomodulation, and osteogenesis remain largely correlative, and the performance of MXene-based scaffolds in large, load-bearing defects is not well established. In neural and cardiac tissue engineering, long-term functional recovery, electrical safety, and arrhythmogenic risks remain insufficiently explored.
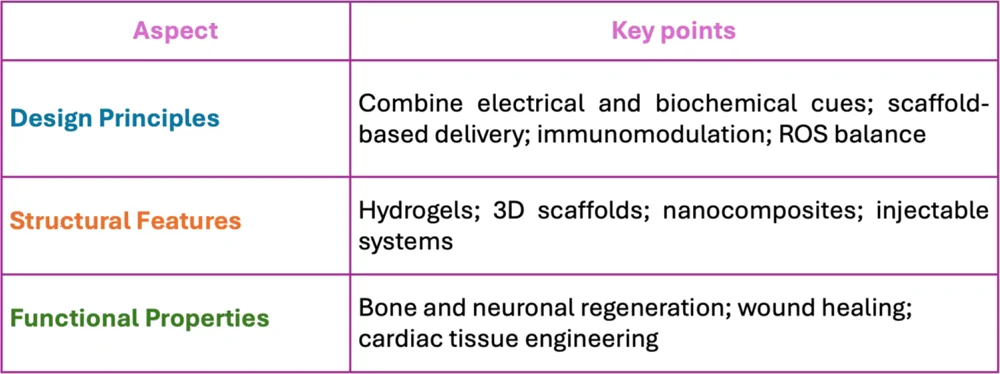


## 5 Next-Generation Anticancer Strategies

Cancer, which is characterized by uncontrolled and irregular cell proliferation and complex pathophysiology, remains one of the leading causes of death worldwide.^112^ The incidence and severity of many cancers have only continued to increase with population growth and aging, but definitive cancer treatment remains elusive.^113^ Indeed, the effectiveness of current treatment modalities for cancer, such as oncologic surgery, chemotherapy, and radiotherapy, is limited by the complexity and heterogeneity of different cancers (Figure 5a).^114–117^

**Figure 5:**
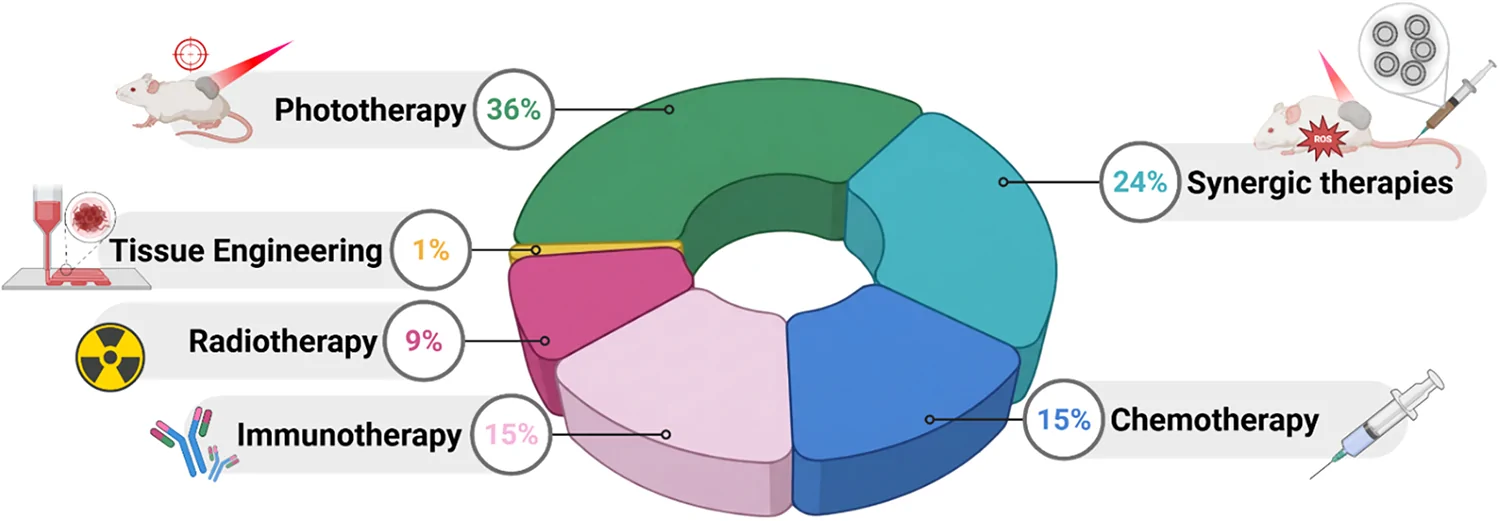
Percentage distribution of MXene-based publications in cancer therapies. The donut chart summarizes the distribution of published MXene-related studies across major cancer therapeutic modalities. Percentages represent the relative proportion of MXene publications reporting applications in phototherapy (36%), synergistic/combinatorial therapies (24%), chemotherapy (15%), immunotherapy (15%), radiotherapy (9%), and tissue engineering–based approaches (1%). The data highlight the dominant role of phototherapy and combination strategies, emphasizing the versatility of MXenes as multifunctional platforms for cancer treatment. Bibliometric data were retrieved from Scopus. (Created with BioRender.com).

In light of these limitations, there has been a growing shift toward alternative and complementary therapeutic strategies, as illustrated in Figure 5. The donut chart illustrates the distribution of published MXene-based cancer studies across major therapeutic modalities, with phototherapy accounting for 36% of the total. Synergistic approaches that combine multiple treatment modalities follow at 24%, reflecting efforts to enhance therapeutic efficacy and overcome resistance. Chemotherapy and immunotherapy each represent 15% of reported studies, while radiotherapy accounts for 9%, and tissue engineering strategies comprise only 1%. Together, this distribution highlights the growing focus on phototherapy and multimodal treatments, consistent with broader trends toward more targeted, efficient, and personalized cancer therapies.

Furthermore, invasive malignancy, mutation sensitivity of cancer cells, the lack of early screening methods, the inability to apply targeted therapies, and the general inadequacy of current medical approaches remain as barriers to the complete elimination of cancer.^118,119^

Carcinogenesis arises from a combination of internal and external factors, including nutrition, lifestyle, genetic mutations, oxidative stress, hypoxia, and exposure to radiation or pollution.^120^ Carcinogenic factors disrupt cell homeostasis.^121^ Cancer cells also lose genetic stability, enabling uncontrolled growth and proliferation, as well as the overexpression of proteins newly produced via gene rearrangement.^122^ As a result, each cancer has a unique set of genetic mutations that accumulate with tumor growth, and drugs used in cancer treatment may not have the same effect on every patient.^123^ In addition, cancer treatments have been limited by their often severe side effects, such as immunosuppression, and a lack of knowledge of the mechanisms required for controlled, targeted or nontargeted drug delivery.^124,125^

Next-generation nanomaterials have the potential to overcome many of these limitations and thereby revolutionize cancer treatment.^126^ These materials, which enable the development of targeted cancer treatment strategies, offer significant advantages over conventional therapies: they can reach and specifically target tumor cells while also increasing pharmaceutical efficacy and minimizing side effects.^127–131^ These unique properties of nanomaterials, combined with advances in nanotechnology, have led to the emergence of interdisciplinary research into new, nanomaterial-based anticancer treatments.^132–135^ In this context, researchers have designed alternative nanomaterials and explored diverse nano systems for cancer treatment, including MXene-based therapies.^136–143^

### 5.1 MXenes in Therapeutic Strategies

Cancer remains a leading cause of global mortality, characterized by uncontrolled cellular proliferation, genetic instability, and significant treatment limitations due to tumor heterogeneity and drug resistance.^144,145^ Current therapies—including surgery, chemotherapy, and radiotherapy—often lack specificity, resulting in severe side effects and limited effectiveness.^146,147^ Recently, MXenes have emerged as promising candidates for addressing these challenges, offering targeted, effective, and minimally invasive therapeutic approaches.^148^

MXenes exhibit unique properties that are advantageous for anticancer applications, including high photothermal conversion efficiency, biodegradability, biocompatibility, and the apability for surface modification and therapeutic loading.^149–152^ These feature makes them ideal platforms for advanced therapeutic strategies such as PTT, photodynamic therapy (PDT), SDT, chemodynamic therapy (CDT), immunotherapy, and synergistic approaches.^129,148,153,154^ Furthermore, MXenes have demonstrated considerable potential in radiotherapy enhancement, promoting radiosensitization and ROS-mediated DNA damage in cancer cells.^155^ The incorporation of MXenes into 3D-printed constructs further highlights their innovative applications in personalized cancer treatments, providing platforms for modeling tumor complexity and facilitating targeted drug delivery.^156^

In this context, the development of more controllable and targeted treatment modalities to minimize the side effects associated with conventional chemotherapy and radiotherapy has become increasingly important.^157^ Emerging approaches, including PTT, PDT, SDT, and immunotherapy, as well as their combinatorial use, have shown significant promise in improving therapeutic efficacy.^158–163^

#### 5.1.1 Photothermal Therapy

Phototherapy has emerged as a promising, more targeted cancer treatment strategy.^164,165^ For example, anticancer phototherapy techniques rely on NIR light, which uses less energy, penetrates more deeply, and causes less tissue damage than visible light.^149,166,167^ In PTT, the phototherapy agent heats up upon exposure to NIR; this approach exploits the thermal sensitivity of tumor cells, leading to cell death after ablation.^168,169^ The photothermal effect in MXenes comes from their ability to efficiently absorb light across a wide wavelength range and then quickly transform that energy to heat through interactions between electrons and phonons. Low thermal conductivity of MXenes contributes to the temperature rize of MXene flakes. This causes localized hyperthermia and the destruction of tumor cells (Figure 6ia).

**Figure 6:**
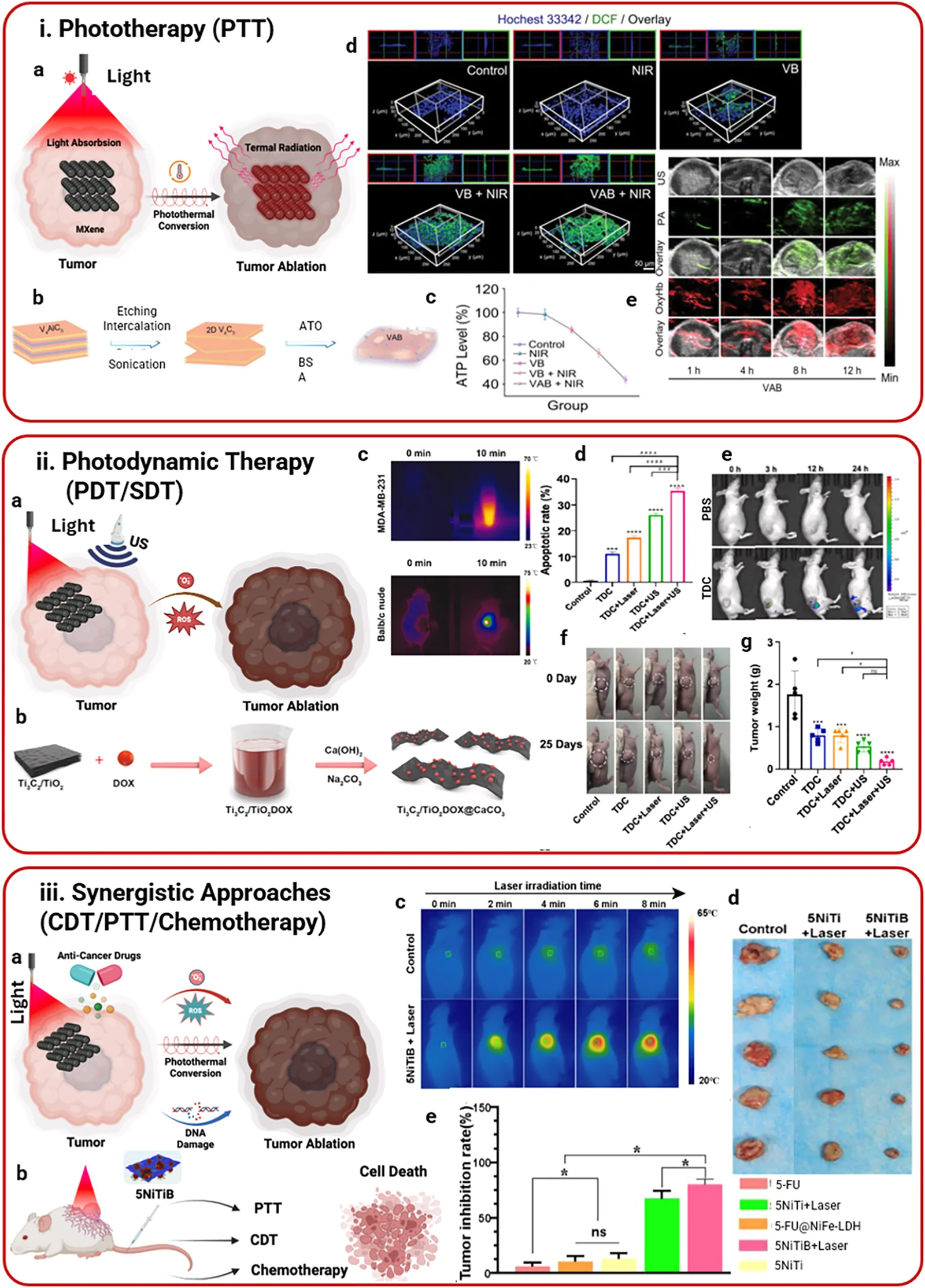
MXenes in therapeutic strategies. **i**. **Photothermal therapy**. (a) Schematic representation of MXene-mediated PTT for tumor ablation. Upon light irradiation, MXene nanosheets localized in the tumor absorb optical energy and convert it into heat through photothermal conversion, generating localized hyperthermia that destroys cancer cells (created with BioRender.com). (b) Illustration of the V_4_C_3_/atovaquone@BSA nanoplatform (VAB), a 2D vanadium-based multifunctional MXene design. (c) Intracellular ATP levels in cancer cells after different treatments (mean ± SD, *n* = 3). (d) Detection of intracellular ROS using the DCFH-DA staining in 3D multicellular tumor spheroids to evaluate antitumor activity. (e) PAI of mice bearing 4T1 tumors with VAB to assess *in vivo* tumor targeting and therapeutic response. Reprinted with permission from Zhao et al. 2024.^183^ Copyright 2024 Wiley-VCH GmbH. **ii**. **Photodynamic therapy (PDT/SDT)**. (a) Schematic illustration of combined PDT and SDT using MXene nanomaterials. Under light and ultrasound stimulation, MXenes generate ROS via photodynamic and sonodynamic effects, inducing oxidative stress and tumor cell destruction (created with BioRender.com). (b) Preparation of a nanopharmaceutical based on CaCO_3_-coated Ti_3_C_2_T_*x*_/TiO_2_ loaded with doxorubicin (DOX) (TDC). (c) Infrared thermal imaging demonstrating the PTT of TDC in vitro in MDA-MB-231 cells and in vivo in tumor-bearing mice. (d) Apoptosis assay in 4T1 breast cancer cells after synergistic treatment with TDC. (e) Fluorescence imaging of tumor-bearing mice injected with TDC at 3, 12, and 24 h to examine DOX biodistribution. (f) Representative tumor photographs before and after treatment in nude mice with breast cancer model. (g) Mean tumor weights of different treatment groups. Reprinted with permission from Luo et al. 2024.^192^ Copyright 2024 Elsevier B.V.. **iii**. **Synergistic approaches (CDT/PTT/Chemotherapy)**. (a) Schematic representation of a multimodal MXene-based platform integrating PTT, PDT, and chemotherapy for enhanced cancer treatment (created with BioRender.com). (b) Illustration of the 5-FU@NiFe-LDH/Ti_3_C_2_T_*x*_/BSA nanoplatform (5NiTiB), combining NiFe-LDH-mediated CDT, MXene-driven PTT, and 5-FU chemotherapy. (c) Infrared thermal images showing the in vivo photothermal response of 5NiTiB. (d) Representative images of tumors excised on day 21 from mice treated with 808 nm laser irradiation, 5NiTi, or 5NiTiB compared with the control group. (e) Tumor inhibition rates for different treatment groups (mean ± SD, *n* = 3). Reprinted with permission from Xu et al. 2024.^204^ Copyright 2024 Elsevier B.V..

Given the growing interest in MXenes for biomedical applications, their potential role in anticancer interventions has attracted considerable research attention. In a foundational study, Lin et al. reported that Ti_3_C_2_T_*x*_ nanosheets exhibit strong NIR absorption and used them as a photothermal agent for *in vivo* photothermal ablation in a mouse tumor model.^170^ This work paved the way for the subsequent use of MXenes as a phototherapy agent against tumors.^171–174^ An effective photothermal agent must convert light energy to heat, be biocompatible, and be completely biodegradable. MXenes meet both of these requirements^175^ as 50% of MXenes can be easily degraded within about 1 month.^176^ In addition, MXenes exhibit minimal cytotoxicity and remain stable in physiological systems, making them promising photothermal agents. To prevent the minimal cytotoxicity caused by ions adsorbed on the MXene surface, Lim et al. proposed conjugating MXene to biopolymers as a surface modification.^30^

MXene-based nanosystems have also been developed for anticancer drug delivery applications by customizing MXene surfaces with biocompatible and bioactive materials.^177,178^ These nanosystems make MXenes an ideal material for cancer therapy, as they combine high photothermal conversion efficiency, specificity, and biocompatibility with the ability to enable controlled and targeted drug release. For example, studies are increasingly evaluating the photothermal destruction of tumors using superparamagnetic MXenes with high photothermal conversion efficiency.^179–181^ To enhance efficacy and shorten treatment duration, pulsed NIR^21^ laser irradiation can be employed, yielding rapid therapeutic effects within 40–60 s at ultralow MXene concentrations (<5 μg mL^–1^). This pulsed, ultrafast photothermal regimen not only achieves efficient melanoma cell ablation but has also demonstrated in vivo safety, highlighting a promising avenue for fast and effective melanoma therapy.^182^

Zhao et al. tested the antitumor activity of a V_4_C_3_T_*x*_ and atovaquone (V_4_C_3_T_*x*_ /ATO@BSA, VAB)-based photothermal agent encapsulated with bovine albumin (Figure 6ib, ic).^183^ Atovaquone binds to mitochondrial cytochrome reductase, thereby interrupting the electron transport chain and subsequent ATP production. Impaired mitochondrial respiration, in turn, inhibits tumor cell proliferation. Zhao et al. found that, upon irradiation with 808 nm NIR light, their composite effectively reduced ATP levels in thermally affected breast cancer tumor cells *in vivo* (Figure 5ic). Glutathione levels were lowest in mice treated with the MXene composite under NIR radiation, regardless of the presence of atovaquone. However, inclusion of atovaquone led to reduced mitochondrial activity, as detected by JC-1 staining. Furthermore, the V_4_C_3_ component exhibited 61% photothermal conversion efficiency upon NIR irradiation, resulting in greater ROS production than non-MXene materials (Figure 6id). Overall, this vanadium-based MXene nanomaterial showed promise for tumor eradication, had minimal side effects, and also resulted in improved imaging of the tumor region through *in vivo* photoacoustic monitoring (Figure 6ie).^177,183^

Despite effective in vitro tumor-cell ablation, in vivo translation remains limited by the lack of active targeting. Following systemic administration, MXenes can distribute broadly, yielding subtherapeutic intratumoral concentrations and necessitating strategies that promote selective tumor accumulation. Surface functionalization with targeting moieties—such as peptides, antibodies, or aptamers—can endow MXenes with molecular recognition, enhancing binding and uptake by cancer cells. For example, polydopamine (PDA)–modified MXenes conjugated with anti-CEACAM1 antibodies selectively recognize CEACAM1-positive melanoma cells, enabling targeted tumor ablation.^184^ Also, MXenes, modified with aptamer against transmembrane glycoprotein mucin 1, demonstrated high selectivity and NIR-based PTT effectiveness toward MCF-7 cancer cells.^185^ In another approach, Ti_3_C_2_ nanosheets were sequentially coated with Fe_3_O_4_ nanoparticles to produce magnetically actuated microrobots (“MXBOTs”).^186^ Under a rotating magnetic field, MXBOTs navigate along predefined paths; integration of Ti_3_C_2_ imparts robust photothermal conversion and improves photoacoustic contrast, conferring theranostic capability. Collectively, such active-targeting strategies can overcome the limitations of passive delivery, improving biodistribution and delivery efficiency, particularly in atypical lesion sites with complex in vivo fluid dynamics.

#### 5.1.2 Photodynamic Therapy

PDT involves biotoxicity-mediated apoptosis of tumor cells by inducing ROS production as a result of light-stimulated activation of photosensitizers in tumors.^187,188^ While PDT has a therapeutic effect in superficial lesions, it cannot penetrate deep tissues and is known to cause phototoxicity.^189,190^ SDT, an emerging alternative to photodynamic therapy, can achieve deep tissue penetration but kills only targeted cancer cells, without harming healthy tissue, through the use of an ultrasound-activated sonosensitizer capable of producing ROS.^191^ ROS generation in MXene-based systems is primarily driven by photo- or sono-induced electron excitation, followed by energy or electron transfer to molecular oxygen, resulting in the formation of reactive species such as singlet oxygen (^1^O_2_) and hydroxyl radicals (^•^OH), which induce oxidative damage in cancer cells (Figure 6iia). In a breast cancer treatment study, Luo et al. developed Ti_3_C_2_T_*x*_ /TiO_2_ nanosheet composites loaded with doxorubicin and encrusted with CaCO_3_ (Figure 6iib).^192^ This composite was shown to respond synergistically to photoacoustic imaging (PAI)^193^ and NIR-based photothermal activation, and its anticancer effects were then demonstrated *in vitro* in MDA-MB-231 cells and *in vivo* in tumor-bearing BALB/c nude mice (Figure 6iic). *In vitro*, the combined photothermal and sonodynamic treatment inhibited tumor cell viability and induced apoptosis, while *in vivo*, the synergistic therapy further enhanced tumor suppression by increasing the population of M1-type macrophages (Figure 6iid). Furthermore, synergistic photothermal and sonodynamic treatment with this MXene composite enhanced the therapeutic effect of doxorubicin against tumors (Figure 6iie), with mice receiving the synergistic treatment showing the greatest reductions in tumor volume and weight (Figure 6iif,iig).

#### 5.1.3 Chemodynamic Therapy

A novel treatment modality, CDT, destroys cancer cells by using low valence transition metal ions to catalyze reactions that produce hydroxyl radicals.^194^ He et al. used Ti_3_C_2_T_*x*_ MXene as a photothermal agent and nanocarrier for breast cancer treatment.^195^ They enhanced the efficacy of their treatment by integrating HSP90 antisense oligonucleotides and the CDT therapy agent doxorubicin into their Ti_3_C_2_T_*x*_ nanoplatform via layered absorption followed by surface modification using hyaluronic acid. The resulting nanocomposite thereby delivered antisense oligonucleotides and doxorubicin when activated via NIR light. This approach represents a potential new adjuvant treatment strategy to inhibit the proliferation and migration of drug-resistant cancer cells. It has been difficult to achieve favorable results with CDT therapy alone, so CDT therapy is often used in combination with other treatment options.^196,197^

#### 5.1.4 Synergistic Approaches

In many cases, cancer treatment will involve holistic therapies that combine multiple, synergistic treatment modalities to overcome challenges such as the complexity of tumor structure, drug resistance, and cancer recurrence.^81,198–200^ In chemodynamic therapy, MXenes facilitate Fenton or Fenton-like reactions by catalyzing the conversion of endogenous H_2_O_2_ into highly toxic hydroxyl radicals, thereby amplifying oxidative stress within the tumor microenvironment (Figure 6iiia). Ongoing studies exploring the use of MXene-based nanomaterials in unconventional cancer therapies continue to produce highly promising strategies.^201^ For example, Tan et al. investigated the *in vitro* effects of Ti_3_C_2_T_*x*_ and UV irradiation on cytotoxicity, apoptosis, caspase activation, nuclear condensation, and cell morphology in MCF-7 breast cancer cells.^202^ They found that low concentrations of Ti_3_C_2_T_*x*_ had minimal effects but the addition of UV light synergistically increased cytotoxicity. Moreover, the combined MXene-UV treatment decreased tubulin expression and distribution in the cancer cells. These results indicated that combining Ti_3_C_2_T_*x*_ and UVB irradiation may be a promising therapeutic strategy for inducing apoptotic cell death in breast cancer. Shao et al. demonstrated that titanium nitride quantum dots (Ti_2_N-QDs) are biocompatible and photothermally efficient under NIR-I (808 nm) and NIR-II (1064 nm) laser irradiation.^176^ They also reported that their Ti_2_N-QDs facilitated anticancer PTT in the NIR-I/II window without any toxicity to host cells, with significant clustering in tumors observed only 4 h after *in vivo* injection.

In refractory cancers such as osteosarcoma, safely but synergistically proportioning multiple treatment modalities has proven challenging.^203^ To address this challenge, Xu et al. designed a synergistic cancer therapy that balances photothermal, chemodynamic, and chemotherapy-based approaches (Figure 6iiib).^204^ First, they developed a multifunctional, trimodal 5-FU@NiFe-LDH/Ti_3_C_2_T_*x*_ /BSA nanoplatform (5NiTiB) for osteosarcoma treatment that maximizes therapeutic effects while minimizing side effects. The chemotherapeutic agent 5-fluorouracil (5-FU) was encapsulated in the nanoplatform via a combination of ion exchange and electrostatic adsorption, transforming the NiFe-LDH base into nanospheres. The alkaline, positively charged NiFe-LDH was then electrostatically coupled with the negatively charged Ti_3_C_2_T_*x*_ MXene, which stimulated pH-responsive drug release in the slightly acidic tumor microenvironment, reduced the side effects of positive charge accumulation in physiological environments, and prevented premature drug degradation. Coating the MXene composite with bovine serum albumin (BSA) further ensured a safe trimodal drug-delivery platform for antiosteosarcoma therapy. Xu et al. then investigated the therapeutic efficacy of their 5NiTB composite in an in vivo mouse model following intravenous administration of different formulations, including 5FU, 5FU@NiFe-LDH, 5NiTi, 5NiTi + Laser, and 5NiTiB + Laser, with laser activation performed at 808 nm. The temperature in the tumor region increased slowly with NIR irradiation (Figure 6iiic), and, after 2 min of irradiation, photothermal ablation of the tumor was achieved with a temperature near 45 °C. Tumor volume and weight were then monitored for 12 days. There was no significant difference in tumor size reduction between the different treatment groups, but NIR irradiation significantly reduced tumor volume and weight in mice treated with 5NiTi and 5NiTiB (Figure 6iiid). Moreover, treatment with 5NiTiB produced the highest tumor suppression rate (Figure 6iiie). In conclusion, this synergistic antitumor system provided pH-sensitive chemotherapeutic release that was further enhanced through photothermal and chemodynamic therapies. In another study, drug-free nanoconstructions of Ti_3_C_2_T_*x*_ MXene nanosheets (TC-MX) were prepared using ultrasonic exfoliation and then deposited with copper oxide (Cu_2_O/MX, CO-MX) using an in situ reduction method.^205^ These engineered nanosheets demonstrated in vivo ablation of mammary carcinoma in BALB/c mice via synergistic PTT and CDT.

#### 5.1.5 Immunotherapy

Complex systems in cancer therapies are often limited by toxicity, drug resistance, high dosage requirements, and off-target effects. This situation fuels the search for more effective alternatives that can functionalize therapeutic approaches, enhance effects on target cells, and eliminate off-target effects.^206^ Immunotherapy is a new generation of cancer treatment that includes immune blockade, T-cell-based adaptive immunotherapy, cytokine delivery, and cancer vaccines, especially for metastasized and recurrent cancers (Figure 7ia).^207,208^ The immunotherapeutic mechanism of MXene-based systems is primarily associated with the induction of immunogenic cell death (ICD), where tumor cell destruction leads to the release of damage-associated molecular patterns (DAMPs), tumor-associated antigens, and pro-inflammatory signals. These signals promote dendritic cell maturation and antigen presentation, which in turn activate cytotoxic T lymphocytes and initiate a systemic antitumor immune response. Additionally, MXenes can serve as nanocarriers for immune adjuvants, further enhancing immune activation and amplifying therapeutic efficacy. In this regard, immunotherapy involves various strategies to activate the immune system to naturally eliminate cancer cells.^209–214^ Recent advances in anticancer therapies have accelerated the transition from immuno-monotherapy to multimodal, synergistic anticancer treatment modalities, and MXenes have the potential to play a prominent role in these treatments.^177,215,216^ Zhang et al. fabricated MXenes@PEI nanosheets (MXP) by functionalizing the surface of Ti_3_C_2_T_*x*_ using the cationic polymer polyethylenimine (PEI).^217^ These nanosheets were then used for combined dendritic cell immunotherapy and NIR-activated PTT to induce tumor immunogenic cell death. In this combination treatment, MXP-mediated PTT enabled the production of “premium” antigens to promote dendritic cell maturation, with fragments of tumor cells forming endogenous nanoparticles. Moreover, the addition of the antigen ovalbumin and its agonists (CpG-ODN) was reported to enhance dendritic cell activation as an exogenous nanostructure. In B16-OVA tumor-bearing mice, MXP-mediated PTT and dendritic cell immunotherapy showed a strong therapeutic effect. Cheng et al. developed an ultrasonically controlled, photocatalytically active 2D bismuth molybdate (Bi_2_MoO_6_, BMO) MXene (BMO-MXene) to induce ferroptosis, a form of targeted and immunotherapy-sensitive iron-dependent programmed cell death caused by excessive lipid peroxidation.^218^ The production of ROS by the BMO-MXene was enhanced via a Schottky heterojunction (Figure 7ib). Under ultrasound excitation, BMO-MXene promoted apoptosis in ovarian cancer cells *in vitro* more effectively than treatment with BMO, MXene, or ultrasound alone (Figure 7ic). The authors correspondingly tested for necroptosis, pyroptosis, apoptosis, and ferroptosis biomarkers to determine the type of cell death induced by the ultrasound-activated BMO-MXene treatment. Ferroptosis biomarkers were significantly downregulated upon ultrasound-activated BMO-MXene treatment, whereas changes in general cell death biomarkers were negligible (Figure 7id). These findings suggest that ferroptosis in ovarian cancer cells facilitates dendritic cell maturation and stimulates antitumor immunity by activating immunogenic cell death (Figure 7ie). Overall, the BMO-MXene acted as an effective 2D piezoelectric nanoagonist to inhibit ovarian cancer under ultrasound stimulation.

**Figure 7:**
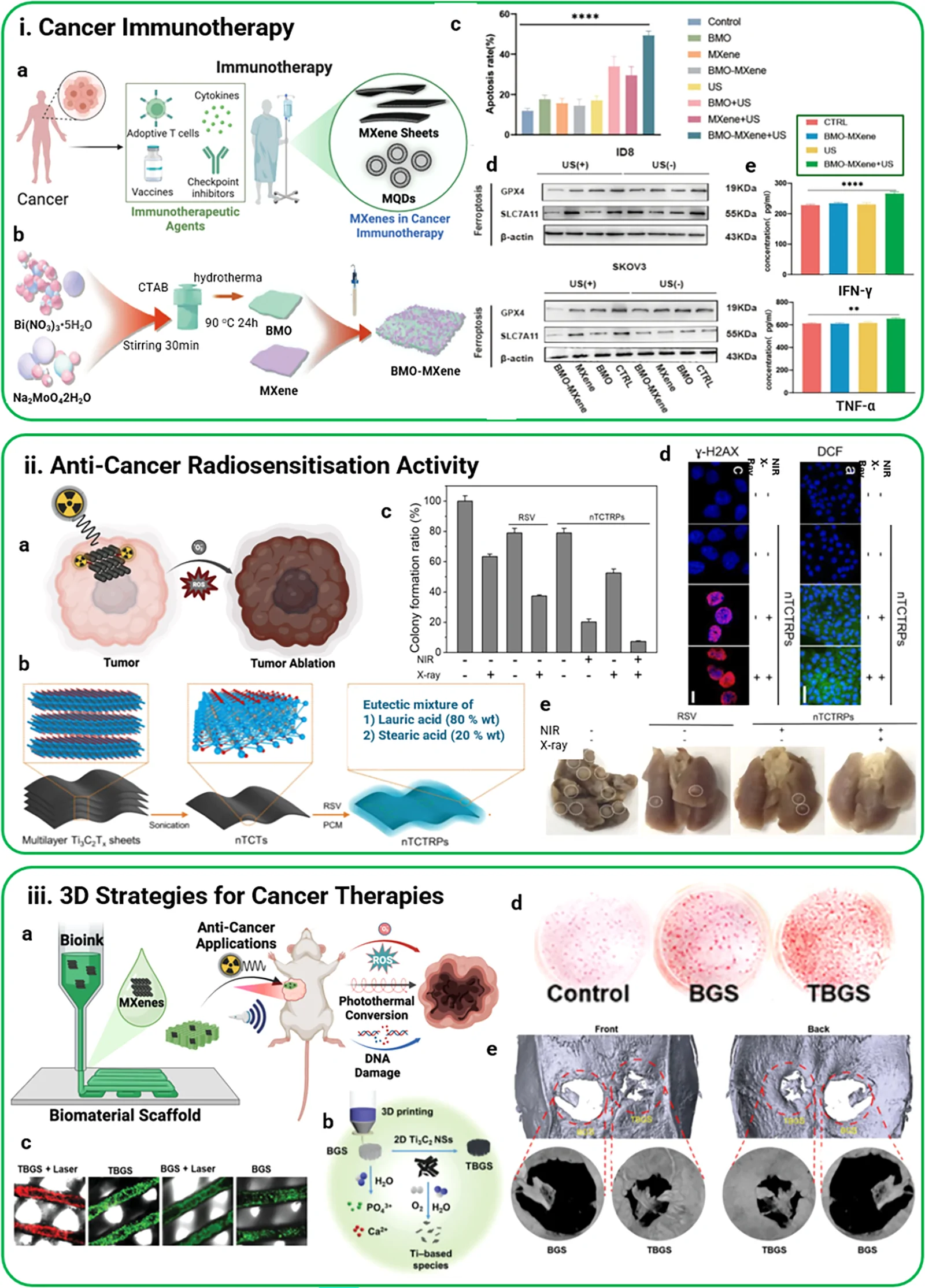
Anticancer applications of MXenes. **i**. **Cancer immunotherapy**. (a) Schematic of MXene-integrated cancer immunotherapy, highlighting their role in augmenting treatment efficacy. (Created with BioRender.com). (b) Schematic illustration of the synthesis process of Bi_2_MoO_6_(BMO)-MXene. (c)­Apoptosis assay showed different effects of treatment groups, including BMO, MXene and US treatment. (d) Changes due to BMO-MXene combinational treatment, including expression levels of ferroptosis biomarkers in ID8 cells and SKOV3 cells to determine the type of death occurring in ovarian cancer cells under US stimulation.^245^ (e) Serum IFN-γ and TNF-α levels of mice measured using ELISA from different groups. Reprinted with permission from Cheng et al. 2024.^218^ Copyright 2024 Springer Nature **ii**. **Anticancer radiosensitization activity**. (a) Schematic representation of MXene-mediated radiosensitization: ionizing radiation triggers MXene nanostructures to enhance ROS generation, inducing oxidative stress and apoptosis for effective tumor ablation (Created with BioRender.com). (b) Illustration of the fabrication of nTCTRPs. (c) 4T1 cells coincubated with nTCTRPs and/or RSV followed by quantification of colony formation rates after irradiation with 808 nm NIR light and/or X-rays. (d) Fluorescence images of 4T1 cells after incubation with nTCTRPs and 808 nm NIR and/or X-ray treatment with H2DCF-DA probe (Scale bar: 50 μm) for of ROS levels and fluorescently labeled γ-H2AX (Scale bar: 10 μm) for detection of DNA damage. (e) Lung tissues were visualized after 808 nm NIR light and/or X-ray application of a 5% glucose solution, RSV or nTCTRPs injected to 4T1 tumor-bearing mice. Reprinted with permission from Zhu et al. 2023 ^238^ Copyright 2023 Elsevier B.V.. **iii**. **Tissue engineering strategies for cancer therapies**. (a) Schematic representation of MXene-loaded bioinks used for 3D bioprinting of biomaterial scaffolds. (Created with BioRender.com). (b) Production of composite scaffold (Ti_3_C_2_T_*x*_-BG scaffold, TBGS). (c) Confocal microscopy images after propidium iodide staining of dead cells and calcein-AM staining of viable cells for viability analysis of Saos-2 on BGSs or TBGSs with different treatments (Scale bar: 200 μm). (d) Alizarin red staining images were shown for ECM mineralization in control, BGS and TBGS groups after 21 days of hBMSCs coculturing. (e) To evaluate in vivo osteogenesis, 3D reconstructions and micro-CT images of the 5 mm cranial defects were analyzed 24 weeks postimplantation. Reprinted with permission from Pan et al. 2020. Copyright 2020 Wiley-VCH GmbH.^244^

MQDs derived from 2D MXene nanosheets have demonstrated promise in anticancer applications. MQDs have unique properties relative to MXene nanosheets, including their tunability, conductivity, and surface area, and researchers can easily control MQD surface chemistry, size, and composition in ways that may facilitate specific molecular interactions.^219^ Studies have correspondingly reported that MQDs can be used with immunoadjuvant approaches to support cancer therapy.^220^ To understand the interaction between tumor cells and the tumor microenvironment in response to MQD distribution, Ceylan et al. monitored the spread of MQDs through a tumor in a mouse breast cancer model by relying on the red fluorescence emitted by the MQDs.^154^ Moreover, they used spatial transcriptomics to identify molecular and cellular changes in the tumor microenvironment after MQD administration. Sites with high MQD accumulation showed a tumor suppressor phenotype, and other immune cell types, including B cells and neutrophils, were positively affected by the MQDs.

#### 5.1.6 Immunomodulation

MXenes possess intrinsic immunomodulatory potential that enables them to regulate immune responses and reshape the tumor microenvironment through redox activity, macrophage polarization, and cytokine modulation.^221^ Unlike immunotherapy, which actively stimulates immune effector mechanisms to destroy cancer cells, immunomodulation focuses on restoring immune balance and preventing pathological inflammation or immune suppression.^63,222^ The immunomodulatory mechanism of MXenes is largely driven by their redox-active surfaces, which regulate intracellular ROS levels and influence signaling pathways involved in immune responses. By modulating oxidative stress, MXenes can suppress pro-inflammatory cytokine production while promoting anti-inflammatory pathways, leading to macrophage polarization toward the M2 phenotype and the expansion of regulatory T cells. This dynamic regulation of immune signaling contributes to the restoration of immune homeostasis and the reshaping of the tumor microenvironment. Among MXenes, Ti_3_C_2_T*_x_* has been the most widely investigated for its immunoregulatory effects.^63^ Ti_3_C_2_T*_x_* nanosheets can attenuate pro-inflammatory cytokine release while promoting anti-inflammatory signaling, resulting in improved tissue regeneration and reduced oxidative stress.^223^ A study by Rafieerad et al. (2019) demonstrated that MXenes are highly biocompatible with human peripheral blood mononuclear cells (PBMCs) at concentrations up to 1000 ng mL^–1^ after 24 h of culture, with no detectable cytotoxicity for up to 7 days. Immunomodulatory evaluations revealed a marked decrease in lymphocyte proliferation in MXene-treated groups compared with controls, indicating reduced lymphocyte activation in response to proinflammatory stimuli. Flow cytometric analysis further showed a reduction in CD4^+^ IFN-γ^+^ Th1 inflammatory cells and an increase in CD4^+^ CD25^+^ FoxP3^+^ regulatory T cells, suggesting that MXenes induce an anti-inflammatory and immunoregulatory phenotype.^63^ More recently, Ta_4_C_3_T*_x_* MXenes have emerged as next-generation immunomodulatory materials due to their high biostability, superior redox control, and minimal cytotoxicity compared to Ti-based systems. Another study by Rafieerad et al. (2021) reported the synthesis of Ta_4_C_3_T*_x_* MQDs that exhibited strong immunomodulatory properties both *in vitro* and *in vivo*.^224^ These Ta_4_C_3_T*_x_* MQDs effectively scavenged ROS and rebalanced cytokine secretion profiles by downregulating pro-inflammatory cytokines (IL-1β, TNF-α), while enhancing anti-inflammatory markers (IL-10). In an allograft vasculopathy model, treatment with Ta_4_C_3_T*_x_* MQDs reduced immune cell infiltration, restored mitochondrial function, and mitigated oxidative injury, demonstrating their ability to stabilize immune activation without broad immunosuppression. This intrinsic immunomodulatory property arises from tantalum’s high oxidation resistance and electron affinity, which allow the MQDs to act as selective ROS quenchers and immune homeostasis regulators. These findings represent the first *in vivo* demonstration of MXenes functioning as nanoscale immunoregulators, independent of drug or antigen loading.^224,225^ Another research by, Zheng et al. (2024) demonstrated that Nb_2_CT*_x_* MXene-functionalized hydrogels substantially promote macrophage recruitment, drive polarization toward the M2 (anti-inflammatory, tissue-repairing) phenotype, enhance angiogenesis, and facilitate the resolution of oxidative stress in vivo. This immunoregulatory activity improves the tissue microenvironment and supports regeneration by reducing inflammation and encouraging vascularization and neurogenesis.^24,226^ MXene-based immunomodulation offers a promising approach to reshape the immune landscape in cancer and regenerative medicine. Their tunable surface chemistry, combined with the ability to selectively modulate ROS levels and cytokine secretion, positions them as powerful next-generation platforms for immune focused nanomedicine. Continued exploration of their long-term immune compatibility, adaptive immune cross-talk, and signaling pathways will be essential for translating MXene-based immunomodulators into clinical applications.

#### 5.1.7 Radiotherapy

A separate line of research into effective anticancer therapies has focused on X-ray-based radiotherapy options,^227^ which may also be enhanced or expanded with the use of nanomaterials like MXenes.^228,229^ The conventional challenge with radiotherapy has been maintaining the dosage balance between the tumor and healthy tissue.^230^ Radiosensitive nanomaterials applied intratumorally have the potential to enhance the effective radiation dose received by tumor cells without causing harm to healthy cells.^231^ Considering the radiosensitizer effects of metal-based nanomaterials, high doses of radiation to cancer cells may cause ROS-induced DNA damage and cell death (Figure 7iia).^232–234^ The radiosensitization mechanism of MXenes primarily stems from their ability to enhance radiation-induced ROS generation and amplify DNA damage in cancer cells. Upon X-ray irradiation, MXenes facilitate energy deposition and secondary electron production, which interact with intracellular water molecules to generate highly reactive species such as hydroxyl radicals (^•^OH). These ROS induce oxidative stress and DNA double-strand breaks, ultimately leading to tumor cell apoptosis or necrosis. In addition, the high atomic number (*Z*) elements present in certain MXene compositions improve radiation absorption and increase local dose enhancement, further strengthening therapeutic efficacy. To date, MXene-based nanomaterials have shown superior radiosensitization efficiency compared to other nanomaterials like graphene oxide, achieving larger increases in intracellular ROS formation under X-ray irradiation.^235^ For example, Zimmermann et al. reported that Ti_3_C_2_T_*x*_ exhibited significant radiosensitization in human soft tissue sarcoma cells, while showing no toxic effect on adjacent healthy fibroblasts.^236^ In another study, molybdenum cluster nanoparticles showed a strong radiosensitizing effect via direct singlet oxygen generation upon X-ray irradiation in prostate carcinoma models.^237^ Zhu et al. reported a photothermally responsive, antitumor drug delivery system that used synergistic radiosensitization by Ti_3_C_2_T_*x*_ nanosheets and provided controlled delivery of natural *trans*-resveratrol (RSV) (Figure 7iib).^238^ Nanosized Ti_3_C_2_T_*x*_ nanosheets (nTCTs) act as a nanozyme that exhibits radiation-enhanced graded enzymatic activity. Using a eutectic mixture of stearic acid and lauric acid, these nanosheets can be tightly loaded with RSV (creating nTCTRPs). Synergistic *in vitro* activation of nTCTRPs with 808 nm NIR light and X-ray irradiation inhibited the growth of murine breast carcinoma cells more effectively than treatment with RSV alone (Figure 7iic).^238^ Growth inhibition was largely achieved by increasing ROS levels. Sequential irradiation with NIR light and X-ray resulted in the highest ROS concentration; increased ROS levels, in turn, caused irreparable DNA damage, as evidenced by increased expression of the DNA damage marker γ-H2AX (Figure 7iid).^238^ In *in vivo* studies using a mouse breast cancer model, treatment with free RSV decreased the number of lung metastatic nodules relative to controls, whereas no discernible lung metastasis was observed in mice treated with nTCTRPs and sequential irradiation (Figure 7iie). These in *vitro* and *in vivo* results suggest that nTCTRPs are a promising candidate for synergistic radiosensitization and tumor metastasis inhibition.^238^

#### 5.1.8 3D-Printed Constructs

The dynamic and complex nature of cancer limits experimental spatial and temporal observations, which has led to an increased interest in 3D approaches in cancer research.^239^ These approaches include the use of 3D-printed multiscale cancer models and the transplantation of 3D-printed scaffolds.^42^ Multiscale cancer modeling enhances the accuracy of prognostic predictions, refines experimental hypotheses, and better directs future research efforts.^240^ Three-dimensional models have accordingly been developed to mimic the structural complexity of cancer cells; some models include the ECM, while others do not (Figure 7iiia).^193,241^ Once developed, 3D models can replicate complex *in vivo* tumor conditions while allowing low-cost, fast, and efficient evaluations of treatment efficacy. These cancer modeling platforms also enable high-throughput screening of nanomaterials, providing deeper insights into their therapeutic performance and systemic effects.^242,243^ Perini et al. employed a breast cancer model developed on 3D-bioprinted spheroids to investigate the anticancer effect of photothermal treatment with 50 μg mL^–1^ of either few- or multilayered Ti_3_C_2_T_*x*_ MXene.^181^ They found that MXene-mediated photothermal treatment of the spheroids resulted in increased ROS concentrations and decreased cancer cell viability. Furthermore, the photothermally induced temperature increased tumor cell death inhibiting tumor progression. These results not only emphasize the anticancer activity of MXenes but also demonstrate the ease of screening MXene-based therapies in 3D cancer models rather than *in vivo*. MXenes used in cancer therapies can also be integrated into 3D-printed multifunctional scaffolds. For example, Pan et al. constructed a 3D scaffold for bone cancer therapy containing multifunctional 2D Ti_3_C_2_T_*x*_ MXene and bioactive glass.^244^ NIR-stimulated photothermal hyperthermia of this composite resulted in simultaneous bone tumor ablation and bone tissue regeneration. Specifically, the mortality rate of osteosarcoma cells (Saos-2 cell line) was higher after MXene-based photothermal treatment relative to other treatments (Figure 7iiib).^244^ Moreover, alizarin red staining of BMMSCs increased osteogenesis *in vitro* in cells that received MXene-based treatment relative to controls or those that received only bioactive glass (Figure 7iiic).^244^ In an *in vivo* model, Sprague–Dawley rats were observed and scanned with microcomputed tomography 24 weeks after implantation of the composite scaffolds. Three-dimensional reconstruction of skulls harvested from rats with critical cranial defects showed more calcified tissue in skulls that received the MXene-based composite (Figure 7iiid-e), confirming that the addition of MXene results in better regenerative outcomes than bioactive glass alone.^244^

In summary, as shown in Table 1, MXene-based therapeutic strategies have tunable surface chemistry, a layered structure, and multifunctional designs. These features enable various mechanisms, including photothermal conversion, ROS production, radiosensitization, and immune modulation. These characteristics confer substantial benefits, including elevated therapeutic efficacy, the capacity for combination therapy, and the prospect of targeted delivery. Moreover, inherent limitations—such as inadequate targeting specificity, difficulties in biodistribution, system complexity, and insufficient understanding of long-term safety—persist as significant obstacles to clinical translation. So, future efforts should be focused on smart design strategies that find a balance between biocompatibility, functionality, and translational feasibility.
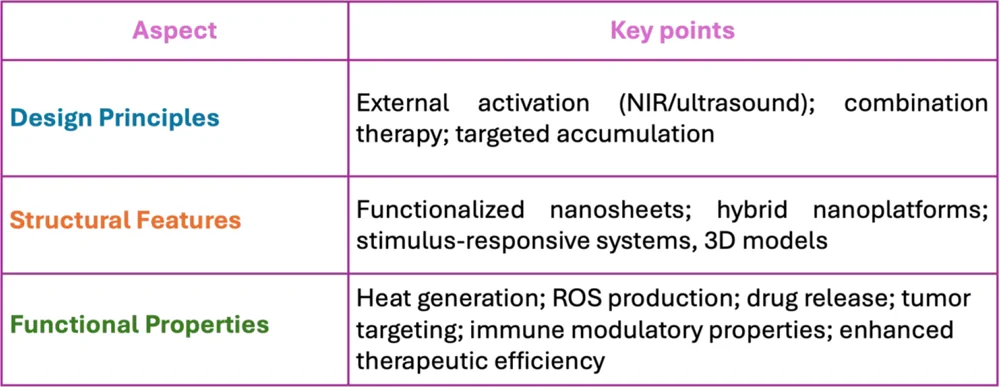


**Table 1:** Critical Analysis of MXene-Based Therapeutic Strategies in Cancer: Design Features, Mechanisms, Advantages, and Limitations

Strategy	Key MXene Design Features	Mechanism of Action	Main Advantages	Current Limitations
PHOTOTHERMAL THERAPY (PTT)	High NIR absorption, large surface area, surface functionalization (PEG, PDA, antibodies)	Light-to-heat conversion → localized hyperthermia → tumor ablation	Rapid response, high efficiency, theranostic potential, minimal invasiveness	Limited NIR penetration, off-target heating, insufficient active targeting, unclear long-term fate
PHOTODYNAMIC/SONODYNAMIC THERAPY (PDT/SDT)	Hybrid MXene composites (Ti_3_C_2_/TiO_2_, CaCO_3_ coatings), ROS-generating systems	ROS generation under light/ultrasound → oxidative stress → apoptosis	Noninvasive activation, synergistic therapy potential, imaging compatibility	Hypoxia reduces ROS, limited light penetration (PDT), system instability, targeting challenges
CHEMODYNAMIC THERAPY (CDT)	Metal-ion loaded MXenes, catalytic nanoplatforms, HA-modified systems	Fenton/Fenton-like reactions → ^•^OH generation → cancer cell death	Tumor microenvironment responsiveness, synergy with PTT/chemotherapy	Low endogenous H_2_O_2_, weak standalone efficacy, metal toxicity concerns
SYNERGISTIC THERAPIES	Multifunctional nanoplatforms (PTT + PDT + CDT + chemo), hierarchical design	Multimechanistic tumor killing (heat + ROS + drugs)	Overcomes resistance, enhanced efficacy, reduced dosage	Complex design, scalability issues, regulatory challenges
IMMUNOTHERAPY	MXene-immune adjuvant conjugates (PEI, CpG), antigen delivery systems	Immune activation, dendritic cell maturation, ICD induction	Systemic antitumor immunity, metastasis control	Risk of immune overactivation, immunotoxicity, limited long-term data
IMMUNOMODULATION	Redox-active MXenes (Ti_3_C_2_T*_x_*, Ta_4_C_3_T*_x_* MQDs), cytokine-regulating surfaces	ROS scavenging, macrophage polarization (M1/M2), immune balance	Anti-inflammatory control, microenvironment regulation	May suppress needed immune response in cancer, unclear immune cross-talk
RADIOTHERAPY ENHANCEMENT	High-Z MXenes, nanozyme activity, ROS amplifiers	Radiosensitization → enhanced ROS → DNA damage	Improved radiotherapy efficiency, selective tumor sensitization	Dose optimization unclear, biodistribution issues, potential toxicity
3D-PRINTED CONSTRUCTS	MXene-integrated scaffolds, bioactive composites, tunable architectures	Tumor modeling + localized therapy + regeneration	Biomimetic modeling, dual therapy-regeneration, screening platform	Translation gap (in vitro → in vivo), fabrication complexity, long-term safety

## 6 MXenes in Bioimaging

Bioimaging, a technique that offers noninvasive and real-time application in biological processes, is critical for early stage cancer diagnosis, localization, staging and detection of cancer recurrence.^246^ Since it is noninvasive, it does not affect living processes and allows obtaining information about the 3D structure without damaging the sample. MXene-based nanomaterials play a role in bioimaging and diagnostics thanks to their nanoscale structure, intermolecular interaction, and unique properties. MXenes can be effectively used with various techniques such as magnetic resonance imaging (MRI), PAI,^193^ computed tomography (CT) and fluorescence imaging, thanks to their excellent physicochemical properties.^247^

### 6.1 High-Dimensional Imaging

Our previous work addressed a critical challenge in nanobiomedicine: the need for high-resolution, label-free detection and imaging of 2D materials such as MXenes within single cells and tissues. To meet this need, we introduced a novel strategy called LINKED (Label-free sINgle-cell tracKing of 2D matErials by mass cytometry and MIBI-TOF Design), combining single-cell mass cytometry by time-of-flight (CyTOF) and multiplexed ion beam imaging by time-of-flight (MIBI-TOF) (Figure 8ia). We designed MXenes (Nb_4_C_3_T_*x*_, Mo_2_Ti_2_C_3_T_*x*_, and Ta_4_C_3_T_*x*_) containing specific transition metals that could be directly identified by mass cytometry without the need for chemical functionalization. Our approach enabled simultaneous detection of nanomaterials and in-depth profiling of cellular and tissue responses. The method demonstrated successful label-free identification and quantification of MXenes in 15 primary human immune cell subpopulations, along with assessments of their biocompatibility, cytokine production, and modulation of immune cell interactions (Figure 8ib). Moreover, *in vivo* studies in mice revealed the biodistribution of MXenes across major organs, including the liver, spleen, lungs, and blood, without signs of toxicity. Importantly, MIBI-TOF was applied for the first time to detect nanomaterials spatially within tissues (Figure 8ic).^248^

**Figure 8:**
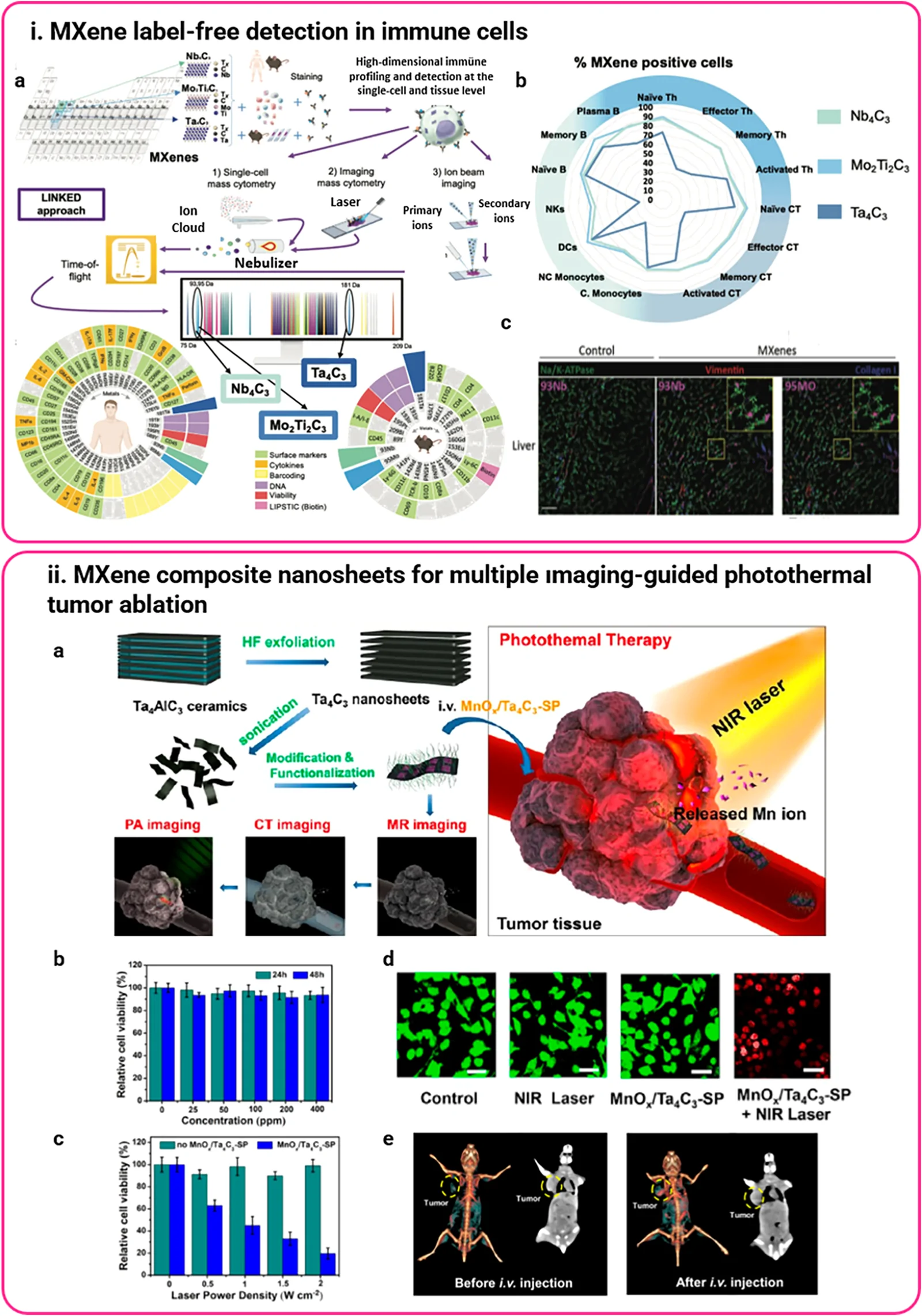
MXene in bioimaging. **i**. **MXene label-free detection in immune cells**. (a) Label-free single-cell tracking of 2D materials by mass cytometry and MIBI-TOF (LINKED approach). CyTOF-visible elements (gray in the periodic table) were selected to synthesize biocompatible MXenes (Nb_4_C_3_T_*x*_, Mo_2_Ti_2_C_3_T_*x*_, Ta_4_C_3_T_*x*_). A schematic shows their detection in cells and tissues via single-cell mass cytometry, imaging mass cytometry, and ion-beam imaging. Mass cytometry detects stable isotopes from 75–209 Da; colored images indicate current CyTOF metal tags. Nb_4_C_3_T_*x*_, Mo_2_Ti_2_C_3_T_*x*_, and Ta_4_C_3_T_*x*_ are detected in the ^93^Nb, ^95^ Mo, and ^181^Ta channels. MXenes are compatible with commercial tags: 48 isotopes were used for human ex vivo and mouse *ex vivo/in vivo* experiments, and 76 probes for viability, cytokines, CD markers, Pd/Cd barcoding, DNA staining, and biotin detection. (b) PBMCs were incubated for 24 h with Nb_4_C_3_T_*x*_ (260 nm), Mo_2_Ti_2_C_3_T_*x*_ (240 nm), and Ta_4_C_3_T_*x*_ (160 nm) (50 μg mL^–1^ each). CyTOF analysis across 15 immune cell types is shown as a spider chart of MXene-positive cells (%) per subpopulation. (c) MIBI-TOF imaging of tissues from control and MXene-injected mice stained with metal-labeled antibodies shows MXene signals (purple) across all tissues in the ^93^Nb and ^95^Mo channels. Scale bar: 50 μm. All experiments were performed in triplicate (mean ± SD). Reprinted with permission from Fusco et al., 2022.^248^ Copyright 2022 Wiley-VCH GmbH. **ii**. **MXene composite nanosheets for multiple imaging-guided photothermal tumor ablation**. (a) Workflow representation. Tantalum carbide (Ta_4_C_3_T_*x*_) MXene surface modified with manganese oxide nanoparticles (MnO_*x*_/Ta_4_C_3_T_*x*_) for contrast-enhanced CT, PTT, MR and PAI. (b) Relative viabilities of 4T1 cells after incubation with MnO_*x*_/Ta_4_C_3_T_*x*_–SP composite nanosheets (25, 50, 100, 200, and 400 μg mL^–1^) for 24 and 48 h. Error bars were based on the standard deviations (s.d.) of five parallel samples. (c) Relative viabilities of 4T1 cells after incubation with MnO_*x*_/Ta_4_C_3_T_*x*_–SP composite nanosheets (200 μg mL^–1^) followed by the exposure to 808 nm laser at different laser power densities (0, 0.5, 1, 1.5, and 2.0 W cm^–2^). (d) CLSM images of 4T1 cancer cells stained by calcein AM and PI after the varied treatments, including control, NIR laser only, MnO_*x*_/Ta_4_C_3_T_*x*_–SP only and MnO_*x*_/Ta_4_C_3_T_*x*_–SP + NIR laser. (e) *In vivo* 3D reconstruction CT (left) and contrast (right) images of mice before and after i.v. administration MnO_*x*_/Ta_4_C_3_–SP composite nanosheets (20 mg kg^–1^, 100 μL) for 2 h. Reprinted with permission from Dai et al. 2017.^264^ Copyright 2017 American Chemical Society.

### 6.2 Fluorescence Imaging

Fluorescence imaging is favored for its high selectivity, low cost, and fast results.^249^ Efforts to develop fluorescent probes for biocompatible and long-term fluorescence imaging, especially those that can detect changes, such as pH or concentration differences, are still ongoing. The ultrathin thickness, chemical stability, hydrophilicity, conductivity, and physicochemical properties of MXene-based nanomaterials have brought them into focus as suitable candidates for electrochemical sensing and fluorescence imaging. Moreover, it has been reported that MXene QDs have been fabricated from MXene nano scaffolds by reducing them to smaller sizes, showing better luminescence and being more suitable for biological imaging due to their small size.^250,251^ Guan et al. used MXene modified with folic acid (FA)-PEG-NH_2_ combined an open fluorescent nanoprobe with fluorescent aptamers to investigate the changes of cellular ATP and GTP during mild-photothermal therapy (mPTT).^252^ The result showed that the fluorescent nanoprobe was biocompatible and capable of specific recognition. Moreover, they reported that ATP and GTP levels increased more in cancer cells than in normal cells during mPTT. In another study, Yang et al, produced an MXene (Nb_2_CT*_x_*) quantum dot with abundant functional groups by ultrasonication in tetrapropylammonium hydroxide (TPAOH). The result demonstrated that the produced Nb_2_CT*_x_* Fe^3+^ exhibited fluorescence through imaging cells and hydrogel implants by selective fluorescence detection.^253^

### 6.3 Magnetic Resonance Imaging

MRI is an imaging technology that provides 3D cross-sectional images for the structural delineation of soft tissues with its nonradiation, noninvasive, and high-resolution image capability.^254^ Increasing concerns about the toxicity of MRI contrast agents to the kidneys in recent years have prompted the development of biosafe, low-toxicity contrast agents to improve MRI quality and specificity.^255^ At this point, MXenes, which stand out with their structural and functional properties such as low toxicity and biocompatibility, offer the opportunity to be used simultaneously for both therapeutic and imaging purposes. Dai et al. reported the design of composite MnO_*x*_/Ti_3_C_2_T_*x*_ MXene for biocompatible and highly effective theranostic applications against cancer.^256^ Composite MnO_*x*_/Ti_3_C_2_T_*x*_ MXenes were developed as a theranostic agent for MRI and PA dual-modality imaging-guided PTT against cancer. The MnO_*x*_ component offers T1-weighted MRI imaging sensitive to the tumor microenvironment, while the high photothermal conversion performance gives MnO_*x*_/Ti_3_C_2_T_*x*_ the desired contrast-enhanced PA imaging capability as well as efficient tumor ablation. In another study, Song et al. reported an ultrathin Ti_3_C_2_T_*x*_ MXene film with a thickness of ≈20 nm, cross-linked via poly­(3,4-ethylene dioxythiophene):polystyrene sulfonate (PEDOT:PSS), capable of simultaneously sensing electrophysiological signals, performing high-fidelity MRI and functional near-infrared spectroscopy (fNIRS) imaging with minimal interference from magnetic and optical fields.^257^ Apart from cross-linking to stabilize the MXene film, PEDOT:PSS also shortens the interlayer distance for efficient charge transfer and high transparency. As a result, this film showed a combination of high electroconductivity and transparency (11 000 S cm^–1^ @89%). Moreover, due to its ultrathin feature, it is compatible with fNIRS and MRI and enables multimodal cognitive-neural monitoring in long-term use.

### 6.4 Photoacoustic Imaging

An effective contrast agent for PAI,^193^ a noninvasive and nonionizing imaging technology, must have excellent photothermal conversion ability to generate a signal that contrasts with the PAI signal generated by the surrounding tissue.^258^ At this point, MXenes with excellent photothermal conversion properties have become the center of interest for PAI.^259^ Zada et al. synthesized V_2_CT_*x*_ MXene nanosheets with 90% efficiency using a green delamination method based on algal extraction. This approach offers a highly efficient, low-cost, and environmentally friendly method for producing V_2_CT_*x*_ MXene, which otherwise suffers from limitations, such as low photothermal conversion in PAI and challenging production conditions. As a result, these synthesized V_2_CT_*x*_ nanosheets were reported to be an effective photothermal agent for PA and MRI, and PTT of cancer in *in vitro* and *in vivo* studies, showing high absorption in the NIR region with a photothermal conversion efficiency of ∼48%.^260^

### 6.5 Computed Tomography

CT imaging is one of the popular imaging modalities due to its spatial resolution, deep tissue penetration and noninvasiveness, which relies on tissue absorption of radiation as the basis for obtaining 3D images.^261^ CT scans, which are an auxiliary imaging technique in the diagnosis and staging of various diseases, are frequently used in high-resolution diagnosis of metastatic cancers.^262^ While the expectation from common contrast agents (CA) for CT scans is that they are biocompatible and have a short circulation time in the blood, they are usually iodinated compounds with the disadvantage of high toxicity and risk of renal failure. Therefore, while the search for nontoxic and biocompatible CAs continues, studies using MXenes by combining various components for CT scans attract attention.^263^ Tantalum carbide (Ta_4_C_3_T_*x*_) MXene exhibits high atomic number and X-ray attenuation coefficient, making it an attractive option for CT.^264,265^

Dai et al., reported the development of a surface-functionalized tantalum carbide (Ta_4_C_3_T_*x*_) MXene nanocomposite, modified with manganese oxide nanoparticles (MnO_*x*_), for use in multimodal imaging and PTT.^264^ The schematic workflow outlines the synthesis process, starting from the exfoliation of Ta_4_AlC_3_ ceramics and subsequent surface modification to enable enhanced imaging capabilities and therapeutic performance (Figure 8iia). The composite enables contrast-enhanced CT, magnetic resonance (MR), and PAI, while also serving as an efficient photothermal agent under NIR^21^ laser irradiation. *In vitro* cytotoxicity assays reveal that the MnO_*x*_/Ta_3_C_3_–SP nanosheets exhibit low toxicity to 4T1 cancer cells at various concentrations in the absence of NIR light (Figure 8iib). However, upon NIR laser exposure, a marked reduction in cell viability is observed in a laser power-dependent manner, confirming effective photothermal ablation (Figure 8iic). Fluorescent live/dead staining further supports the synergistic effect of the composite and laser irradiation in inducing cell death (Figure 8iid). *In vivo* CT imaging demonstrates significant tumor accumulation and enhanced imaging contrast postinjection, validating the composite’s potential for real-time tumor tracking and image-guided therapy (Figure 8iie). Together, these results demonstrate the feasibility of using MnO*_x_*/Ta_4_C_3_ MXene-based nanomaterials as multifunctional platforms for integrated cancer diagnosis and therapy, combining biocompatibility, multimodal imaging capability, and photothermal therapeutic efficacy.^264^ Furthermore, Liu et al. reported the use of a platform based on Ta_4_C_3_T_*x*_ MXene and superparamagnetic iron oxide functionalization (Ta_4_C_3_T_*x*_ -IONP-SPs composite MXenes) for simultaneous MRI/CT dual-modality imaging and photothermal hyperthermia in breast cancer.^266^ Although MXenes have rapidly emerged as promising platforms for bioimaging, substantial knowledge gaps persist that constrain their translational potential and clinical relevance. In high-dimensional and label-free imaging approaches, such as CyTOF and MIBI-TOF, further studies are needed to define detection limits, quantitative accuracy, and scalability across different MXene families and complex human tissues, particularly under pathological conditions. For fluorescence imaging, although MXene quantum dots show promising luminescence and biocompatibility, the mechanisms underlying fluorescence generation, long-term photostability, and potential quenching in biological environments remain insufficiently understood. In MRI, MXene-based contrast agents have shown reduced toxicity and theranostic potential, yet their relaxivity, degradation kinetics, and clearance pathways have not been systematically benchmarked against clinically approved agents, raising unresolved safety and regulatory questions. Similarly, in PAI and CT, most studies emphasize contrast enhancement and proof-of-concept tumor visualization, while long-term biodistribution, off-target accumulation, and dose-dependent imaging–toxicity trade-offs remain poorly characterized, highlighting the need for integrated structure–property–biological performance frameworks and longitudinal *in vivo* studies.
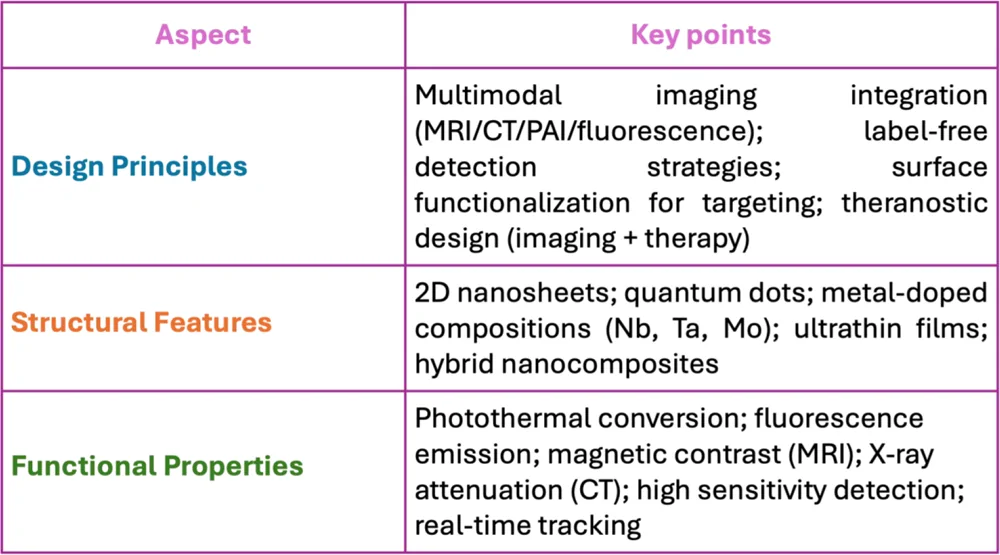


## 7 MXenes as Smart Antimicrobial Materials

After the first publication in 2016, where Ti_3_C_2_T_*x*_ has shown significant antibacterial effects against Gram-negative *E*.*coli* and Gram-positive *B*. *Subtilis* (Figure 9ib), MXenes considered as promising candidates for the development of antibacterial strategies and as a promising tool against antibiotic-resistant infections.^267^ This property is valuable not only in biomedical contexts such as wound healing or tissue regeneration but also in industrial settings, including food preservation and the remediation of contaminated water. Several subsequent reports demonstrated strong antibacterial activity across in *vitro* and *in vivo* models, including skin infection and wound healing.^268^ Furthermore, MXenes can be functionalized or combined with other materials to improve their antimicrobial performance. Surface modifications of MXenes can introduce additional antimicrobial agents or improve the stability and biocompatibility of the material.^269^ These functionalized MXenes can then be incorporated into wound dressings, implant coatings, and drug delivery systems to prevent bacterial infections and promote tissue regeneration. Ti_3_C_2_T_*x*_-based polymers, such as sodium alginate-, polyethylenimine-, and Ag/poly­(vinyl alcohol)-based aerogels, have exhibited robust antibacterial activity against pathogens like *E. coli* and the Gram-positive *S. aureus*. Ding et al. (2024) developed a nanoantibacterial spray by embedding ICG-loaded ZIF-90 (a zinc-based MOF) into Ti_3_C_2_T_*x*_ MXene. Upon NIR irradiation, the spray showed strong anti-*S. aureus* and anti-*E. coli* activity *in vitro* and *in vivo*, accelerating wound healing via combined photothermal and photodynamic effects plus Zn^2+^-mediated membrane disruption (Figure 9ic).^270^ In a related polymer system, Ag/Ti_3_C_2_T_*x*_ functions as a nanoenzyme that elevates intracellular ROS.^271^ These antimicrobial properties of Ti_3_C_2_T_*x*_ are not limited to its 2D nanosheet structure: Ti_3_C_2_T_*x*_ MXene MQDs are also considered effective antimicrobial agents. *In vitro* and *in vivo* experiments confirm the exceptional antibiofilm, antioxidant/anti-inflammatory, and wound-regeneration properties of a probiotic bioheterojunction (P-bioHJ) combining *Lactobacillus rhamnosus* with (MQDs)/FeS.^272^ Even antibiotic-resistant strains, including MRSA and vancomycin-resistant Enterococcus, are susceptible to Ti_3_C_2_T_*x*_ photothermal antibacterial activity.^273^ Likewise, ICG-loaded Ti_3_C_2_T_*x*_ and a non-cross-linked chitosan/Ti_3_C_2_T_*x*_ hydrogel achieved MRSA clearance under NIR irradiation via combined PTT and PDT effects.^274^

**Figure 9:**
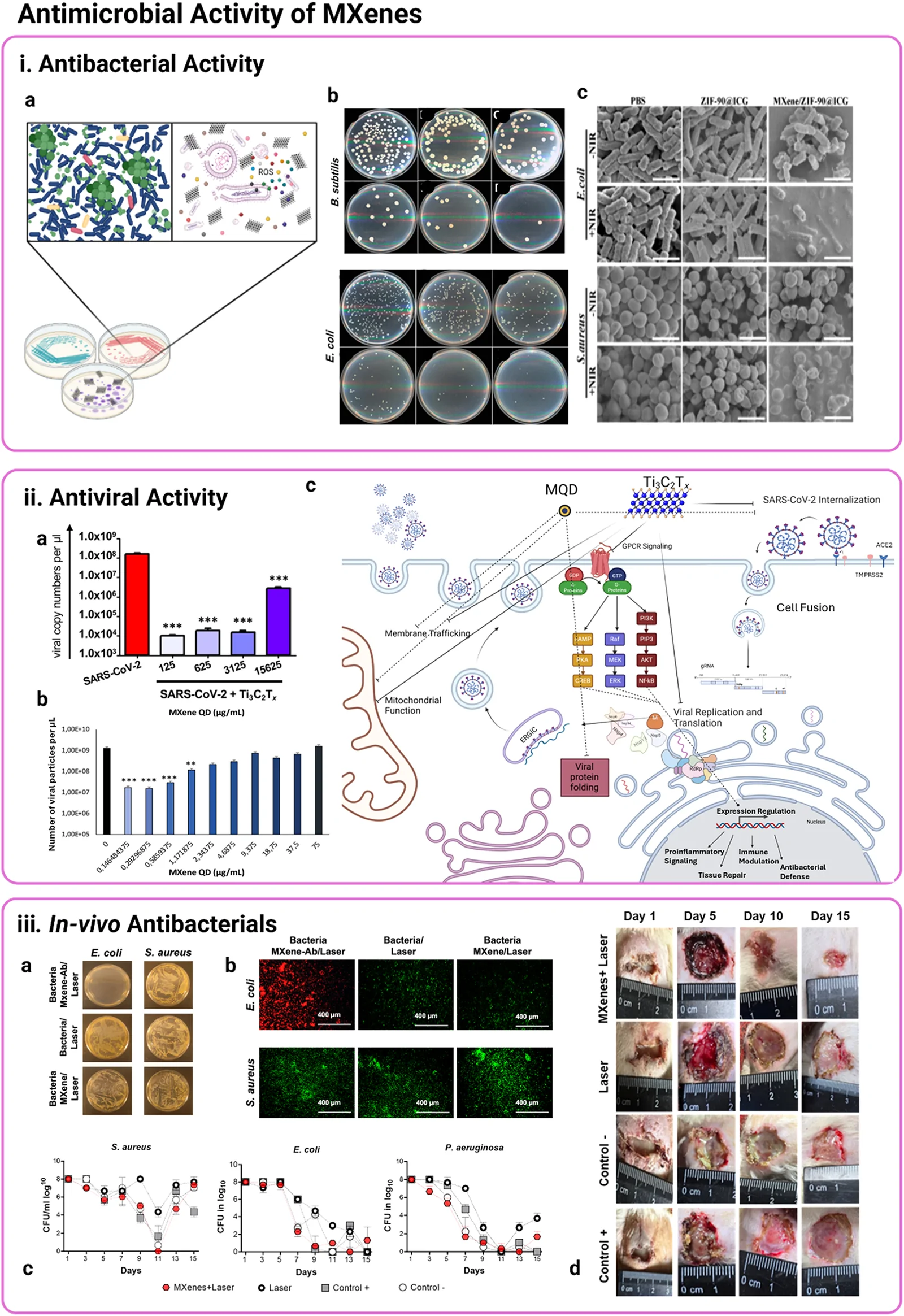
Antimicrobial activity of MXenes. **i**. **Antibacterial activity**. (a) Schematic representation of the bacterial clearance with MXenes leading to bacterial clearance via ROS induction and bacterial membrane disruption (Created with BioRender.com). (b) The clearance of *E. coli* and B. subtilis seeded agar plates in with an increasing dose of Ti_3_C_2_T_*x*_. Reprinted with permission from Rasool et al., 2016.^267^ Copyright 2016 American Chemical Society. (c) SEM images of bacterial death and morphological changes. Reprinted with permission from Ding et al. 2024.^270^ Copyright 2024 American Chemical Society. **ii**. **Antiviral activity**. (a) The antiviral activity of Ti_3_C_2_T_*x*_ is shown. Reprinted with permission from Unal et al., 2021.^279^ Copyright 2021 Elsevier B.V.. (b) The low doses of MQD also showed antiviral activity. Reprinted with permission from Yilmazer et al., 2023.^280^ Copyright 2023 Wiley. (c) Schematic representation of MQD and Ti_3_C_2_T_*x*_ interaction with the inhibition of protein responsible for viral internalization, transcription and translation, protein folding, membrane trafficking and mitochondrial function in SARS-CoV-2 infection (Created with BioRender.com). **iii**. **Targeting antibacterial delivery and** ***in vivo*** **effectiveness**. (a) Representative images of ABIN3027591-negative *S. aureus* and ABIN3027591-positive *E. coli* growth on Mueller–Hinton agar plates after 24 h of incubation, (b) and corresponding live/dead fluorescence images obtained after targeted Ti_3_C_2_T*_x_*-PDA-Ab PTT. (c) Dynamics of bacterial survival after MXene-based PTT of rat purulent wound with (d) macroscopic images of wound defects at different time intervals of the experiment. Reprinted with permission from Korniienko et al., 2026.^285^ Copyright 2026 American Chemical Society.

Other MXenes, including delaminated Nb_2_CT_*x*_ and Nb_4_C_3_T_*x*_, have also demonstrated antibacterial activity against *E. coli* and *S. aureus* by inducing oxidative stress. Notably, Nb_4_C_3_T_*x*_ exhibited a stronger oxidative effect than Nb_2_CT_*x*_, highlighting the role of MXene atomic structure in antimicrobial applications.^275^ Zong et al. (2023) enhanced the modest antibacterial performance of fluorine-free Mo_2_CT_*x*_ by constructing an MoO_*x*_@Mo_2_CT_*x*_ nanonetwork, where oxygen-vacancy-rich MoO_*x*_ was formed in situ on the MXene scaffold. Upon ultrasound activation, the nanonetwork showed strong bactericidal activity against *S. aureus*, with RNA sequencing used to probe the killing mechanism. In mouse models of deep wounds and bone defects, MoO_*x*_@Mo_2_CT_*x*_ achieved complete MRSA clearance and supported tissue regeneration.^276^ In other research, deoxyribonuclease I loaded vanadium carbide MXene (DNase-I@V_2_C) demonstrated effective biofilm elimination that open new perspectives for diabetic wound treatment and infection prevention.^277^

The antibacterial activity of MXenes also extends beyond bacterial clearance: they inhibit biofilm formation, making them excellent candidate materials for medical implants coating.^278^ For example, a probiotic bioheterojunction containing Ti_3_C_2_T_*x*_-derived MQDs disrupted the formation of extracellular polymeric substance, which is crucial for biofilm formation.^272^ Similarly, Nb_2_CT_*x*_ MXene titanium plate-based clinical implants both (i) induce biofilm detachment by downregulating bacterial attachment pathways and promote bacterial clearance by modulating bacterial metabolic signaling pathways, thereby inhibiting growth and proliferation.^278^

Antiviral studies on MXenes are developing, but the evidence base remains limited. In our work, Ti_3_C_2_T_*x*_, Ta_4_C_3_T_*x*_, Mo_2_Ti_2_C_3_T_*x*_, and Nb_4_C_3_T_*x*_ were evaluated against SARS-CoV-2; Ti_3_C_2_T_*x*_ and Mo_2_Ti_2_C_3_T_*x*_ showed antiviral activity (Figure 9iia). TiO_2_ alone had no effect, suggesting Ti_3_C_2_T_*x*_ activity is driven by MXene surface chemistry rather than titanium. Proteomics and CyTOF across 17 immune subpopulations indicated potential mechanisms involving viral entry, GPCR signaling, membrane trafficking, replication, mitochondrial function, and metabolism, alongside reduced pro-inflammatory cytokine production.^279^ Similarly, Yilmazer et al. (2023) reported that Ti_3_C_2_T_*x*_-derived MQDs suppressed SARS-CoV-2 replication at low dose—likely by disrupting viral protein folding/replication, and modulated immunity by altering the TNF-α response (Figure 9iib–c).^280^

The biological properties of MXenes, including antibacterial activity, depend strongly on their physicochemical characteristics, such as sheet size, surface charge, and atomic structure, as well as on synthesis-related factors, including the etching chemistry and the reagents used for delamination and intercalation.^26^ In general, MXene antimicrobial activity is attributed to two main mechanisms. First, under external stimuli such as light, MXenes can generate ROS. MXenes can induce ROS generation on the bacterial membrane by combining surface reactivity and electron transfer mechanisms. The transition-metal layers can catalyze redox reactions with oxygen, generating superoxide (O_2_^–•^) and downstream species such as hydrogen peroxide (H_2_O_2_) and hydroxyl radicals (^•^OH). In addition, the sharp edges and high conductivity of MXene nanosheets can allow direct contact with the bacterial membrane, disrupting the electron transport chain and promoting electron leakage, further driving ROS formation inside/near the cell.^45^ These ROS, including singlet oxygen and hydroxyl radicals, induce oxidative damage in bacterial cells and ultimately lead to cell death. MXenes can produce ROS efficiently because of their distinctive electronic properties and surface chemistry. As a result, they can act as effective clearance agents against a broad range of bacterial species.^281^ Second, MXenes can physically disrupt bacterial cell membranes (Figure 9ia). Their interaction with the membrane may cause structural damage, increased permeabilization, and leakage of intracellular contents, which culminates in bacterial death. For example, during photothermal antibacterial treatment, MXenes can function as “nanothermal blades,” inactivating bacterial proteins, inducing DNA cross-linking, and compromising membrane integrity.^282,283^ Due to these properties, MXenes exhibit a broad-spectrum antimicrobial effect without inducing acquired bacterial resistance.

However, despite encouraging findings, several studies have failed to detect intrinsic antibacterial activity in pristine MXenes. In many cases, bactericidal effects emerge only after compositing or surface functionalization, for example, with graphene oxide (GO), oxygen-doped graphitic carbon nitride (O-g-C_3_N_4_), tungsten trioxide (WO_3_), or bismuth oxychloride (BiOCl).^284^ This inconsistency is further compounded by the generally high cytocompatibility of MXenes, which challenges mechanisms invoking “nano-knife” membrane disruption or ROS-mediated killing; indeed, several reports suggest mammalian cells may be more susceptible to such damage than bacteria. Recent work also indicates that Ti_3_C_2_T_*x*_, Mo_2_TiC_2_T_*x*_, and Mo_2_Ti_2_C_3_T_*x*_ generate ROS at levels insufficient to damage bacteria.^45^ A key determinant of antibacterial performance is the composition and purity of MXene nanosheets parameters that were not consistently controlled in the early stages of MXene research. More recent studies, using a range of *in vitro* and *in vivo* models, indicate that high-quality, stoichiometric, minimally oxidized MXenes (e.g., Ti_3_C_2_T_*x*_, Nb_2_CT_*x*_, V_2_CT_*x*_, and Ti_3_CNT_*x*_) show negligible antimicrobial activity under standard, nonactivated conditions.^285^ However, MXenes possess distinctive photothermal properties that can be leveraged for highly effective bacterial ablation when externally triggered, particularly when paired with antibody-based targeting to concentrate the material at the bacterial surface (Figure 9iiia,b). This targeted photothermal strategy enables rapid and selective bacterial killing and provides a framework for next-generation MXene antimicrobials designed as precision “nanotools” for efficient bacterial eradication. This approach demonstrated effective *in vivo* bacterial eradication in a murine purulent wound model infected with a mixed bacterial inoculum (*S. aureus*, *E. coli*, and *Pseudomonas aeruginosa*) and was accompanied by significantly accelerated wound closure following NIR irradiation of MXene-loaded wounds (Figure 9iiic,d).

Clinically, MXene-based nanomaterials are being explored for next-generation wound dressings and implant surface coatings, where their multimodal antimicrobial mechanisms can prevent infections and promote tissue integration.^51,286^ Industrially, MXenes offer exciting possibilities in water treatment and antifouling systems, as their inherent antibacterial properties can be harnessed in membranes and coatings to disinfect water and resist biofilm formation.^287^ Looking forward, MXenes stand out as precision-engineered nanomaterials with tunable features, from surface terminations to optical and electronic properties, that can be adjusted to optimize targeted antimicrobial activity.

Despite this optimistic outlook, several challenges must be addressed to fully realize MXenes’ potential. Pristine MXenes sometimes exhibit inconsistent antibacterial efficacy, often requiring composite formulation or surface functionalization to achieve consistently potent microbicidal effects. Moreover, concerns about cytocompatibility and long-term safety persist, as some MXenes (especially under certain conditions) can display cytotoxic or off-target effects. These limitations underscore the need for continued research and careful evaluation. Future studies should elucidate the detailed structure–function relationships governing MXenes’ antimicrobial mechanisms, helping to identify how specific structural features or chemistries influence their bactericidal and virucidal performance. Equally important is standardizing MXene synthesis and characterization protocols to improve reproducibility and material consistency across studies. Rigorous *in vivo* evaluations of MXene-based therapies are needed to verify their safety and efficacy in complex biological systems, paving the way for clinical translation. Further exploration of MXenes’ antiviral capabilities and immunomodulatory potential is also recommended, especially given preliminary evidence that certain MXenes can inactivate viruses (e.g., SARS-CoV-2) and modulate immune responses by dampening pro-inflammatory cytokine release.

Overall, with continued innovation and systematic research, MXenes can be custom-tailored as next-generation antimicrobial agents, from smart wound-healing materials and durable implant coatings to water purification membranes, combining tunable nanostructures with targeted antibacterial and antiviral action. By addressing current limitations and emphasizing safety, precision, and mechanism-driven design, MXenes hold the promise of advancing from laboratory breakthroughs to real-world clinical and environmental solutions in the fight against infectious pathogens.
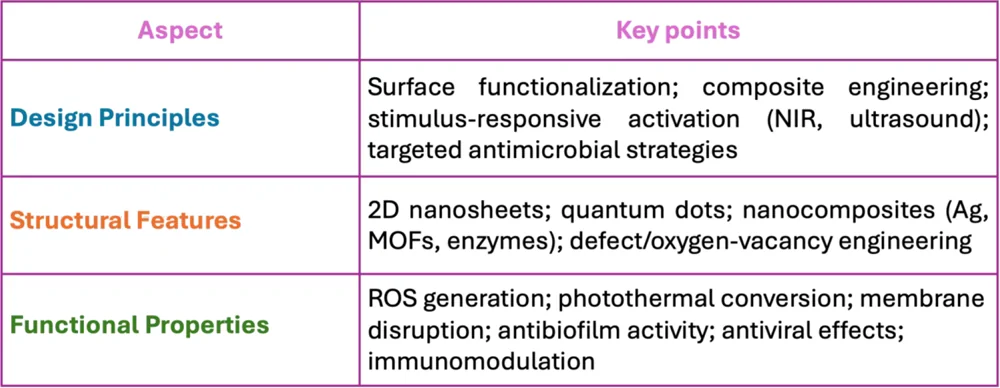


## 8 MXenes for Beyond-Body Biosensing Applications

### 8.1 MXenes in Biosensor Applications

MXenes have also demonstrated promise for use in highly sensitive biosensor-based applications, where detecting or quantifying even low levels of key biomarkers, such as pathogens or chronic disease markers, can support early diagnosis and better treatment outcomes.^288^ Traditional methods for biomolecule detection include high-performance liquid chromatography (HPLC), electrogenerated chemiluminescence (ECL), chemiluminescence, and enzyme-linked immunosorbent assays.^289^ However, these techniques typically require costly protocols, labor-intensive sample preparation, and specialized personnel. As a result, there is considerable interest in developing innovative, cost- and time-effective approaches capable of achieving lower detection limits.^290^ To this end, enzyme-based biosensors are gradually being replaced with biosensors that integrate nanotechnology, artificial intelligence, and information technology to enable more personalized and adaptive healthcare solutions. To enhance biosensor performance, various metals, metal oxides, nitride nanoparticles, and structural nanosheets, including MXenes, have been tested in different biosensor designs.^291^

A typical biosensor includes two main components: a molecular recognition system (bioreceptor) and a physicochemical transducer. The analyte of interest interacts with the bioreceptor at the transducer interface, and the transducer converts this biological response into a measurable electrical or optical signal.^292^ MXene-based biosensors rely on the electrochemical properties of MXenes to facilitate biomolecule detection. Specifically, MXenes function as efficient transducers and can enhance electron transfer between the target biomolecules and the transducer interface. In biosensors that incorporate specific bioreceptors such as aptamers, nucleic acids, proteins, or enzymes, interactions between the bioreceptor and the target molecule produce measurable electrochemical or optical signals. MXenes enhance these signals by increasing their intensity and sensitivity (Figure 10ia).^293^

**Figure 10:**
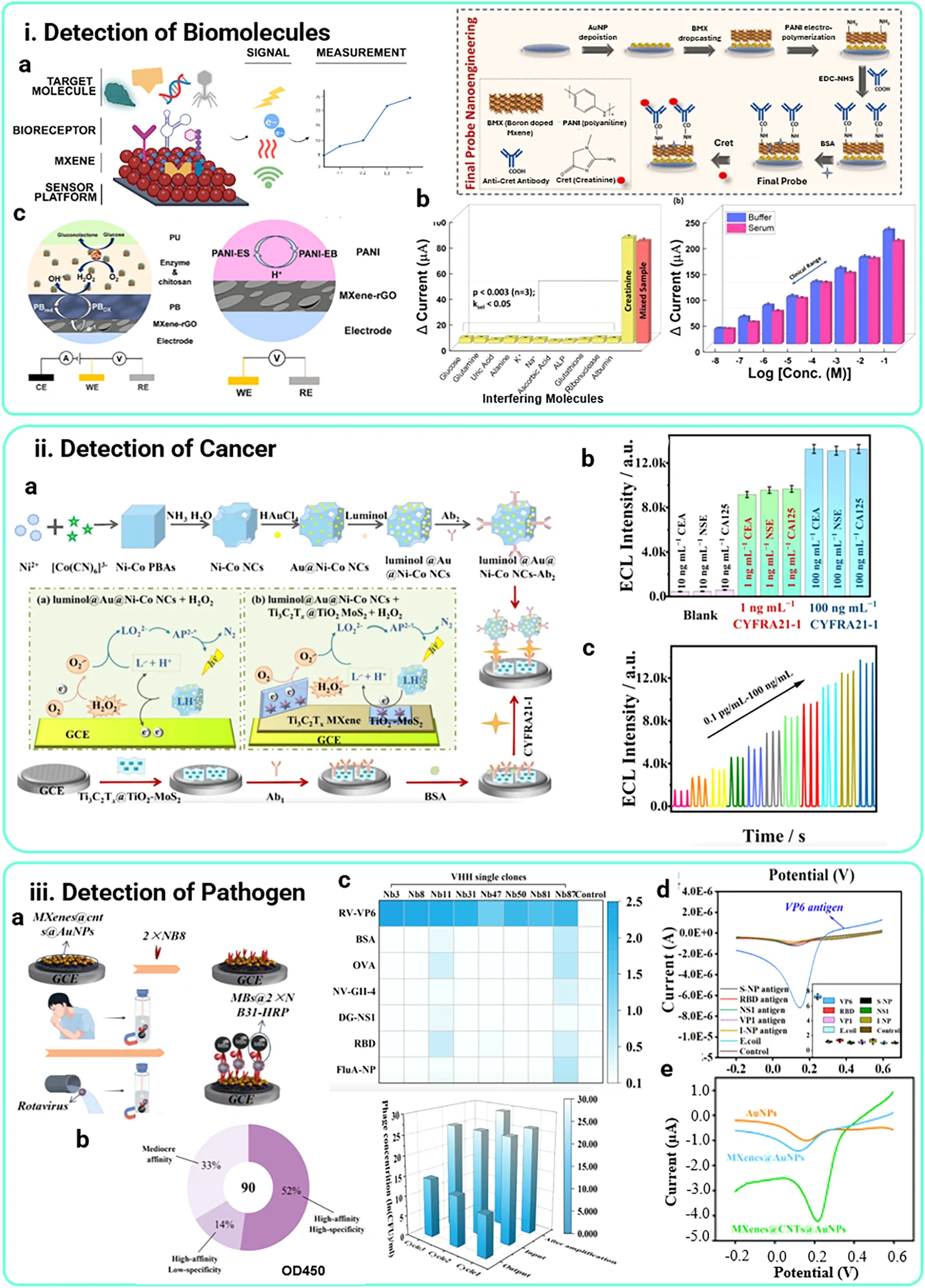
MXenes in biosensor applications. **i**. **Detection of biomolecules**. (a) **Schematic illustration of the biosensor working principle**. The sensor surface is functionalized with biorecognition elements that specifically bind the target analyte, enabling sensitive and selective detection (Created with BioRender.com). (b) The probe was fabricated on a carbon electrode via sequential deposition of AuNPs, PANI, and boron-doped MXene, followed by antibody immobilization. Selectivity tests showed strong responses to creatinine over common serum. Adapted with permission from Divya et al., 2024.^300^ Copyright 2024 American Chemical Society. (c). The glucose sensor uses glucose oxidase (GOx) in a PU–chitosan matrix to generate H_2_O_2_, which reacts with Prussian blue on the MXene–rGO electrode to produce an amperometric signal. The pH sensor operates via proton-driven switching between PANI-ES and PANI-EB, altering the open-circuit potential. Reprinted with permission from Chen et al. 2024.^302^ Copyright 2024 American Chemical Society **ii**. **Detection of cancer**. (a) Schematic illustration of the fabrication of the ECL-based immunosensor. The binding of CYFRA forms a sandwich complex with the ECL probe, generating a strong ECL signal for sensitive detection. (b) The presence of common lung cancer biomarkers did not affect the ECL response to CYFRA, demonstrating high sensor selectivity. (c) ECL intensity increases with CYFRA concentration, showing a strong linear relationship between I_ECL and log CYFRA concentration. Adapted with permission from Ren et al. 2024.^305^ Copyright 2024 American Chemical Society. **iii**. **Detection of Pathogen**. (a) Schematic illustration of the NB-based electrochemical sensor for ultrasensitive rotavirus detection. The sensor is constructed by modifying with MXenes@CNTs@AuNPs and immobilizing NB8 nanobodies for virus capture (b) **Left:** Increasing phage titers across three biopanning rounds indicate enrichment of VP6-binding clones. **Right:** Analysis of 90 clones shows 52% high affinity/high specificity, 14% high affinity/low specificity, and 33% moderate affinity. (c) Strong binding was observed only for RV-VP6, with no significant cross-reactivity to other antigens (d) The sensor showed a strong and selective response to the VP6 antigen, enabling accurate rotavirus detection with minimal false positives. (e) Functionalized electrodes enabled efficient immobilization of nanobodies, enhancing sensitivity. The MXenes@CNTs@AuNPs-modified electrodes generated the highest current response to VP6, confirming superior detection performance. Adapted with permission from Hu et al. 2024.^321^ Copyright 2024 American Chemical Society.

Many biosensors rely on ECL, where electrochemically generated reagents are used in redox reactions to generate light. Because of their electrical conductivity, high surface area, biocompatibility, and catalytic properties, many nanocomposites have been incorporated into electrochemiluminescence-based biosensors to improve sensor performance by facilitating more efficient electron transfer at the receptor/transducer interface.^294^ The effectiveness of a given nanomaterial for biosensor applications depends on its structure and morphology, which are a function of the stabilizer ligands used during nanomaterial synthesis. These ligands can play a critical role in preventing aggregation. However, some stabilizers, such as surfactants, can cover the active sites on the nanomaterial and thereby inhibit electron transfer. As a result, nanomaterials devoid of surfactants, such as MXenes, are preferred for hybridization with metal nanoparticle hybrids because of their exceptional electrical conductivity, electrocatalytic efficiency, and strong reduction abilities.^295^ Given these well-known properties, MXenes are considered promising materials for biosensor applications, particularly in the detection of disease markers and pathogens.

#### 8.1.1 Detection of Biomarkers for Chronic Diseases and Metabolic Disorders

The detection of biomolecular markers in body fluids or biopsy samples can play a critical role in biomedical interventions at all stages of the disease process. Data from biomarker detection methods can be used to diagnose diseases early in their progression, support rapid interventions, monitor disease progression, personalize treatment strategies, and enhance patient comfort.^296^

The performance of biosensors depends on the measurement method (optical, electrical, fluorescence, electrochemical, or mechanical), biosensor components (biomolecules, transducer type, surface chemistry), and sample characteristics (composition, pH, ionic strength). Since all these parameters influence dynamic range, accuracy, sensitivity, signal-to-noise ratio, and sensor stability, they must be considered when comparing detection limits across biosensors.^297^ For example, Yadav et al. achieved a detection limit of 0.5 μM for creatine in serum samples using electrochemical measurements with a platinum electrode in the biosensor they designed.^298^ Sriramprabha et al. reported a serum creatinine detection limit of 144 nM in a nonenzymatic biosensor designed with a hybrid of polyaniline (PANI) and Fe_2_O_3_.^299^ On the other hand, Divya et al. designed an MXene-based biosensor to improve the diagnosis and treatment of chronic kidney disease.^300^ They immobilized a creatinine-specific anticreatinine antibody onto the sensor surface and then measured electrical signals in patient serum samples using differential pulse voltammetry (DPV). The sensor, which achieved a detection sensitivity of 1.72 (±0.07) nM, was further integrated into a smartphone-based mobile application to enable personalized treatment (Figure 10ib). In another study, it was demonstrated that a functionalized MXene with AuNPs (MXene@AuNP) biosensor detected creatinine in serum with a limit of detection of 0.01 mM, in 4 min.^301^

Biosensors also play an important role in diabetes management, where continuous and long-term glucose monitoring systems are essential for maintaining patients’ quality of life. Many electrochemical biosensors for diabetes patients rely on sweat samples, in which accuracy can be significantly affected by pH. Accounting for pH is therefore important when measuring glucose concentrations in sweat. In this context, Chen et al. developed an MXene-based biosensor that could integrate data from simultaneous glucose and pH measurements.^302^ By incorporating polyaniline into the platform, they established a three-dimensional electrode network that enhanced response speed, stability, and selectivity by increasing the number of electrocatalytic active sites (Figure 10ic). The neurotransmitter serotonin can serve as a biomarker for neurologically linked disorders such as cardiovascular dysfunction, anxiety, depression, and obesity. Ilanchezhiyan et al. successfully used a Ti_3_C_2_T_*x*_ MXene nanocomposite containing FeVO_4_ nanoflakes to detect serotonin in serum samples at concentrations as low as 5.88 nM.^303^

#### 8.1.2 Detection of Cancer

Biosensors that can promote early cancer detection also have the potential to revolutionize cancer treatment, as early diagnoses are associated with better disease monitoring, more effective treatment, and higher survival rates.^304^

Cytokeratin-19 (CYFRA 21-1) is a biomarker for the early diagnosis of nonsmall cell lung cancer. In a developed biosensor, CYFRA 21-1 antibody and luminol-loaded gold nanomaterial were hybridized with highly conductive Ti_3_C_2_T_*x*_ MXene@MoS_2_ (Figure 10iia). When applied together with interfering antigens NSE, CEA, and CA125, the biosensor specifically detected CYFRA 21-1 (Figure 10iib). In the biosensor system, the increased O_2_ production resulting from the H_2_O_2_ redox reaction facilitated ECL generation. The ECL signal intensity exhibited a linear relationship with increasing CYFRA 21-1 concentrations, enabling a detection limit of 0.046 pg/mL (Figure 10iic).^305^

Early diagnosis and treatment are particularly crucial to improving cancer patients’ well-being and survival.^306,307^ Recently, advanced diagnostic approaches based on biosensors have been developed for cancer diagnosis,^308,309^ and the past few years have seen increasing interest in the use of MXenes for cancer diagnosis.^310–313^ For example, Li et al. fabricated an optical microfiber biosensor integrated with Ti_3_C_2_T_*x*_-supported gold nanorod hybrid nanointerfaces for the ultrasensitive detection of carbonic anhydrase IX (CAIX) protein and kidney cancer cells.^314^ Their biosensor was able to detect the CAIX protein biomarker at concentrations as low as 13.8 zM in pure buffer solution and 0.19 aM in 30% serum solution, and it could detect kidney cancer cells in cell culture media at a density as low as 180 cells/mL.

Extracellular vesicles known as exosomes have emerged as promising cancer biomarkers, as they mirror the molecular and functional characteristics of their cells of origin. Despite their diagnostic relevance, many studies continue to rely on conventional techniques for exosome detection, isolation, and characterization, as the development of rapid, reproducible, and highly sensitive detection platforms remains challenging.^315^ In one study, Zhang et al. fabricated a biosensor platform using Ti_3_C_2_ MXenes decorated with gold nanoparticles.^316^ No additional reductant or stabilizer was used in their synthesis, and they reported achieving an exosome detection capacity 1,000 times lower than conventional ELISA methods. In their study, MXene acted as both a reductant and an enhancer for the electro-chemiluminescent signal of luminol and the stabilizer. The hydrophilic and metastable properties of MXenes can also lead to rapid oxidation. To address this issue, Wang et al. modified the surface of an MXene-based exosome biosensor with a metal–organic framework.^317^ This system, which prevented direct contact with the target biomolecules while preserving the properties of MXene, successfully captured exosomes carrying programmed death ligand-1 (PD-L1) from the serum of cancer patients. This biosensor was then developed into a rapid and sensitive MXene@MOF/peptides-SPR sensor with a limit of detection of just 5.24 particles/mL.

In cancer diagnostics, MXene-based biosensors are strategically designed based on the principle of combining high electrical conductivity with hybrid nanostructures to amplify detection signals. Structurally, these hybrid interfaces increase the active surface area and facilitate efficient electron transfer. Functionally, this enables the ultrasensitive detection of cancer biomarkers and extracellular vesicles at extremely low concentrations, highlighting their strong potential for early diagnosis and liquid biopsy applications.

#### 8.1.3 Detection of Pathogens

MXenes can be incorporated into pathogen-detecting biosensors to improve screenings for infectious diseases.^318^ In pathogen detection systems, next-generation nanobodies serve as antigen-binding molecules derived from camelids and are characterized by a single antibody domain. These structures offer several advantages over previous detection methods, including their high solubility, stability, batch production capability, smaller antigen-binding regions, and chemical robustness.^319^ Moreover, appropriately targeted nanobodies can be obtained from biological screening methods, thereby eliminating the need for the complex synthesis or immunization protocols required in the preparation of traditional antibodies.^320^ However, exceptional nanoprobes must be selected for precise viral detection. Hu et al. screened a phage-display library to identify nanobodies with a high affinity for the rotavirus VP6 antigen (Figure 10iiia).^321^ They selected and analyzed 90 clones, of which 47 demonstrated high specificity (Figure 10iiib). These clones were further evaluated through an ELISA test to confirm no cross-reactivity with other pathogen-derived antigens (Figure 10iiic). Overall, their fabricated MXene-based sensor was 3.77 × 10^5^-fold more sensitive than traditional immuno-analytical methods, produced fewer false positives, and did not cross-react with other pathogens (Figure 10iiid). This significant enhancement was attributed to the elevated electron flow provided by their composite material (MXenes@CNTs@AuNPs), which was formed from two nanomaterials (Figure 10iiie).

As an alternative to targeting pathogen antibodies, electrochemical biosensors can directly target pathogen DNA. DNA-based pathogen detection methods are rapid, sensitive, and reproducible, but the density of probe DNA used in the receptor can be critical for ensuring detection accuracy. Excessive amounts of probe DNA can yield inaccurate results or low efficiency, while insufficient amounts limit the sensor’s detection capabilities. Fan et al. developed a novel concept for controlling probe DNA density during material synthesis by replacing the surface −OH groups of Ti_3_C_2_T_*x*_ with -NH_2_ groups, thereby forming Ti_3_C_2_NH_2_.^322^ This design reduced the detection limit of hepatitis B viral DNA to as low as 1.05 × 10^–14^ M.
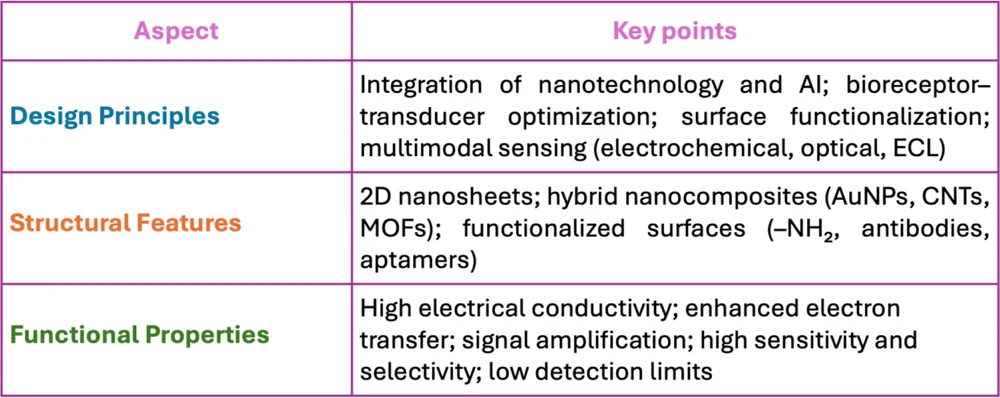


MXenes possess numerous advantageous properties; however, their sensitivity to moisture and susceptibility to oxidative degradation represent certain limitations. These factors may adversely affect their long-term stability, particularly in biosensing applications. To overcome these challenges, various strategies have been developed, including surface modification, coating with polymers or metal nanoparticles, antioxidant stabilization, and the formation of hybrid nanostructures.^3,22,151,182^ These approaches enhance the stability of MXenes while preserving their electrical and catalytic properties, thereby improving their reliability in practical applications. At the same, when assembled into a film, MXenes retain their metallic conductivity over a decade.^323^ Collectively, MXene-based biosensors demonstrate that the synergy between structural properties and the functional integration of biorecognition elements underpins their superior sensing performance. Although significant progress has been achieved, future studies should focus on improving long-term stability, developing scalable fabrication processes, and optimizing surface functionalization strategies to facilitate clinical translation and real-world applications.

Recent studies on MXene-based biosensors are summarized in Table 2.

**Table 2:** MXene-Based Biosensing Studies

	TARGET	MXENE HYBRIDED SURFACE	DETECTION TECHNIQUE	DETECTION LIMIT	REF
BIOMOLECULES	Creatinine	GCE/AuNP/BMX/PANI/Anti-Cret	DPV	1.72 nM	^300^
	Glucose	3D aerogel/Ti_3_C_2_T_*x*_/rGO	Electrochemical	33,69 μM	^302^
	Glucose	Ti_3_C_2_T_*x*_-BPB	ECL	0.02 μmol L^–1^	^5^
	Uric Acid	MIP/CdSe@ZnS/MXene@NaAsc/GCE	ECL/DPV	18.13 pmol L^–1^	^324^
	Dexamethasone	N–TiO_2_/Ti_3_C_2_T_*x*_ -luminol	ECL/Molecular imprinting	2.6 × 10^–7^ μM	^325^
	Dopamine	Ir@Ti_3_C_2_T_*x*_-PVA	ECL	2.0 pmol mL^–1^	^326^
	Alkaline phosphatase	ZnTCPP/Ti_3_C_2_T_*x*_	ECL	0.0083 mU mL^–1^	^327^
	C-reactive protein	3D-MXting (BSA/Al-MXNs@PBA/GA)	AEC	4.2 pg mL^–1^	^328^
	Serotonin	FeVO_4_@Ti_3_C_2_T_*x*_	Electrochemical	5.88 nM	^303^
	Metabolites	MXene@N-EEG	Electrochemical	0.1 nM	^329^
PATHOGENES	SARS-CoV-2-Spike	V_2_CT_*x*_ Mxene	Electrochemical impedance spectroscopy (EIS)	45 fM	^330^
	Hepatitis B	Ti_3_C_2_NH_2_ MXene@Au/pDNA	Electrochemical DPV/EIS	1.05 × 10^–14^ M	^322^
	Hepatitis C	primer@PDA@Nb_2_CT_*x*_ MQDs-Ab	BPE-ECL	3.3 × 10^–5^ ng mL^–1^	^331^
	Rotavirus - VP6	MXenes@CNTs@AuNPs	Electrochemical	0.0207 pg mL^–1^	^321^
CANCER	HER-2	AuPd/NiFeO_*x*_/Ti_3_C_2_T_*x*_	ECL	0.036 pg mL^–1^	^332^
	CYFRA 21–1	Ti_3_C_2_T_*x*_@TiO_2_- MoS_2_	ECL	0.046 pg mL^–1^	^305^
	CA-125 (ovarian)	MXene-GQD/AuNPs	Electrochemical	0.075 nUmL^–1^	^333^
	CEA (colon and rectal)	Ru@TiO_2_-MXene	ECL	2.65 fg·mL^–1^	^334^
	miRNA-373 (breast)	N dots/MoS_2_/MXene	ECL	0.24 fM	^335^
	Exosome	AuNPs/Ti_3_C_2_T_*x*_/CD63 aptamer	ECL	30 particles μL^–1^	^316^
	Exosome	MXene@MOF/peptides	SPR	5.24 particles mL^–1^	^317^

## 9 MXenes as Sustainable Practices for Biomedical Applications

MXenes offer a compelling foundation for advancing sustainability within biomedical technologies because their distinctive chemistry enables the formation of stable aqueous solutions and flexible, processable films, both of which are essential for environmentally responsible biomedical use.^2^ The capacity to prepare MXenes in water, without relying on persistent organic solvents, aligns closely with green biomedical design by reducing chemical burdens during fabrication, patient administration, and disposal. This stability also supports their integration into wearable sensors, implantable coatings, and transdermal or mucosal delivery platforms, where material compatibility with soft tissues and body fluids is crucial.^336^ In such applications, MXenes’ intrinsic flexibility and hydrophilicity enable thin and energy-efficient devices, thereby lowering the operational footprint of continuous health monitoring systems.

From a resource perspective, many widely studied MXenes are based on relatively abundant transition metals such as titanium, niobium, and vanadium, offering a more sustainable alternative to noble-metal or rare-element components traditionally used in biomedical electronics.^2^ This abundance contributes to long-term material accessibility for medical technologies,increasingly important consideration as global demand for electronic and biosensing platforms grows. At the same time, the biomedical sector must address the sustainability implications of single-use materials, which dominate clinical practice for safety and sterility reasons. MXene-based biosensors, wound dressings, and drug-delivery patches therefore benefit from design strategies that minimize material mass, incorporate biodegradable or low-toxicity matrices, and enable safe disposal to prevent environmental accumulation.^267^

Collectively, these attributes position MXenes as a forward-looking material class in which sustainability is achieved not through production processes alone, but through thoughtful integration into biomedical contexts—considering material abundance, device longevity, waste generation, and ecological safety at each stage of clinical use.
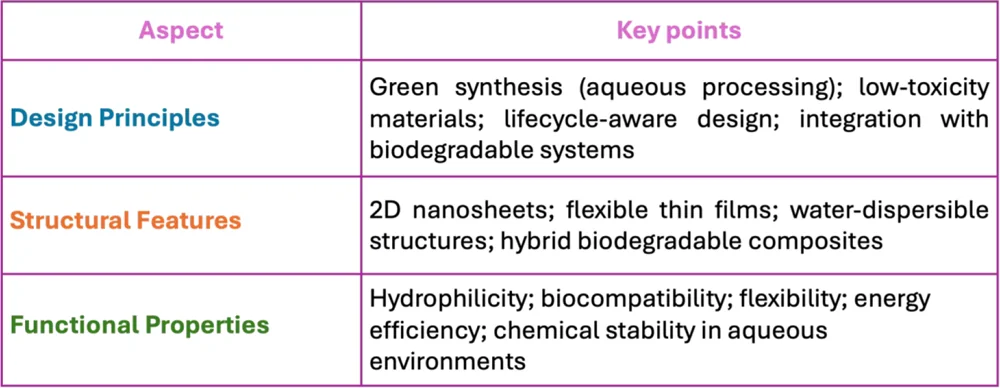


## 10 Artificial Intelligence for MXenes in Healthcare

The integration of artificial intelligence (AI) and machine learning (ML) into MXene research is emerging as a powerful strategy to accelerate both materials discovery and biomedical translation. At the materials level, AI enables the identification of structure–process–property relationships and supports inverse design by analyzing large experimental and computational data sets. For example, a recent *Nature Communications* study reported a machine-learning-assisted robotic platform for optimizing Ti_3_C_2_T_*x*_ ***-*** based aerogels, where active learning accelerated the discovery of compositions with targeted mechanical and electrical properties.^337^ More broadly, ML models can predict key MXene attributes, including electrochemical performance, mechanical strength, thermal stability, and cytotoxicity, while also optimizing synthesis parameters such as temperature, etchant type, and precursor ratios, thereby improving reproducibility and reducing empirical trial-and-error.^338,339^ AI-driven tools further streamline characterization: convolutional neural networks and related approaches enable rapid interpretation of spectroscopic data and real-time monitoring of structural evolution, correlating surface chemistry, defects, and oxidation states with functional performance.^339^ These capabilities are particularly important for biointerfaces, where MXene biocompatibility and stability depend on dynamic physicochemical changes in biological environments.

At the device and biomedical application level, AI is increasingly integrated with MXene-based systems to enable intelligent sensing, diagnostics, and therapeutic design. MXene wearable sensors combined with embedded ML algorithms have demonstrated real-time motion classification and human–machine interfacing,^340^ while more advanced platforms integrate multimodal sensing with on-device data processing, highlighting opportunities in neuromorphic and adaptive bioelectronics.^341^ In diagnostics, AI-enhanced MXene biosensors can extract clinically relevant information from complex signals; for instance, a DNA-functionalized MXene gas sensor array coupled with ML enabled accurate classification of multiple cancer types from breath samples.^342^ Beyond sensing, AI facilitates the design of MXene-based drug delivery systems and biomaterials by predicting biomolecular interactions, screening for biocompatibility, and optimizing parameters such as drug loading, targeting specificity, and degradation profiles using techniques like Bayesian optimization and reinforcement learning^343^ (Figure 11). Despite these advances, the application of AI in MXene biomedicine remains at an early stage. Current studies are often proof-of-concept and limited by the lack of standardized data sets, cross-platform validation, and clinically representative data. If successfully realized, the convergence of MXene materials science with AI-driven analytics could transform MXenes into intelligent, adaptive platforms for next-generation diagnostics, therapeutics, and bioelectronic systems.
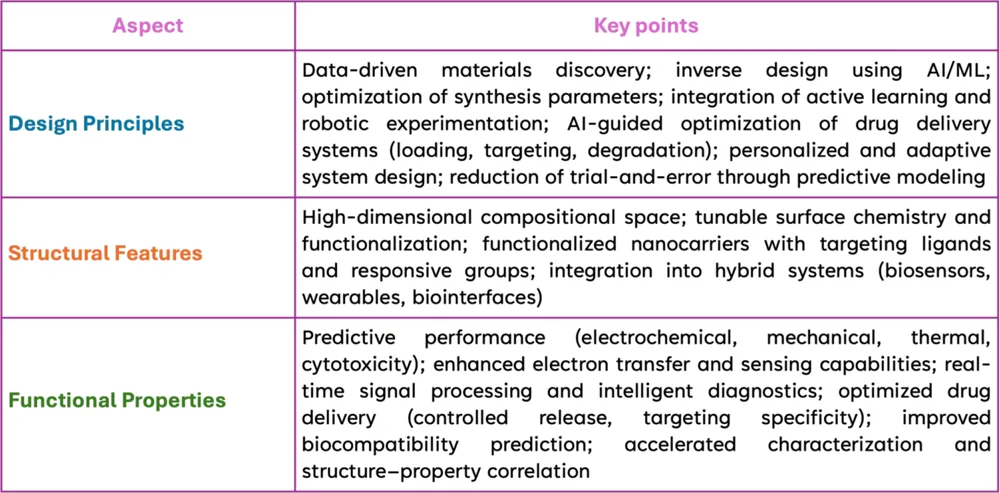


**Figure 11:**
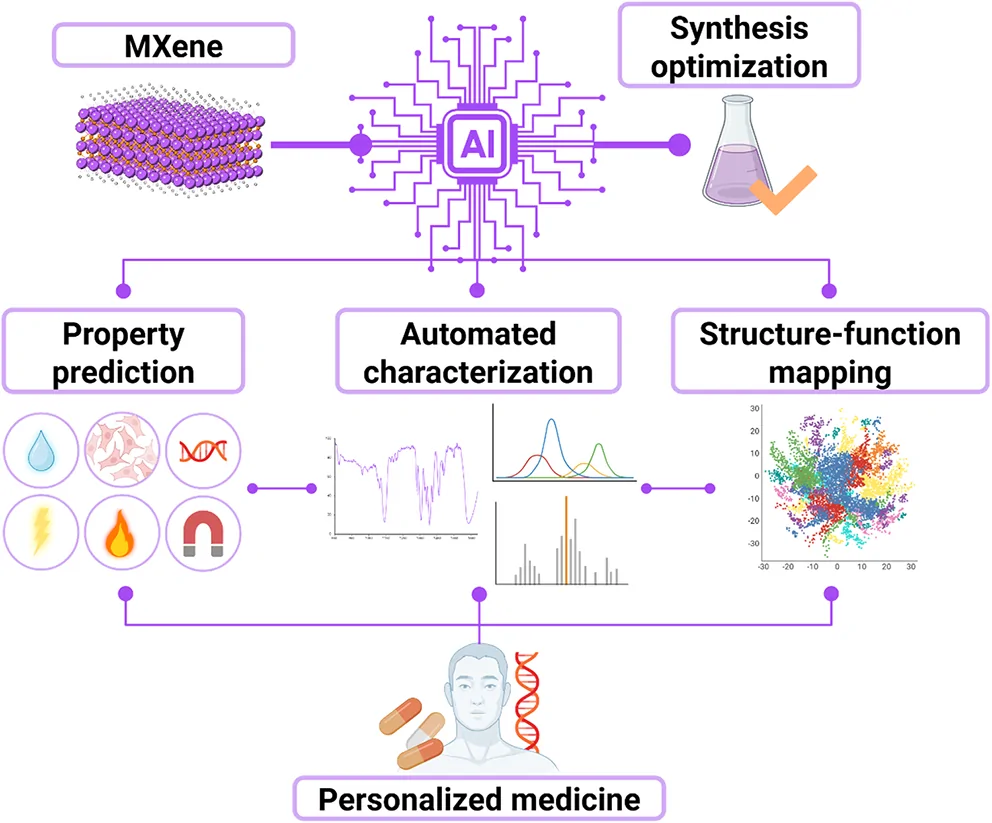
Smart MXenes: AI-enabled property prediction, synthesis optimization and personalized medicine. The figure illustrates the role of AI in accelerating MXene-based material design and application. AI algorithms support synthesis optimization by identifying optimal reaction conditions and precursors and facilitate automated characterization through rapid analysis of complex spectroscopic and imaging data. These data streams enable structure–function mapping, linking compositional and morphological features to material performance. AI further aids in property prediction, encompassing hydrophilicity, mechanical behavior, electronic and magnetic responses, and bioactivity. The convergence of these tools enables the design of tailored MXene-based systems for personalized medicine, enhancing therapeutic efficacy and patient-specific treatment strategies. (Created with BioRender.com).

## 11 MXenes vs Conventional Materials

The trajectory of graphene oxide (GO) in biomedicine provides a particularly instructive benchmark for the clinical translation of MXenes. Graphene-based nanomaterials have been explored for over two decades, yet this extensive preclinical effort translated only recently into a first in-human inhalation study, where highly purified GO nanosheets were acutely well tolerated in healthy volunteers, with no significant cardiopulmonary adverse effects and only minor transient changes in one blood parameter.^344^

This milestone highlights a key lesson for MXenes: early safety signals, while encouraging, must be complemented by rigorous long-term evaluation of biodistribution, clearance, persistence, and immunological effects, which remain major barriers in nanomedicine translation.^345^

GO research has further demonstrated that biological responses are highly dependent on physicochemical parameters, such as lateral size, thickness, aggregation state, oxidation degree, and impurity profile. Larger GO sheets, for instance, induce more persistent pulmonary inflammation and granuloma formation compared to smaller flakes, underscoring the importance of size control and clearance kinetics.^346,347^ In addition, persistent translational bottlenecks, including batch-to-batch variability, lack of standardized characterization, contamination, and limited reproducibility, have been identified as key challenges slowing clinical progression.^348^ These insights provide a concrete roadmap for MXene development. “Safe-by-design” strategies should prioritize precise control over flake dimensions, colloidal stability, surface terminations, oxidation susceptibility, and residual synthesis byproducts, alongside standardized reporting and reproducibility. At the same time, clinical evaluation should incorporate exposure-route-specific toxicology, realistic dosing, and long-term follow-up frameworks aligned with regulatory expectations. In this context, GO serves not only as proof that 2D nanomaterials can reach human testing, but also as a cautionary example that material definition, biological fate, and manufacturability must be addressed early to enable successful translation.

Beyond safety considerations, both GO and MXenes have emerged as important platforms for bioelectronic interfaces due to their mechanical flexibility, solution processability, and compatibility with scalable fabrication methods, enabling applications in neural interfaces, wearable electronics, and electrophysiological recordings.^349,350^ GO benefits from abundant oxygen-containing functional groups that facilitate dispersion and biofunctionalization; however, these same groups disrupt the sp^2^ carbon network, resulting in reduced electrical conductivity and increased interfacial impedance, often requiring reduction or composite strategies that introduce heterogeneity.^351^

In contrast, MXenes uniquely combine hydrophilic, termination-rich surfaces with metal-like electronic conductivity, enabling strong and stable tissue–electrode coupling without extensive postsynthetic modification.^2^ This results in lower interfacial impedance, higher charge-injection capacity, and improved signal fidelity compared to GO-based electrodes.^20,352^ Furthermore, MXenes exhibit mixed ionic–electronic transport, which is particularly advantageous in biological environments, allowing efficient transduction between ionic signals and electronic readouts.

Compared with semiconducting materials (e.g., MoS_2_), MXenes offer orders-of-magnitude higher electrical conductivity and faster charge transfer kinetics, while still maintaining a layered structure that supports ion intercalation.^353,354^ Relative to traditional pseudocapacitive materials such as transition-metal oxides, MXenes exhibit superior rate capability and cycling stability owing to their robust inorganic framework and low internal resistance, while still enabling redox-active behavior through surface terminations and interlayer chemistry.^2^ Additionally, their relatively large and tunable interlayer spacing provides efficient ion transport pathways that are less accessible in restacked graphene orin densely packed oxide structures.^352,355^

Furthermore, Gogotsi and Anasori^151^ and subsequent studies have shown that Ti_3_C_2_T_*x*_ MXenes exhibit metal-like conductivity and superior charge transport compared to GO. Compared to conductive polymers such as PEDOT:PSS, MXenes provide higher intrinsic conductivity and improved electrochemical performance, while retaining solution processability and flexibility, as discussed in the study of Driscoll et al.^20^ However, MXenes remain more susceptible to oxidation and long-term degradation than graphene-based materials.^356^ In contrast, conventional metals such as gold and platinum offer superior long-term stability and well-established clinical validation, particularly in implantable devices, as reviewed by Green et al.^357^ Nevertheless, these materials lack the mechanical flexibility, surface tunability, and mixed ionic–electronic transport that distinguish MXenes and enable advanced soft biointerfaces.

Collectively, these characteristics position MXenes not as replacements for GO or other material classes, but as complementary next-generation platforms that overcome key limitations in conductivity and interface performance while introducing new challenges related to stability and standardization. As demonstrated by the graphene field, their successful clinical translation will ultimately depend on rigorous material control, reproducible manufacturing, and comprehensive *in vivo* validation.
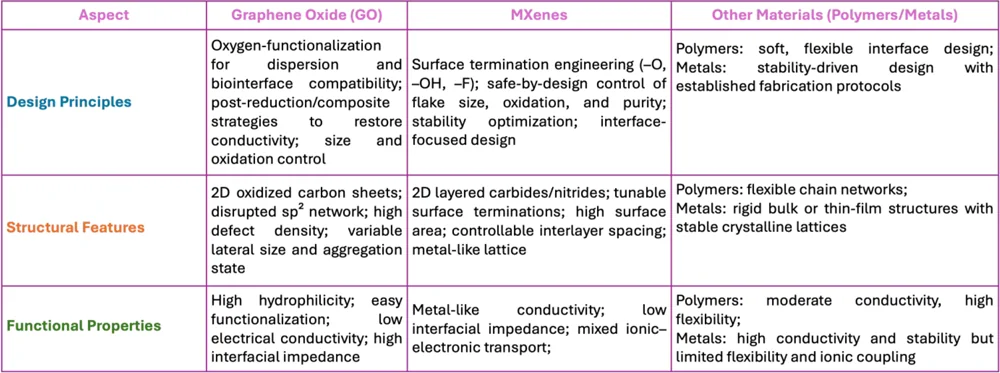


## 12 Conclusions

MXenes are a rapidly emerging family of 2D nanomaterials with tremendous promise in biomedical applications. Their metallic conductivity, large surface area, and tunable surface chemistry enable diverse functionalities from therapeutics to diagnostics. At the same time, MXenes have emerged as key materials for wearable, epidermal, and implantable bioelectronics, where their low interfacial impedance, hydrophilicity, and mechanical flexibility enable intimate coupling with soft tissues. MXene-based electrodes and sensors have demonstrated superior electrophysiological performance compared with conventional materials, supporting long-term, gel-free monitoring and stimulation in neural, cardiac, and muscular systems.^18,20,47^ These capabilities position MXenes as foundational components for next-generation wearable and implantable platforms that bridge biological systems with digital health technologies. In regenerative medicine, MXenes and their composites hold *great promise* as components of scaffolds and implants, offering high mechanical strength and electrical conductivity to stimulate tissue repair.^358^ Recent studies also highlight their ability to modulate macrophage polarization and reduce inflammatory responses, expanding their role in immuno-regenerative therapy.^63^ MXenes have also become rising stars in cancer therapy research. Early studies established Ti_3_C_2_T_*x*_ MXene as an effective NIR photothermal agent for tumor ablation^153,170^ benefiting from strong NIR absorption and inherent biocompatibility and biodegradability.^359^ Building on this foundation, later works combined MXene-based PTT with PDT, chemotherapy, or immunotherapy—taking advantage of MXenes’ capacity to carry therapeutic payloads and transduce energy.^47,248^ In radiotherapy, delivering MXene sheets into tumors can boost the DNA damage and oxidative stress induced by X-rays. In fact, MXene nanomaterials achieved greater ROS generation under irradiation than various other nanomaterials,^360^ underscoring their potential advantage as radiosensitizers. As antimicrobial agents, MXene nanosheets have demonstrated broad-spectrum bactericidal effects through multiple mechanisms—physically disrupting microbial membranes, generating ROS, and converting light to heat for photothermal disinfection.^361^ Such multipronged strategies could help combat antibiotic-resistant infections. Beyond therapeutics, MXenes are making inroads in biomedical diagnostics. Thanks to their high conductivity and rich surface chemistry, MXene-based biosensors can detect clinically relevant biomarkers—from proteins and nucleic acids to metabolites and pathogens with ultralow detection limits and excellent signal stability.^362^ Altogether, the successes of MXenes in tissue regeneration, cancer treatment, antimicrobial therapy and diagnostics underscore their potential to become keystone materials in next-generation biomedicine.

## 13 Gaps in Knowledge and Key Directions in MXene-Based Applications

Despite rapid progress in MXene research, several fundamental challenges continue to limit their scalable implementation and clinical translation. A primary concern is the chemical and structural stability of MXenes during synthesis and processing. Many MXenes remain vulnerable to oxidation under harsh chemical environments or elevated temperatures, often leading to the formation of hybrid oxide–MXene or carbon–MXene structures that alter intrinsic properties.^3,22,151,182,323^ Although recent advances—including antioxidant stabilization, edge passivation, and stoichiometric control—have substantially improved oxidation resistance, such as extending the aqueous stability of Ti_3_C_2_T_*x*_ to nearly one year through glutathione-mediated protection.^363^ These strategies are not yet universally applicable across MXene chemistries. Furthermore, while emerging synthesis routes based on molten salts, termination exchange, and halogen etching have expanded the compositional landscape to include high-entropy MXenes, reproducible control over surface terminations, defect density, and morphology—particularly at scale—remains a significant challenge.^3^ Material purity and structural uniformity present additional bottlenecks. Residual MAX phases, unremoved etching byproducts (e.g., LiF, AlF_3_), and secondary carbide or nitride phases can influence physicochemical and biological behavior, limiting reproducibility. As a result, rigorous purification protocols and standardized characterization workflows are essential to distinguish intrinsic MXene properties from impurity-driven effects.^3^ From a device-engineering perspective, scalable routes to large-area, single-crystal or monolayer MXenes remain elusive. Vapor-phase approaches have not yet achieved uniform, defect-controlled MXene films, restricting fundamental studies of electronic band structure and limiting integration into nanoelectronic and optoelectronic platforms.^3^ Computational modeling has played a pivotal role in predicting MXene properties, and the integration of machine learning is increasingly accelerating materials discovery and process optimization. However, many theoretically predicted phenomena—including ferromagnetism, topological insulating behavior, and extreme transport properties—have yet to be experimentally validated, underscoring a persistent gap between theory and experiment.^364^ In parallel, biomedical translation faces distinct challenges. The widespread use of toxic fluoride-based etchants, particularly HF, remains a major barrier to regulatory approval. Although short-term in vitro and acute in vivo studies generally indicate favorable biocompatibility, comprehensive long-term assessments addressing chronic exposure, biodistribution, degradation pathways, and organ accumulation are still lacking. Importantly, MXene toxicity and immune responses are highly dependent on composition, surface terminations, oxidation state, and dose, precluding broad generalization across the MXene family.^356,365,366^

Looking forward, addressing these challenges will require coordinated advances across synthesis, characterization, modeling, and translational research. Establishing predictive structure–property–biological response relationships that integrate MXene chemistry, surface functionality, and defect landscapes is essential to guide rational material design. Long-term in vivo studies under clinically relevant conditions are needed to define biosafety profiles, immune interactions, biodegradation mechanisms, and clearance routes. In regenerative medicine and bioelectronics, mechanistic studies must disentangle the relative contributions of electrical conductivity, redox activity, photothermal effects, and mechanical compliance to therapeutic outcomes. For antimicrobial and anticancer applications, improved targeting strategies and precise spatiotemporal control over ROS generation will be critical to maximize efficacy while minimizing off-target toxicity. From a manufacturing perspective, the transition toward scalable, fluorine-free, and oxidation-resistant synthesis routes aligned with green chemistry and Good Manufacturing Practice standards is fundamental.^367^ The integration of artificial intelligence and high-throughput computational screening offers powerful opportunities to accelerate material discovery and optimize synthesis parameters. Ultimately, successful clinical translation will depend on standardized production protocols, well-defined dose and exposure metrics, cleanroom-compatible manufacturing for injectable and implantable systems, and rigorous validation in large-animal models to meet regulatory requirements established by agencies such as the Food and Drug Administration (FDA) and European Medicines Agency (EMA) (Figure 12).

**Figure 12:**
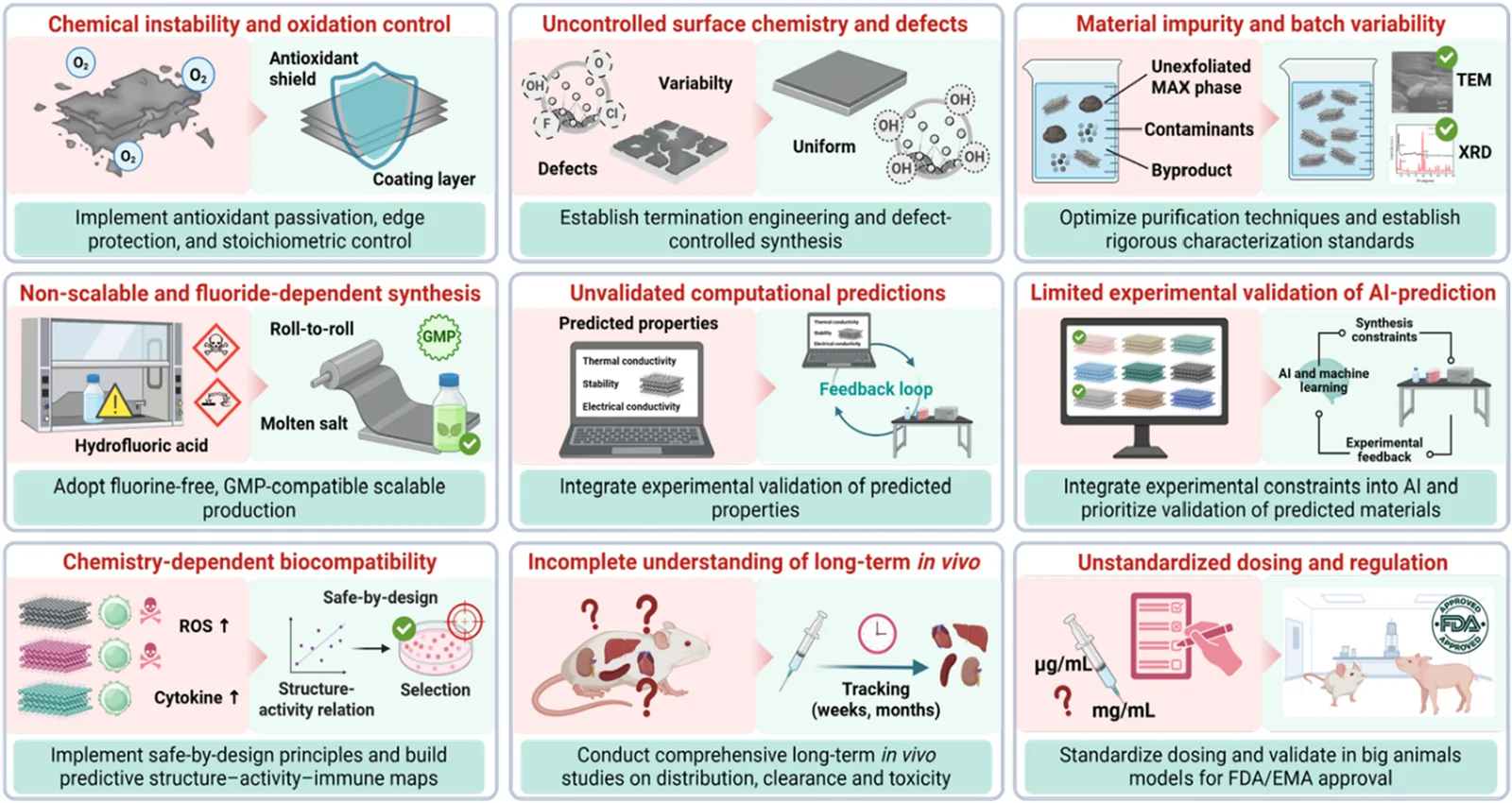
Knowledge gaps and key directions in MXene research in biomedicine. Schematic overview highlighting major priorities in MXene research. The figure is organized from materials-related challenges (top) to synthesis and validation bottlenecks (middle), and finally to biological and regulatory considerations (bottom). For each identified gap, a concise strategic direction is highlighted, providing a roadmap to guide future research toward scalable production, reliable prediction and validation, improved biocompatibility, and regulatory readiness. (Created with BioRender.com).

## Vocabulary

MXenes: MXenes are a family of two-dimensional transition-metal carbides, nitrides, and carbonitrides with the general formula M_*n*+1_X_*n*_T_*x*_, characterized by high electrical conductivity, tunable surface chemistry, and mixed ionic–electronic transport, making them highly versatile for biomedical applications.
Theranostics: Theranostics describes the integration of therapeutic and diagnostic functions within a single MXene-based platform, enabling simultaneous tumor treatment (e.g., PTT or SDT) and real-time imaging monitoring.
Reactive oxygen species (ROS) generation: ROS generation refers to the controlled production of highly reactive oxygen species by MXene-based systems under external stimuli (NIR light, ultrasound, or radiation), inducing oxidative stress and apoptosis in cancer cells.
Immunomodulation: Immunomodulation refers to the capacity of MXenes to regulate immune responses, for example, by influencing macrophage polarization (M1/M2 balance), thereby reducing inflammation and promoting tissue regeneration.
NIR-II phototherapy: NIR-II phototherapy refers to tumor treatment using second near-infrared window irradiation, which enables deeper tissue penetration and improved photothermal performance of MXene-based scaffolds for cancer ablation.
